# A novel method for detecting morphologically similar crops and weeds based on the combination of contour masks and filtered Local Binary Pattern operators

**DOI:** 10.1093/gigascience/giaa017

**Published:** 2020-03-04

**Authors:** Vi Nguyen Thanh Le, Selam Ahderom, Beniamin Apopei, Kamal Alameh

**Affiliations:** Electronic Science Research Institute, Edith Cowan University, 270 Joondalup Drive, Joondalup, Western Australia, 6027

**Keywords:** precision agriculture, morphological operators, feature extraction, local binary patterns, contour masks, weed detection, computer vision

## Abstract

**Background:**

Weeds are a major cause of low agricultural productivity. Some weeds have morphological features similar to crops, making them difficult to discriminate.

**Results:**

We propose a novel method using a combination of filtered features extracted by combined Local Binary Pattern operators and features extracted by plant-leaf contour masks to improve the discrimination rate between broadleaf plants. Opening and closing morphological operators were applied to filter noise in plant images. The images at 4 stages of growth were collected using a testbed system. Mask-based local binary pattern features were combined with filtered features and a coefficient *k*. The classification of crops and weeds was achieved using support vector machine with radial basis function kernel. By investigating optimal parameters, this method reached a classification accuracy of 98.63% with 4 classes in the “bccr-segset” dataset published online in comparison with an accuracy of 91.85% attained by a previously reported method.

**Conclusions:**

The proposed method enhances the identification of crops and weeds with similar appearance and demonstrates its capabilities in real-time weed detection.

## Introduction

Weed infestation poses a threat to the environment, crop yields, and quality. Weeds in a field retard crop growth by competing for access to sunshine, water, and nutrients. In particular, the density, spreading time, and growth characteristics are important factors for weed management [1]. One of the most invasive and serious weeds is wild radish, which causes significant crop yield losses and low-quality crops owing to its fast growth rate, contaminants, multiple-herbicide resistance, and vigorous competition [2–4]. Currently, blanket herbicide spraying is the most common practice used to eradicate weeds. However, the excessive use of herbicides has negative effects on the environment in addition to the development of herbicide resistance properties in weeds. The dramatic challenge for controlling weeds is to attain an optimal eradication efficacy with minimum herbicide usage. Note that reducing herbicide application rates decreases the cost of weed management. Hence, it is a worthwhile objective in precision agriculture.

Spraying selected weeds automatically in vegetation fields is considered as a potential method to reduce the environmental and economic costs of weed management. Wild radish is a dominant weed in all broadacre field crops, including wheat, barley, sorghum, maize, and canola. Canola is the most difficult crop to discriminate against wild radish because of their morphological similarity [5]. Therefore, canola, corn, and wild radish are selected for experimental investigation in this study. Classifying crops and wild radish plants is a vital practical problem in agriculture. The ability to accurately detect and classify weeds in row crops in real time enables the selective application of herbicides, thus enhancing the quality and productivity of crops.

There have been numerous studies on weed-from-crop discrimination. Spectral techniques based on the calculation of the normalized difference vegetation indices (NDVIs) [6, 7] have long been proposed for identifying plant species. However, this method has some deficiencies. In typical farm field conditions, the wind, shadowing, and soil background brightness may change the spectral features of plants, leading to a reduction in the discrimination accuracy of NDVI-based weed sensors [8, 9]. Owing to the drawbacks of such spectral reflectance sensors, research on spatial sensors based on the use of image-processing techniques for the classification of plant species and weeds in real time has been conducted [10]. One such spatial technique is “texture analysis” in image processing, which has been applied in many fields, such as industrial inspection systems, medical image analysis, face recognition, and content-based image retrieval [11]. There are significant challenges in image texture analysis, such as noise sensitivity, grey-scale variation, rotation sensitivity, and illumination and brightness conditions. One of the discriminative and computationally effective local texture descriptors that can potentially overcome these issues is local binary patterns (LBP) [12–14]. The important role of extracting dominant features is emphasized because poor features combining with even the best classifier are unlikely to achieve good identification results.

In this article, the LBP method is applied to extract plant features owing to its flexibility and robustness in monotonic grey-level transformation, illumination, scaling, viewpoint, and rotation variance. Furthermore, the LBP method is also a robust tool for identifying the relationship among the pixels in plant images and detecting microstructures including lines, spots, edges, and flat areas [14]. Another attractive feature of the LBP method is low computational complexity [15]. In fact, the LBP is computationally less complex than its SIFT or SURF counterparts [16]. Finally, it has exhibited superior performance in various applications, such as motion analysis [17, 18], texture recognition [12, 14, 19], face recognition [20–22], face expression analysis [23, 24], fingerprint recognition [25], and image retrieval [26, 27].

Numerous studies on the LBP method have been developed to enhance its discriminative power including completed LBP [12], extended LBP [28, 29], discriminative completed LBP [30], dominant LBP with Gabor filtering features [19], pairwise rotation invariant co-occurrence LBP [31], fuzzy LBP [32], robust LBP [33], noise-tolerant LBP [34], and noise-resistant LBP [35]. However, these methods still have unsatisfying tolerance to noise in images and increased feature dimensionality, leading to high computational complexity [36].

In the agricultural context, the complex and similar morphologies of plant leaves are one of the key challenges to finding effective and discriminative plant descriptors. Combining LBP features with other features from different methods has become an interesting research topic in plant recognition. There have been several approaches based on applying the LBP method for the identification and classification of plants. For example, using LBP, in conjunction with template matching and support vector machine (SVM), was proposed to classify broadleaf and grass weed images [37]. These weed images having broad and narrow leaf shapes were easily distinguished. Similarly, another study combined LBP, local ternary pattern, and local directional to classify broadleaf and narrow grass weeds [38]. Another statistical method for separating sugar beets and weeds has been proposed, based on using shape features [39]. However, this method was considered accurate only because the sugar beet sizes were significantly different from those of the weeds. The LBP method has also been used for crop segmentation to detect occluded crops (sweet pepper) [40]. However, the detection accuracy was limited (just 67%). The detection and classification of apple fruit diseases using global colour histogram, colour coherence vector, LBP, and complete LBP has been investigated [41]. The classification accuracy of this method was 93%. Identifying medicinal plants was conducted by combining morphological, LBP variance, and colour features, and the classification accuracy of this method was 72% [42]. In addition, canola, corn, and radish plants have been classified using the combined LBP operators and SVM with a classification accuracy of 92% [43]. These methods are still deemed unsatisfactory owing to their low classification accuracy.

Some studies have investigated a promising approach to reducing noise and increasing classification accuracy: the combination of the LBP operators and contours that mask LBP images. LBP-guided active contour approaches have only been proposed for texture segmentation [44]. The active contour can identify the position of the initial curve anywhere in the captured image and then automatically detect interior contours. By combining scalar and vector LBP active contours, reduced computational cost and high segmentation quality can be achieved. However, typically, this method has been applied in the segmentation process. LBP-based edge-texture features for object recognition has also been proposed [45]. Particularly, discriminative LBP (DLBP) and local ternary pattern (DLTP) were focused on differentiating a bright object against a dark background by combining edge and texture information. Another method for detecting humans based on non-redundant LBP shape descriptor has been implemented by concatenating a set of local appearance descriptors extracted at a set of key points. However, occlusion was the main limitation that made this method impractical [46]. Another LBP edge-mapped descriptor for face recognition has been investigated [47], whereby LBP was applied on the edge contours (eyes, nose, and mouth) instead of the whole image; then the LBP intensity was combined with the edge pixel array around the feature points.

The aforementioned methods have their own drawbacks, such as unsatisfactory classification accuracy, computational complexity, application-specific recognition, and not dealing with occlusion. In the context of this article, we address the challenge of discriminating broadleaf plant species of relatively similar morphology by proposing a novel method called “filtered LBP method with contour mask and coefficient *k* (k-FLBPCM),” which enhances plant discrimination capability. The k-FLBPCM is based on combining filtered LBP features and contour mask–based features to precisely identify and classify broadleaf plants in the field. The present k-FLBPCM method has particularly been applied for the classification of 2 broadleaf plants, namely, canola (crop) and wild radish (weed), which significantly improves on the accuracy of our previously published article [43]. This article still uses an SVM classifier owing to its good accuracy and relevance to real-life datasets [48, 49]. We use the “bccr-segset” dataset, which comprises a variety of plant images at 4 defined growth stages, with rotation, scale, and viewpoint variance, to compare the present results with our previously reported results.

## Morphological Operations

The excess green minus excess red indices (ExG − ExR) method was used to segment green plant regions in the bccr-segset dataset [43]. During segmentation, the noise in plant images creates issues in the process of edge detection. However, reducing the noise level in these plant images plays an important role in image enhancement for the next stages of feature extraction and classification.

Morphological image processing is particularly investigated in this article [50]. Morphological operators are introduced and extended to analyse images by Serra [51]. Particularly, in morphological analysis, images are treated as sets that illustrate the plant shapes, represented in grey-scale or binary images. Morphological transformations are a tool that helps extract features from images using Minkowski addition and subtraction [52]. The morphological process needs 2 inputs including grey-scale images and structuring elements. The function of morphology operators is to transform from one set to another with the aim of searching the special structure of the original set. Then, the special structure information is stored in the transformed set and the transformation is recognized by special structuring elements. As a result, there is a correlation among some characteristics of the structuring elements.

There are 2 basic morphological operations for binary and grey-scale images: erosion and dilation. Erosion is defined as a shrinking transformation, which reduces the size of regions within the image while expanding the size of holes within the regions. Dilation is defined as an expansion transformation, which increases the size of regions within the image while reducing the size of the holes in the regions and gaps between the regions. It is important to note that the erosion operator filters the inner image, while the dilation operator filters the outer image. Opening and closing morphological operators, which are an extension of erosion and dilation operators, are also used, to find specific shapes in an image. Specifically, the opening operation comprises the erosion operation followed by the dilation operation; it helps to smooth the contour of an image and eliminate small objects. On the other hand, the closing operation tends to remove small holes and fill gaps in the contours [53]. Note that morphological operations have gained popularity because they are useful for the detection of the edge of an image and suppression of noise.

In this article, opening and closing morphological operators are applied on grey-scale images, mainly to filter noise [53], while erosion and dilation operations are used for processing image edges. *I*(*x, y*) is considered as a grey-scale 2D image, and *S* is a structuring element. The erosion of a grey-scale image *I*(*x, y*) by a structuring element *S*(*a, b*) is defined as [52, 54] follows:

(1) $$\begin{equation*}
I \ominus S = {\rm{min}}\left\{ {I\left( {x + a,{\rm{y}} + b} \right) - S\left( {a,b} \right)} \right\}.
\end{equation*}$$

The dilation of a grey-scale image, *I*(*x, y*), is denoted by

(2) $$\begin{equation*}
I \oplus S = {\rm{\ max}}\left\{ {{\rm{I}}\left( {x - a,y - b} \right) + S\left( {a,b} \right)} \right\}.
\end{equation*}$$

Based on the erosion and dilation operators, the opening and closing of the image *I* by the structuring element *S* are respectively defined as follows:

(3) $$\begin{equation*}
I \circ S = \left( {I \ominus S} \right) \oplus I,
\end{equation*}$$

(4) $$\begin{equation*}
I \cdot S = \left( {I \oplus S} \right) \ominus S.
\end{equation*}$$

In this article, the first step is to select structuring elements, which are regarded as matrices and able to measure the shape of the image. In addition, choosing the shape and size of the structuring element is based on the condition and processing demand of the image. We used a 5 × 5 square structuring element to input the opening and closing morphological operators for filtering. The opened and closed images were then converted to binary images by using thresholds for next features extraction and classification processes.

## Local Binary Pattern Operators

The LBP algorithm was introduced by Ojala et al. 1996 [55]. The LBP operator has been developed to detect textures or objects in images for a long time. It is considered a robust texture descriptor for analysing images because of its capability to represent plant discriminative information and computational efficiency [55]. It is also one of the best texture descriptors and has been effectively used in various applications. The potentials and effectiveness of LBP have been presented in identifying objects, recognizing faces and facial expressions, and classifying demographic characteristics. In this article, the LBP operator is particularly used for leaf description owing to its effectiveness in pattern description.

The main limitation of the previously reported LBP operator was to cover only a small 3 **×** 3 neighbourhood, thus failing to capture dominant textural features in images with large-scale structures. To overcome this drawback (i.e., improve the LBP operators), the number of pixels and the radius in the circular neighbourhood have been increased [14]. Typically, it is more flexible and effective to enhance the performance of the LBP method by using textures of different scales. Generally, the value of the LBP code of a centre pixel (*x_c_,y_c_*) can be calculated as follows [14]:

(5) $$\begin{equation*}
{\rm{LB}}{{\rm{P}}_{P,R}} = \mathop \sum \nolimits_{p = 0}^{P - 1} s\left( {{g_p} - {{\rm{g}}_{\rm{c}}}} \right){2^p}{\rm{\ \ where\ }}s\ \left( x \right) = \left\{ {\begin{array}{@{}*{1}{c}@{}} {1,x \ge 0}\\ {0,x < 0} \end{array}} \right.
\end{equation*}$$

where *g_c_* is the grey value of the central pixel and *g_p_* indicates the grey values of the circularly symmetric neighbourhood from *p* = 0 to *P* − 1 and *g_p_* = *x_P,R,p_*. In addition, *P* stands for the number of surrounding pixels in the circular neighbourhood with the spatial resolution of the neighbourhood *R*. Also, *s*(*x*) symbolizes the thresholding function, which helps the LBP algorithm to gain illumination invariance against any monotonic transformation. The probability distribution of the 2^*p*^ LBP patterns represents the characteristic of the texture image. The mentioned parameters of the LBP algorithm control how patterns are computed for each pixel in input images.

Rotating an image causes diverse LBP codes. Therefore, LBP codes need to rotate back to the position of the reference pixel in order to invalidate the results of translating a pixel location and generate multiple identical versions of binary codes. To address the problem of the image rotation effect, a rotation-invariant LBP has been defined as follows [14, 56]:

(6) $$\begin{equation*}
{\rm{LBP}}_{P,R}^{{\rm{ri}}} = {\rm{min}}\left\{ {{\rm{ROR}}\left( {{\rm{LB}}{{\rm{P}}_{P,R}},i} \right){\rm{\ }}|i = 0,{\rm{\ }}1, \ldots ,P - 1} \right\},
\end{equation*}$$

where the function ${\rm{ROR}}( {x,i} )$ performs an *i*-step circular bit-wise right shift on the *P*-bit number *x*. The rotation-invariant LBP is formed by circularly rotating the basic LBP code and keeping the rotationally unique patterns that result in a significant reduction in feature dimensionality.

For uniform patterns, ${\rm{LB}}{{\rm{P}}_{P,R}}$ refers to the number of spatial transitions in the patterns and the ${\rm{\ LBP}}_{P,R}^{u2}$ patterns need to have at most 2 bitwise transitions from 0 to 1 or vice versa. As for a given pattern of *P* bits, the uniform descriptor produces *P*(*P* − 1) + 3 output bins, which consist of *P*(*P* − 1) + 2 bins for distinct uniform patterns, and a single bin $\ ( {P + 1} )$ assigned to all non-uniform patterns. To overcome poor discrimination, due to the crude quantization of angular space at 45° intervals, the rotation-invariant uniform descriptor ${\rm{LBP}}_{P,R}^{{\rm{riu}}2}$, which has a *U* value of ≤2, is defined as follows [14]:

(7) $$\begin{equation*}
{\rm{LBP}}_{P,R}^{{\rm{riu}}2} = \left\{ {\begin{array}{@{}*{1}{c}@{}} {\mathop \sum \nolimits_{p = 0}^{P - 1} s\left( {{g_p} - {g_c}} \right),{\rm{\ if\ }}U\left( {{\rm{LB}}{{\rm{P}}_{P,R}}} \right) \le 2}\\ {P + 1,\ \quad \quad \quad \quad {\rm{if\ }}U\left( {{\rm{LB}}{{\rm{P}}_{P,R}}} \right) > 2} \end{array}} \right.
\end{equation*}$$

The other patterns are labelled “miscellaneous” and grouped into a single value. To map from ${\rm{LB}}{{\rm{P}}_{P,R}}$ to ${\rm{\ LBP}}_{P,R}^{{\rm{riu}}2}$, the rotation invariant uniform descriptor has *(P* + 2) distinct output patterns. Correspondingly, the ${\rm{\ LBP}}_{8,1}^{{\rm{riu}}2},{\rm{\ LBP}}_{16,2}^{{\rm{riu}}2}$, and ${\rm{\ LBP}}_{24,3}^{{\rm{riu}}2}$ operators have 10, 18, and 26 bins, respectively.

## Support Vector Machines

After the dominant features are extracted using the LBP method, the next stage is classification. There are several different classification methods, including decision trees, SVM, neural networks, *k*-nearest neighbour method, and the Bayesian classifier. One of the efficient classification methods is SVM, due to its high performance in many applications, such as face recognition [57, 58], weed identification [59, 60], and disease detection in plant leaves [61, 62]. Therefore, the optimal combination of the LBP descriptors and the SVM classifier can lead to high plant discrimination accuracy. Furthermore, the SVM method has become widespread for classifying objects. It is also regarded as an effective and robust supervised classifier owing to its capability of dealing with pattern recognition problems in image processing and preventing over-fitting and noise data [63, 64]. SVM was originally introduced in 1992 [65] and then significantly extended by many other researchers. A binary classification SVM was first proposed [66]. Given a training dataset of images (*x_i_, y_i_*), where ${x_i} \in {\mathbb{R}^d}$ for i = 1, 2, 3… *N* (images) with a label ${y_i} \in \{ { - 1,\ 1} \}$, the SVM binary classifier $f( x )$ predicts a label *y* as follows [66]:

(8) $$\begin{equation*}
f\left( {{x_i}} \right)\left\{ {\begin{array}{@{}*{1}{c}@{}} { \ge 0\ \ {y_i} = \ + 1\ }\\ { < 0\ \ {y_i} = \ - 1} \end{array}} \right.
\end{equation*}$$

For example, ${y_i}\ f( {{x_i}} )\ > \ 0$ is considered as a correct classification. The optimization problem solved for binary classification is formulated as follows [65, 67]:

(9) $$\begin{equation*}
{\rm{mi}}{{\rm{n}}_{w,b,{\rm{\xi }}}} = \frac{1}{2}{w^T}w + C\mathop \sum \nolimits_{i = {\rm{\ }}1}^{\rm{l}} {{\rm{\xi }}_i}
\end{equation*}$$

subject to the constraint ${y_i}( {{w^T}\phi ( {{x_i}} ) + b} ) \ge {\rm{\ }}1--{{\rm{\xi }}_i}{\rm{\ with\ }}{{\rm{\xi }}_i}{\rm{\ }} \ge 0,i = {\rm{\ }}1,{\rm{\ }} \ldots ,l$.

According to Equation (9), the training data ${x_i}$ are mapped into a higher dimensional space by the function $\phi $ and every constraint can be satisfied if ${{\rm{\xi }}_i}$ is sufficiently large. In addition, *C* > 0 is the regularization parameter, *w* is known as the weight vector, and *b* is the bias. The SVM method generates an optimal hyperplane with the maximal margin between classes in the higher dimensional space. A kernel function $K( {{x_i},\ {x_j}} )$ is represented as $\phi {( {{x_i}} )^T}\phi ( {{x_j}} )$ and 2 kernels including polynomial and radial basis function (RBF) are applied in this article. The polynomial and RBF kernels with kernel parameters ${\rm{\gamma }},{\rm{\ }}$*r, d* are given by [68]

(10) $$\begin{equation*}
{\rm{Polynomial\ SVM}}:K\left( {{x_i},{x_j}} \right){\rm{\ }} = {\rm{\ }}{\left( {{\rm{\gamma }}{x_i}^T{x_j}{\rm{\ }} + r} \right)^d},{\rm{\ \gamma \ }} > {\rm{\ }}0,
\end{equation*}$$

(11) $$\begin{equation*}
{\rm{RBF\ SVM}}:K\left( {{x_i},{x_j}} \right) = \exp \left( { - {\rm{\gamma }}{{\left\| {{x_i} - {x_j}} \right\|}^2}} \right)\ ,{\rm{\ \gamma \ }} > {\rm{\ }}0.
\end{equation*}$$

Kernel selection has long been a problem. In this article, a study is conducted using independent test sets to compare kernels and select the best one.

## Data Collection

**Figure 1:**
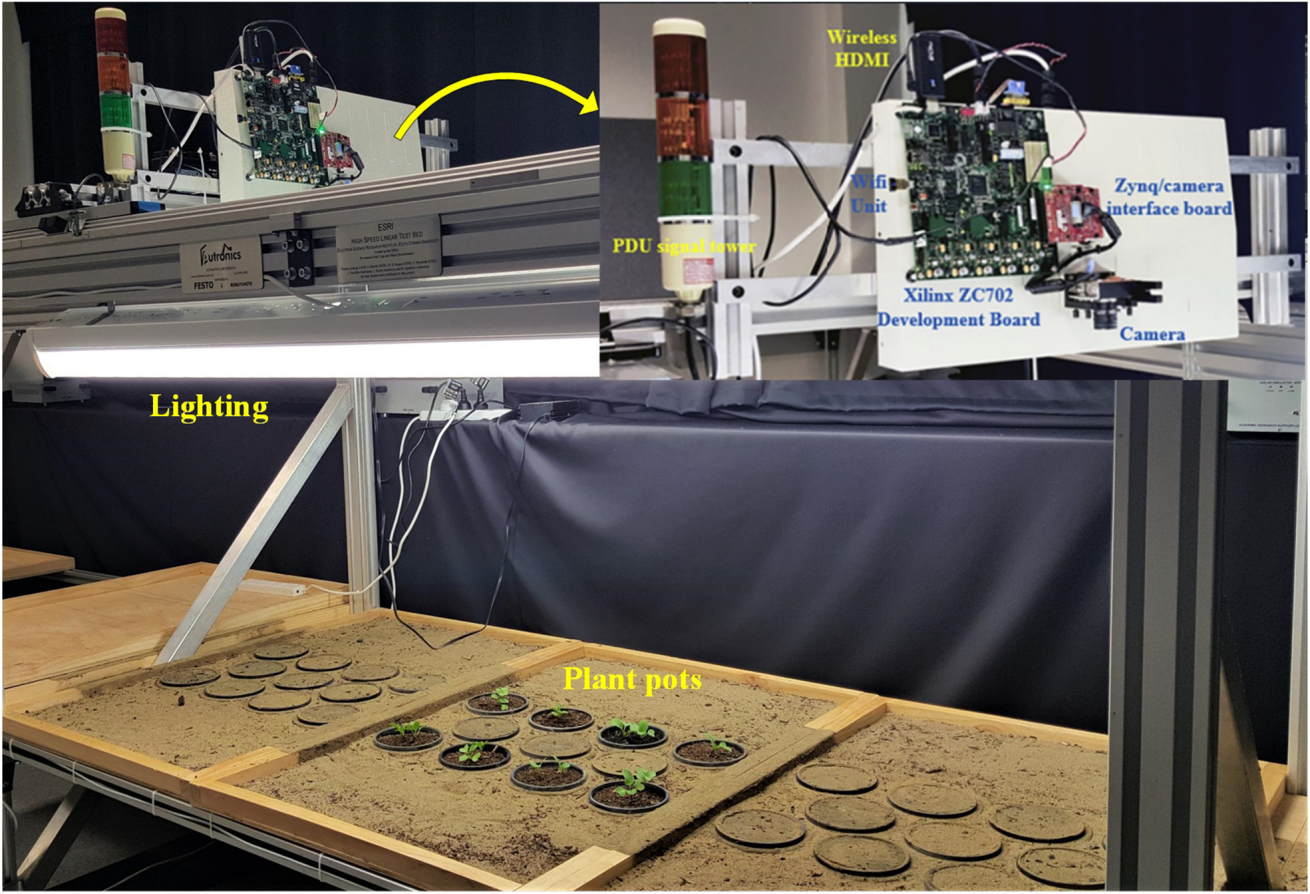
A high-speed testbed system used for controlled data capture [43]. This system has two components including (Plant Discrimination Unit) PDU based on spectral reflectance techniques and a Xilinx Zynq ZC702 development platform.

As mentioned by Le et al. [43], all data were captured on a custom-built testing facility in Fig. 1 at ESRI (Electron Science Research Institute), Edith Cowan University, Australia. Particularly, a Xilinx Zynq ZC702 development platform [65] captured HD images (1,920 **×** 1,080 pixels) at 60 frames per second and used an On-Semi VITA 2000 camera sensor. All images captured by the camera had a spatial resolution of $\approx $1 mm/pixel and size of 228 **×** 228 pixels, which were down-sampled by a factor of 2 from a size of 456 **×** 456 pixels. Moreover, the vertical height of the camera above the surface of the plant pots was 980 mm and the camera focal length was 9 mm.

In this article, we continue to use the bccr-segset dataset to compare the performance of the novel combination of the LBP algorithm and contoured mask with coefficient *k* with that of the combined LBP operators reported by Le et al. [43]. In addition, a new dataset of broadleaf images including only canola and radish leaves is captured to objectively evaluate the detection capability of the proposed approach.

## Methods

**Figure 2:**
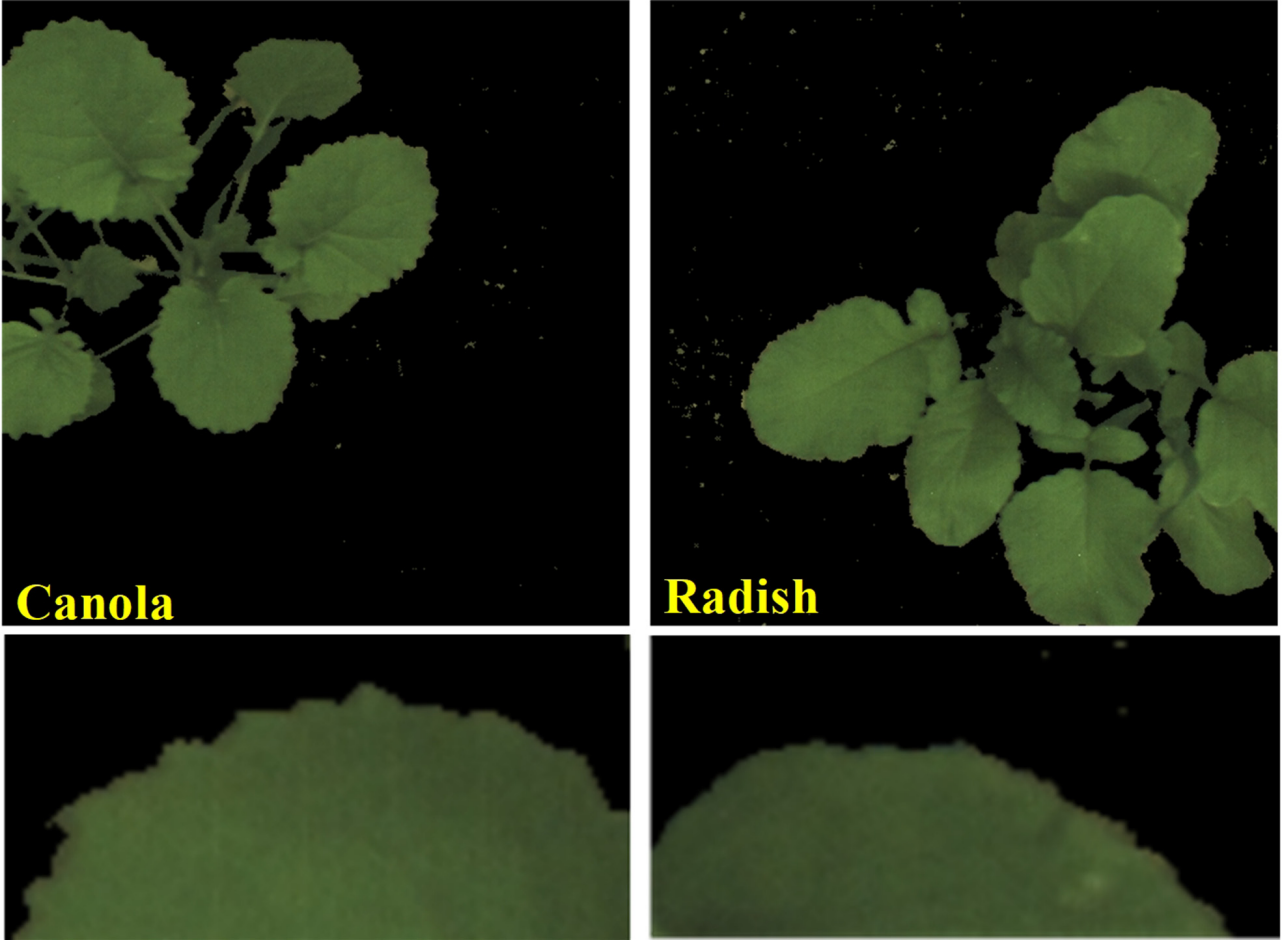
Full and zoomed-in images of canola and radish leaves in the third stage.

In the previous article [43], 3 different LBP operators $\mathrm{LB}\mathrm{P}_{8,1}^{\mathrm{riu}2},\ \mathrm{LB}\mathrm{P}_{16,2}^{\mathrm{riu}2}$, and $\mathrm{LB}\mathrm{P}_{24,3}^{\mathrm{riu}2}$ and the SVM method were combined to detect and classify broadleaf and narrow-leaf plants. The results confirmed that the classification accuracies between broad and narrow leaves were higher than those between broadleaf groups. The recognition of leaves is based on the observation of their morphological features such as texture and shape. According to our bccr-segset dataset, canola and radish plants belong to the broadleaf group, develop as a rosette, and have lobes. However, there are some differences between leaf shapes on the canola and radish plants. When the edge of each leaf is observed closely at the third stage in Fig. 2, canola leaves have outward-pointing teeth and radish leaves have a rounded shape with curved-toothed edge. In other words, from the glossary of leaf morphology, the leaf margin of canola is sinuate while the edge of radish is undulate with a wavy edge, shallower than sinuate [69]. For canola leaves at the fourth growth stage, their lobes are often completely separated towards the base of the leaf. With regard to older radish leaves, they have a larger rounded lobe at the tip of the leaf, some pairs of side lobes, and each set is progressively smaller toward the base.

To overcome the limitation of the combined LBP operators in the previous article, a novel method has been developed for amplifying the dominant features of canola and radish leaves. The flow chart below describes this method in detail.

**Figure 3:**
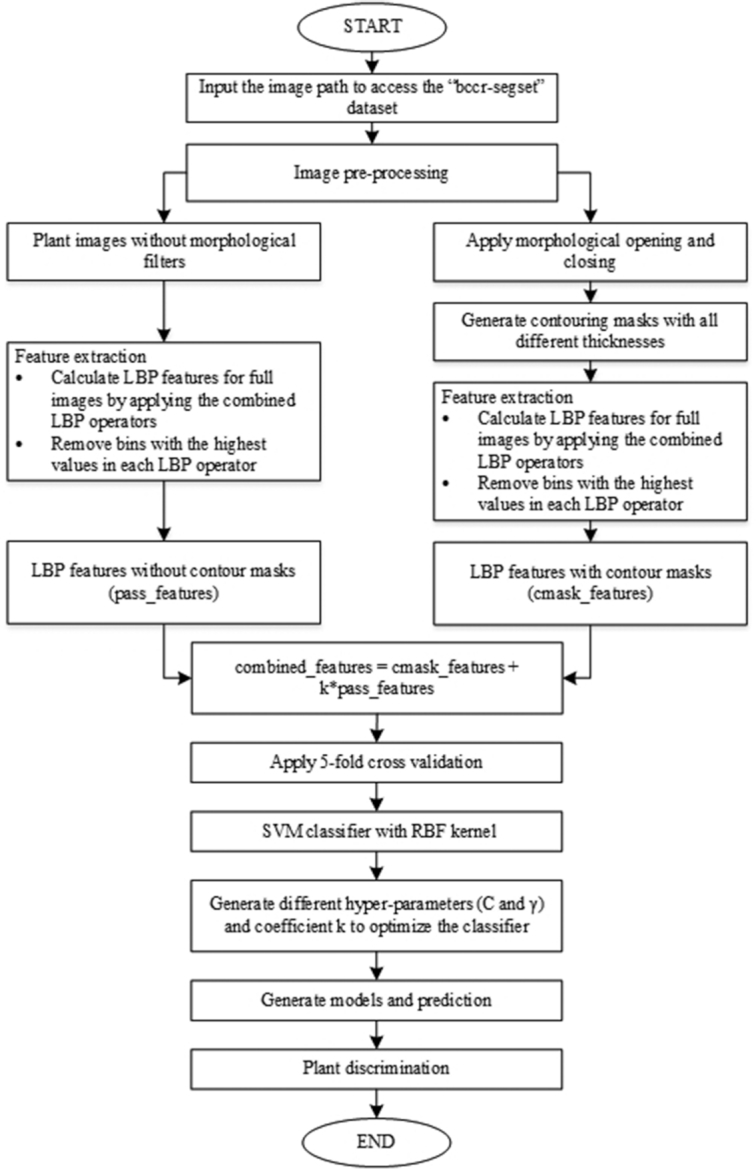
A flow chart describing the procedures of the novel method through steps, namely, filtering LBP bins, extracting features, masking images based on contours, and classifying plant leaves.

To begin with, we input the bccr-segset dataset into the plant classification program. The dataset was processed in 2 branches: (i) the dataset was input to the feature extraction block without applying the morphological operations, and (ii) the dataset applied the morphological opening and closing, and generated contour masks with different thicknesses as shown in Fig. 3. To be more specific in the second branch, a 5 × 5 morphological filter was created to implement the morphological opening and closing on all plant images in the dataset. By selecting a threshold, grey-scale images were converted into binary images to get better accuracy. Here, we masked all plant images with contours, i.e., boundaries around selected plant images. The findContours function and drawContours function in OpenCV were used, and then all the masks of plant images of different thicknesses were stored. This eliminates the need to recalculate when the thickness was changed.

The next stage of both branches was going through the feature extraction block. Particularly, LBP features were computed for full images in the mentioned dataset by incorporating ${\rm{LBP}}_{8,1}^{{\rm{riu}}2} + {\rm{LBP}}_{16,2}^{{\rm{riu}}2} + {\rm{LBP}}_{24,3}^{{\rm{riu}}2}$ operators, which are accumulated into a histogram of *P* + 2 bins (with *P* = 8, 16, 24 corresponding to each LBP operator). Each bin denotes an estimate of the probability of encountering the corresponding pattern in the plant image. The discrete histograms of the ${\rm{LBP}}_{P,R}^{{\rm{riu}}2}$ operators were calculated over plant images. Note that it is not necessary for all bins in the LBP histogram to contain useful information for plant leaf detection. It is observed that for the LBP histograms of plant images at the bin level, the ninth bin of ${\rm{\ LBP}}_{8,1}^{{\rm{riu}}2}$, the 17th bin of ${\rm{LBP}}_{16,2}^{{\rm{riu}}2}$, and the 25th bin of ${\rm{LBP}}_{24,3}^{{\rm{riu}}2}$ contain a much higher number of hits than the remaining bins from the LBP histogram. A further investigation shows that the LBP values for these bins correspond to patterns that have no pixel variations. For example, all pixels are constant values such as the values of background pixels. However, the remaining bins correspond to LBP patterns that mainly capture the intensity variations of green pixels (plant leaves). Therefore, bins *P* + 1 (the ninth bin of ${\rm{\ LBP}}_{8,1}^{{\rm{riu}}2}$, the 17th bin of ${\rm{LBP}}_{16,2}^{{\rm{riu}}2}$, and the 25th bin of $\ {\rm{LBP}}_{24,3}^{{\rm{riu}}2}$) were removed from each LBP histogram in order to better scale the remaining bins. According to the combination of 3 different spatial resolutions and different angular resolutions in LBP operators, 3 bins including ninth, 27th, and 53rd were removed in the joint histogram of the ${\rm{LBP}}_{8,1}^{{\rm{riu}}2} + {\rm{LBP}}_{16,2}^{{\rm{riu}}2} + {\rm{LBP}}_{24,3}^{{\rm{riu}}2}$ operator (10 bins + 18 bins + 26 bins = 54 bins). After applying the ${\rm{LBP}}_{8,1}^{{\rm{riu}}2} + {\rm{LBP}}_{16,2}^{{\rm{riu}}2} + {\rm{LBP}}_{24,3}^{{\rm{riu}}2}$ operator for the plant images, the resultant images were called as LBP images.

**Figure 4:**
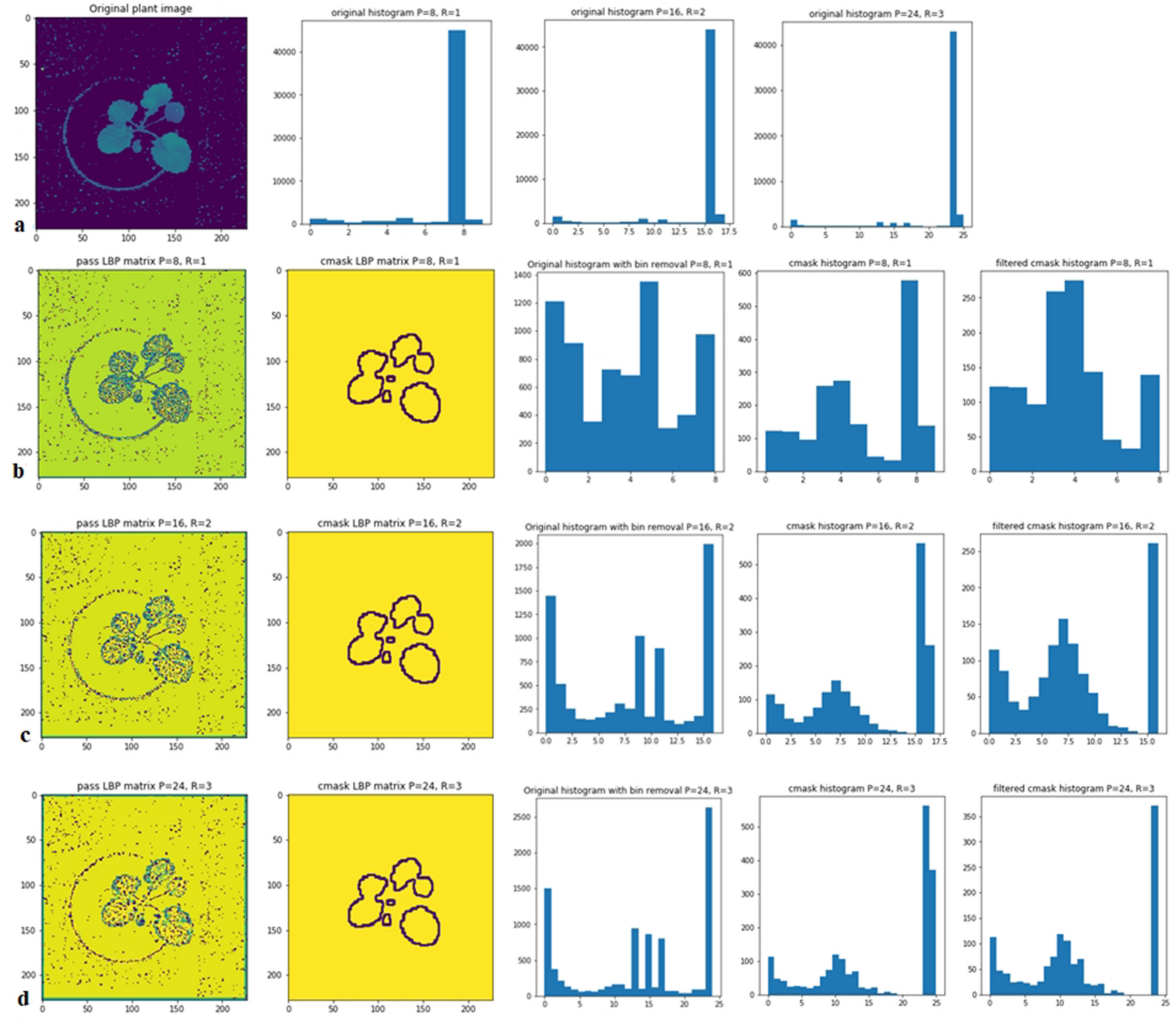
(a) An original canola leaf image and its LBP histograms corresponding to the ${\mathrm{LBP}}_{8,1}^{{\mathrm{riu}}2},{\boldsymbol{\ }}{\mathrm{LBP}}_{16,2}^{{\mathrm{riu}}2}$, and ${\mathrm{LBP}}_{24,\ 3}^{{\mathrm{riu}}2}$ operators. (b–d) LBP images, LBP images with contour masks, and their original LBP histograms and filtered LBP histograms are presented by implementing ${\mathrm{LBP}}_{8,1}^{{\mathrm{riu}}2},{\boldsymbol{\ }}{\mathrm{LBP}}_{16,2}^{{\mathrm{riu}}2}$, and ${\mathrm{LBP}}_{24,3}^{{\mathrm{riu}}2}$ operators, respectively. Multiresolution analysis can be achieved by altering *P* and *R* of LBP operators and then combining these operators as shown in Fig.   5.

Fig. 4 illustrates an example of the process shown in the flow chart (Fig. 3). In Fig. 4a we show an original canola leaf image and its 3 histograms corresponding to the ${\rm{LBP}}_{8,1}^{{\rm{riu}}2},\ {\rm{LBP}}_{16,2}^{{\rm{riu}}2}$, and ${\rm{LBP}}_{24,3}^{{\rm{riu}}2}$ operators. The ninth, 17th, and 25th bins in each operator have the highest level of the distribution of patterns. The LBP-based canola leaf image and contour mask, the original histogram, and the filtered histogram of the contour masks are shown in Fig. 4b–d with the ${\rm{LBP}}_{8,1}^{{\rm{riu}}2},\ {\rm{LBP}}_{16,2}^{{\rm{riu}}2}\ $, and ${\rm{LBP}}_{24,3}^{{\rm{riu}}2}$ operators, respectively. It is apparent that the feature distribution is easily observed in the other bins of the LBP histogram with bin removal. Interestingly, dominant features such as edge and corner patterns in other bins can be seen clearly by removing some specific bins (ninth, 17th, and 25th bins) in the LBP histograms. Similarly, plant features in the histogram of the LBP-based contour mask with bin removal also present their significance. It is noted that the bin number of the LBP histogram in Fig. 4, calculated in a Python code, has an index range from 0 to [(*P* + 2) − 1] bins. Note that the bin number mentioned in this article ranges from 1 to *P* + 2. For example, the ${\rm{LBP}}_{8,1}^{{\rm{riu}}2}$ operator has an index range from 0 to 9 but bin number from 1 to 10.

As shown in Fig. 3, the filtered LBP features without contour mask in plant images are denoted as "pass_features." The method used to generate images is referred to as the filtered LBP method (FLBP). The FLBP method is applied to the plant images, and results in 51 features are calculated over the entire image. The FLBP-based contour masks are denoted as "cmask_features." The method used to create images consisting of cmask_features is referred to as the filtered LBP–based contour mask (FLBPbCM). Applying the FLBPbCM method to the plant images also results in 51 features computed only on the contours. The remaining region in the image is set to the maximum value (255) in the LBP matrix and ignored when generating the LBP histogram.

**Figure 5:**
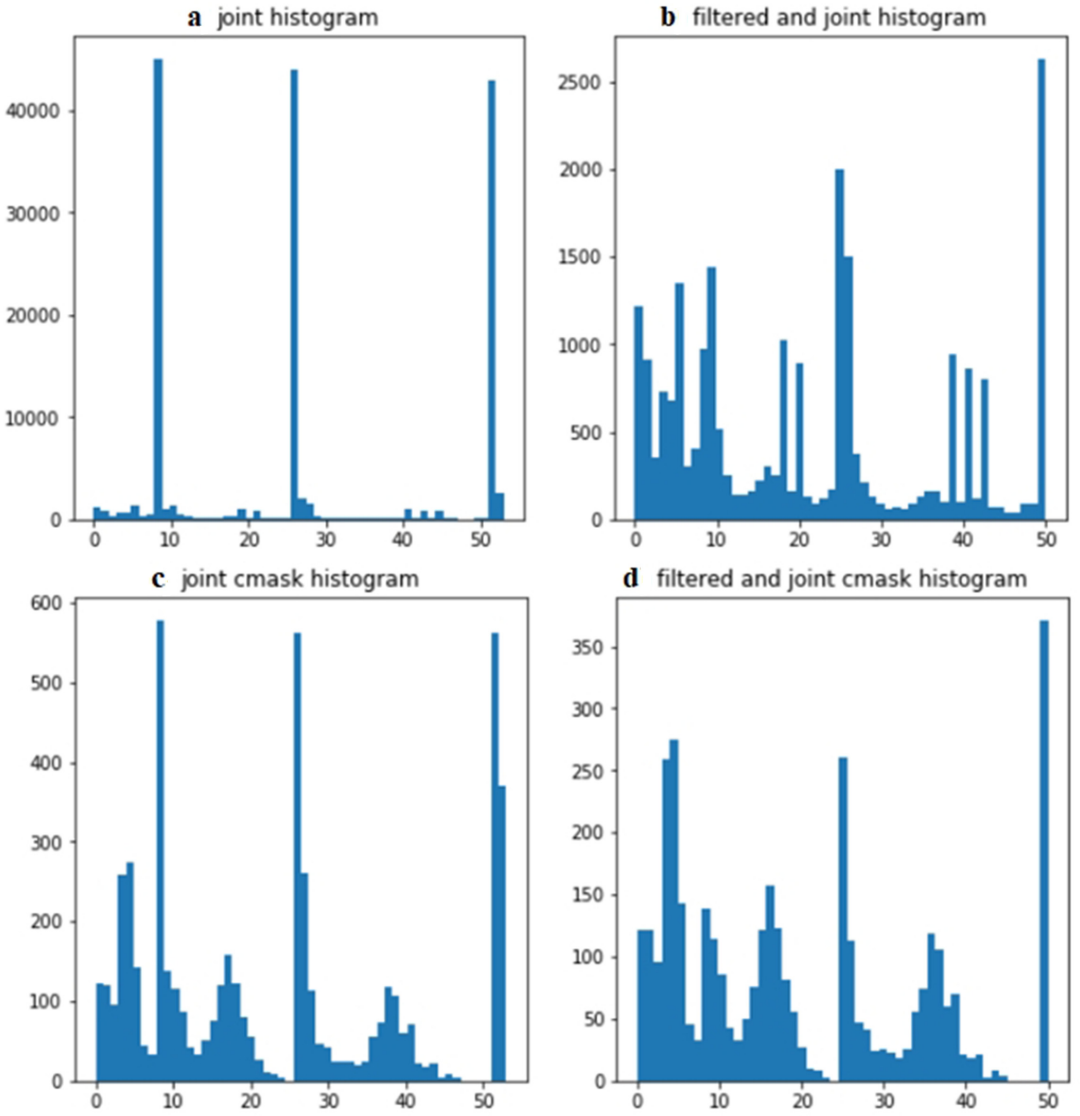
Four different LBP histograms of a canola leaf image. (a) combining 3 operators (${\mathrm{LBP}}_{8,1}^{{\mathrm{riu}}2},{\boldsymbol{\ }}{\mathrm{LBP}}_{16,2}^{{\mathrm{riu}}2}$, and ${\mathrm{LBP}}_{24,3}^{{\mathrm{riu}}2}$). (b) A filtered and joint histogram is generated by eliminating the ninth, 27th, and 53rd bins in the joint histogram. (c) A joint cmask histogram is generated by applying the LBP method with a contour mask. (d) Removing the ninth, 27th, and 53rd bins in the joint cmask histogram.

The novelty of the present k-FLBPCM is a combination of pass_features and cmask_features. Owing to the high bin values in the FLBP method as shown in Figs 4 and 5, cmask_features are scaled by multiplying pass_features by coefficient *k* in the k-FLBPCM method. For example, Table 1 shows the distributions of patterns (bin values) in a typical canola image. It demonstrates that by combining the pass_features (in the FLBP method) and cmask_features (in the FBLPbCM method), the bin values of the k-FLBPCM method have better balance between these 2 feature sets. The purpose of multiplying coefficient *k* (*k* ≤ 1) by pass_features is to reduce the gap between the bin values of the cmask_features and pass_features.

**Table 1:** The bin values of a typical canola image using FLBP, LFBPbCM, and the combined k-FLBPCM methods

Bin values of different methods	Bin 1	Bin 2	Bin 3	Bin 4	Bin 5	Bin 6	Bin 7	Bin 8	Bin 10
FLBP	1212	913	355	727	680	1351	305	402	974
FLBPbCM	122	121	96	259	275	143	45	33	139
k-FLBPCM with *k* = 0.5	728	577.5	273.5	622.5	615	818.5	197.5	234	626

After the feature extraction step, the plant images are classified by using SVM kernels. Initially, 5-fold cross-validation was used to divide the dataset into 5 subsets. Owing to the different plant growth stages in the dataset, images at each growth stage are equally divided in each subset as well. A single subset of the dataset is used for testing while the remaining 4 subsets of the dataset are used for training. The cross-validation process was iteratively applied 5 times, with the test subset changed each time. This procedure helps to prevent overfitting. After generating the training model by selecting the RBF kernel in SVM and making predictions, the classification accuracies of the methods were calculated by using performance metrics such as accuracy, precision, recall, and F1-score.

## Results

The results are divided into 2 sections. The first section presents the average classification accuracies of the broadleaf classes consisting of canola and radish. The effectiveness of the proposed k-FLBPCM method is evaluated on the basis of factors including feature extraction (by comparing among the FLBP, FLBPbCM, and k-FLBPCM methods), different SVM kernels (the second-order polynomial kernel and RBF kernel), contour thickness, and LBP parameters *P* (the total number of the neighbouring pixels) and *R* (the radius), as well as the coefficient *k*. In the second section, the parameters (*C*, γ, coefficient *k*, and thickness) for the classification of all 4 classes in the bccr-segset dataset including canola, corn, radish, and background are optimized to obtain improved classification accuracy. The computer used in these experiments had a 3.4 GHz processor and 16 GB RAM and ran Python 2.7.13.

### Results of the k-FLBPCM, FLBPbCM, and FLBP methods in classifying 2 different broadleaf plants

Canola and radish images were taken from the bccr-segset dataset. The train and test sets of canola and radish classes consist of 15,000 images (7,500 images in each class). After applying the FLBP, FLBPbCM, or k-FLBPCM method, SVM was used to classify the 2 broadleaf classes including canola and radish plants. The classification accuracies of the second-order polynomial kernel and the RBF kernel were compared. In this experiment, *C* = 10, 60, γ = ${10^{ - 5}}\ ,\ {10^{ - 6}}$, and thickness = 2 were selected. The values of *C* and γ selected were typical values, before any optimization had been performed.

The results of using 2 SVM kernels (the second-order polynomial and RBF kernels) on the given dataset for classification are summarized in Table 2. In particular, the average classification accuracy of the k-FLBPCM method (*C* = 10, γ = ${10^{ - 5}}\ $,*k* = 0.5, and 0.2) with the RBF kernel was 97.32%, followed by 96.40% corresponding to the k-FLBPCM method with coefficient *k* = 0.1. Meanwhile, the average classification accuracy of the k-FLBPCM method (*C* = 10, γ = ${10^{ - 5}}\ $, *k* = 0.5) with the second-order polynomial kernel was just 95.46%. Similarly, the case (*C* = 60, γ = ${10^{ - 6}}\ $) of the k-FLBPCM method with the RBF kernel was also higher than the polynomial kernel of degree 2. In addition, the FLBP method with the RBF kernel had a higher classification rate than the polynomial kernel. As for the FLBPbCM method (*C* = 10, γ = ${10^{ - 5}}\ $), the RBF kernel had a classification accuracy of 94.07% in comparison to the second-order polynomial kernel at 88.53%. These results show the RBF kernel, which nonlinearly maps features into a higher dimensional space, resulting in higher classification accuracy for all 3 methods (FLBP, FLBPbCM, and k-FLBPCM).

**Table 2:** The average classification accuracy score of the k-FLBPCM, FLBPbCM, and FLBP methods with the second-order polynomial and RBF kernels

*C*	γ	Thickness	Method	Accuracy score	
				Polynomial kernel of degree 2 (%)	RBF kernel (%)
10	1E−05	2	k-FLBPCM method, *k* = 0.5	95.46	97.32
10	1E−05	2	k-FLBPCM method, *k* = 0.2	94.91	97.32
10	1E−05	2	k-FLBPCM method, *k* = 0.1	94.27	96.40
60	1E−06	2	k-FLBPCM method, *k* = 1	94.92	97.50
60	1E−06	2	k-FLBPCM method, *k* = 0.5	94.56	96.89
60	1E−06	2	k-FLBPCM method, *k* = 0.2	93.55	96.06
10	1E−05	No thickness	FLBP method	93.53	95.36
60	1E−06	No thickness	FLBP method	93.74	96.72
10	1E−05	2	FLBPCM method	88.53	94.07
60	1E−06	2	FLBPCM method	88.26	94.83

A second experiment was conducted to investigate the effects of the hyper-parameters *C* and γ, as well as the coefficient *k*, on the classification accuracy of canola and radish images. Various pairs of (*C*, γ) values were tried and good results were obtained with exponentially growing sequences of *C* and γ [70]. Therefore, we chose the ranges of *C*, γ and coefficient *k* as follows: *C* = 1, 10, 30, 60, 100, 1,000, γ = $\ {10^{ - 4}},\ {10^{ - 5}}\ ,\ {10^{ - 6}},\ {10^{ - 7}}$. In addition, as mentioned in the Methods section, we selected *k* (*k*≤ 1) randomly from 0.1 to 1 (*k* = 0.1, 0.2, 0.5, 0.7, 0.8, and 1.0). We tested all these values in the experiments to observe the variation of values and chose an optimal set *k, C*, and γ when these parameters reach the highest classification accuracy. As reported in Table 3, the k-FLBPCM method had the highest classification accuracy, averaged over the 5-fold cross- validation, in the first pair (*C* = 30, γ = ${10^{ - 5}}\ $, thickness = 2, *k* = 0.2) and the second pair (*C* = 60, γ = ${10^{ - 6}}\ $, thickness = 2, *k* = 1), at 97.50%. In addition, the average classification accuracies of the k-FLBPCM method with different parameters were sorted from high to low. Owing to the large number of combinations possible, only the top 10 cases are listed in Table 3. Owing to the low accuracy of using γ = ${10^{ - 4}}\ $, the parameter γ should be <${10^{ - 5}}$ to improve the classification accuracy of the k-FLBPCM method.

**Table 3:** The average accuracy scores of the k-FLBPCM method with the RBF kernel, varying *C*, γ, and the coefficient *k*

*C*	γ	Thickness	k-FLBPCM method	Accuracy score (%)
30	1E−05	2	*k* = 0.2	97.50
60	1E−06	2	*k* = 1	97.50
60	1E−05	2	*k* = 0.2	97.49
100	1E−05	2	*k* = 0.2	97.45
100	1E−06	2	*k* = 1	97.42
30	1E−06	2	*k* = 1	97.42
100	1E−06	2	*k* = 0.7	97.40
30	1E−05	2	*k* = 0.5	97.37
100	1E−06	2	*k* = 0.8	97.35
60	1E−06	2	*k* = 0.8	97.34

**Figure 6:**
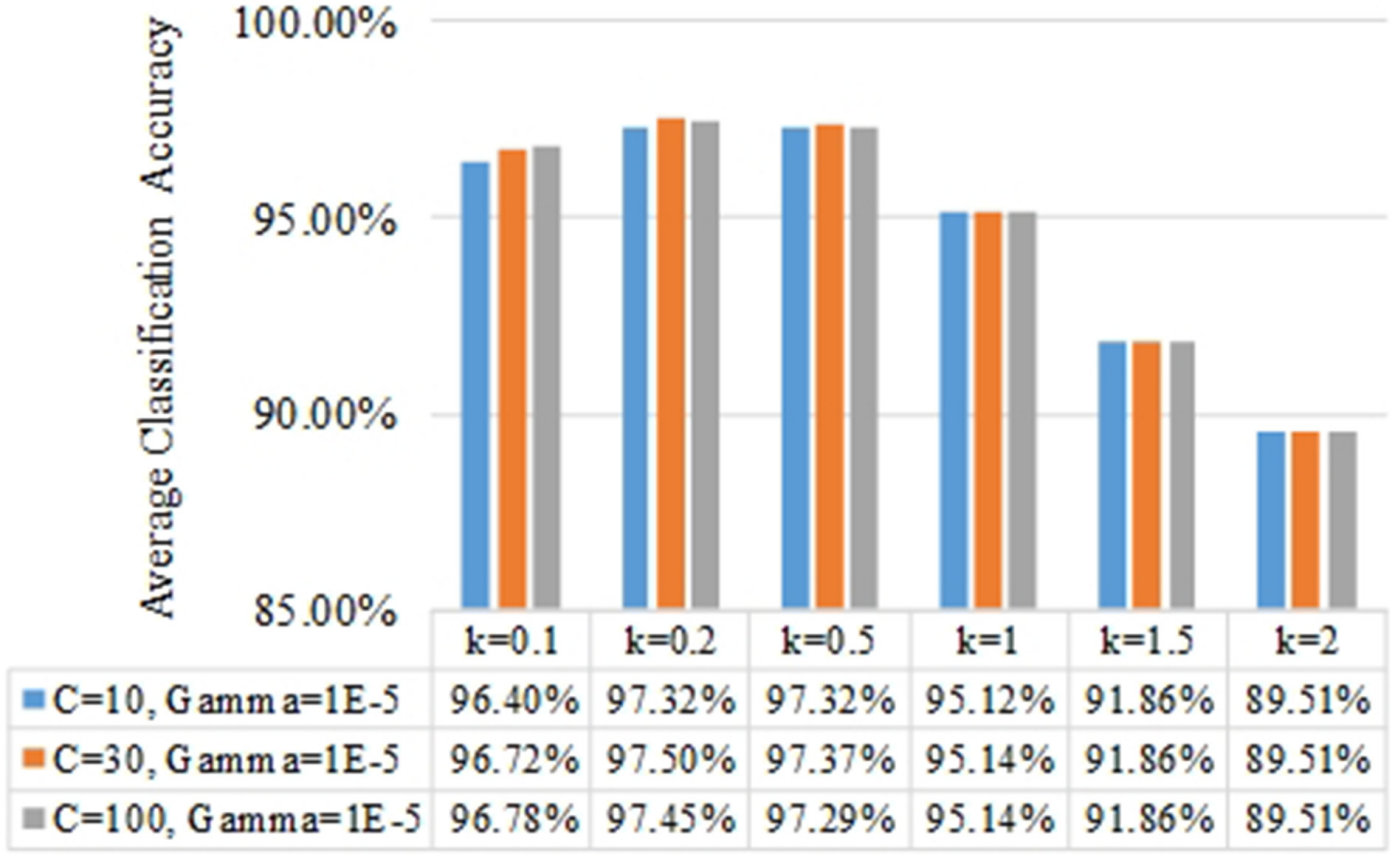
The average classification accuracies of the k-FLBPCM method with different coefficients *k*.

Although all experiments were conducted with different coefficients *k*, this parameter should be ≤1. We find that k ≤ 1 results in optimal accuracy. As shown in Fig. 6, the average classification accuracies of the proposed k-FLBPCM method with *k* ≤ 1 were higher than those with *k* > 1.

#### Comparing the FLBP, FLBPbCM, and k-FLBPCM methods

To check the effectiveness of the k-FLBPCM method in a different dataset, a new set of canola and radish images in 4 different growth stages was collected and designated “can-rad” dataset (published online). A total of 19,600 broadleaf images (9,800 images in each class) were collected at 4 different growth stages. The parameters *C* = 10, 30, 60, 100, 1000, γ = ${10^{ - 5}}\ ,\ {10^{ - 6}}$, and thicknesses from 1 to 8 were selected. Note that the SVM classifier was used only with the RBF kernel in the remaining parts of the experiments. Furthermore, only the 10 highest classification accuracies for each method are listed in Tables 3–5 and the average classification accuracy scores are sorted from high to low.

As can be seen from Tables 4 and 5, the classification accuracy of the FLBP method was 95.13% with *C* = 100 and γ = ${10^{ - 6}}\ $, while that of the FLBPbCM method was 93.95%, lower than the FLBP method. However, when combining the FLBP and FLBPbCM methods (in k-FLBPCM method), the classification accuracy was significantly higher. Table 6 shows that the highest average classification accuracy of the k-FLBPCM method was 96.21%.

**Table 4:** Classification accuracy of the FLBP method

*C*	γ	Classification accuracy of the FLBP method (%)
**100**	1E−06	95.13
1,000	1E−06	95.03
60	1E−06	94.96
30	1E−06	94.92
10	1E−06	94.31
1,000	1E−07	93.92
10	1E−05	93.78
30	1E−05	93.67
60	1E−05	93.62
100	1E−05	93.61

**Table 5:** Classification accuracy of the FLBPbCM method

*C*	γ	Thickness	Classification accuracy of the FLBPbCM method (%)
**30**	1E−05	8	93.95
**30**	1E−05	7	93.95
100	1E−05	2	93.94
30	1E−05	6	93.88
30	1E−05	5	93.88
10	1E−05	8	93.88
10	1E−05	7	93.88
1,000	1E−05	2	93.87
60	1E−05	6	93.87
100	1E−05	6	93.81

**Table 6:** Classification accuracy of the k-FLBPCM method

*C*	γ	Thickness	k-FLBPCM method	Classification Accuracy (%)
**1,000**	**1E−06**	**2**	***k* = 0.5**	**96.21**
30	1E−05	2	*k* = 0.5	96.19
10	1E−05	2	*k* = 0.5	96.18
30	1E−05	4	*k* = 0.5	96.16
30	1E−05	3	*k* = 0.5	96.16
60	1E−05	2	*k* = 0.5	96.15
10	1E−05	4	*k* = 0.5	96.14
10	1E−05	3	*k* = 0.5	96.14
30	1E−05	2	*k* = 0.2	96.13
30	1E−05	4	*k* = 0.2	96.11

**Table 7:** Comparison of the average classification accuracies of the FLBP, FLBPCM, and k-FLBPCM methods

C	γ	Thickness	Method	Accuracy score (%)
30	1E−05	2	k-FLBPCM, *k* = 0.2	98.63
60	1E−05	2	k-FLBPCM, *k* = 0.2	98.61
100	1E−06	2	k-FLBPCM, *k* = 0.8	98.61
30	1E−05	No thickness	FLBP	97.23
60	1E−05	No thickness	FLBP	97.22
100	1E−06	No thickness	FLBP	98.17
30	1E−05	2	FLBPCM	97.04
60	1E−05	2	FLBPCM	97.14
100	1E−06	2	FLBPCM	96.01

#### Effects of the contour thickness on the classification accuracy

**Figure 7:**
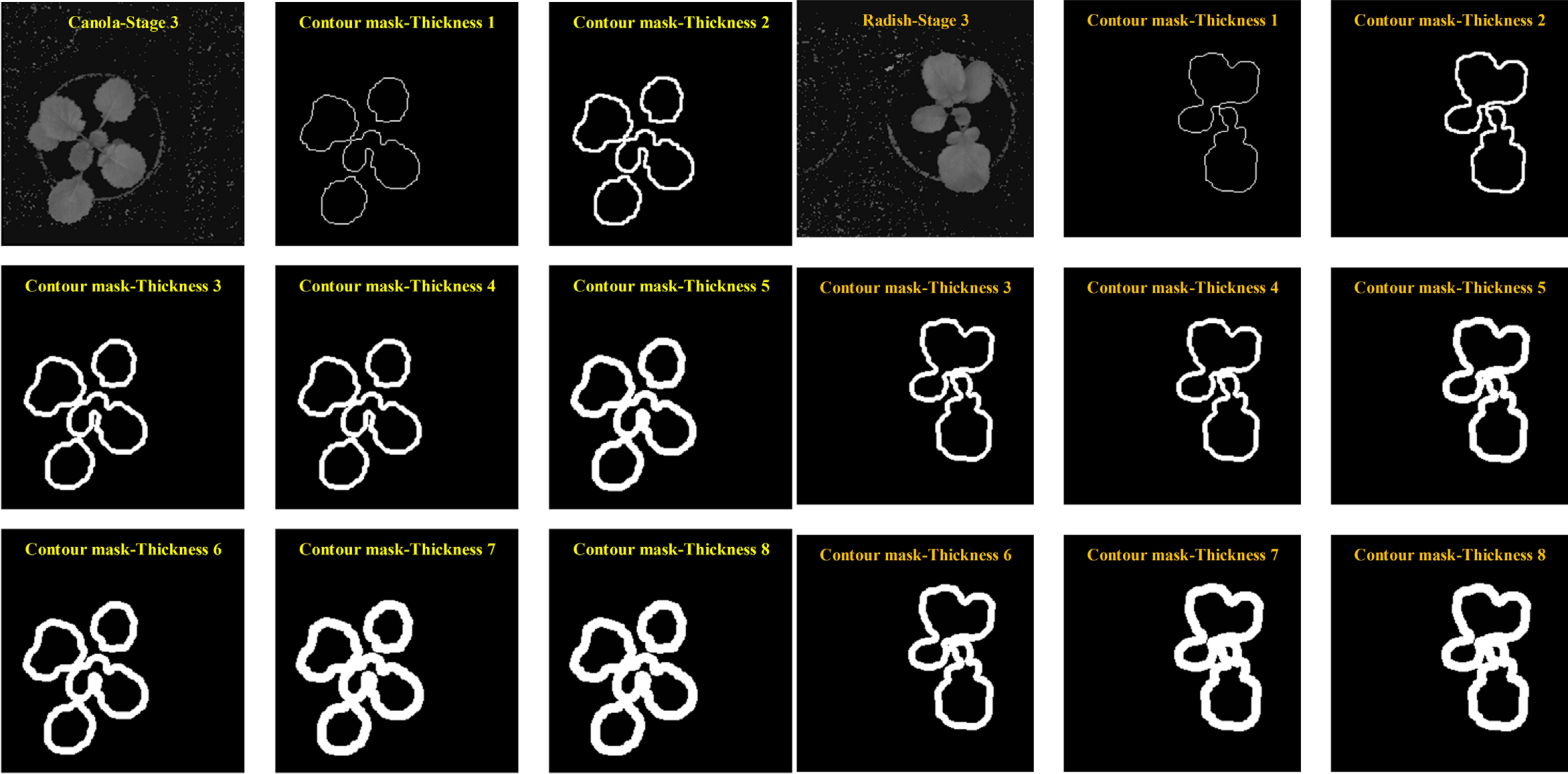
Canola and radish at the third stage with varying thicknesses of the contour lines.

Next, we evaluated the average classification accuracy of the k-FLBPCM method for varying the thicknesses of the contour lines. The can-rad dataset was used for this investigation. We selected *C* = 10, 30, 100, γ = ${10^{ - 5}}\ $, coefficient *k* = 0.5, and thickness from 1 to 8. As can be seen in Fig. 7, 2 images of canola and radish with varying contour thickness are presented at the third growth stage.

**Figure 8:**
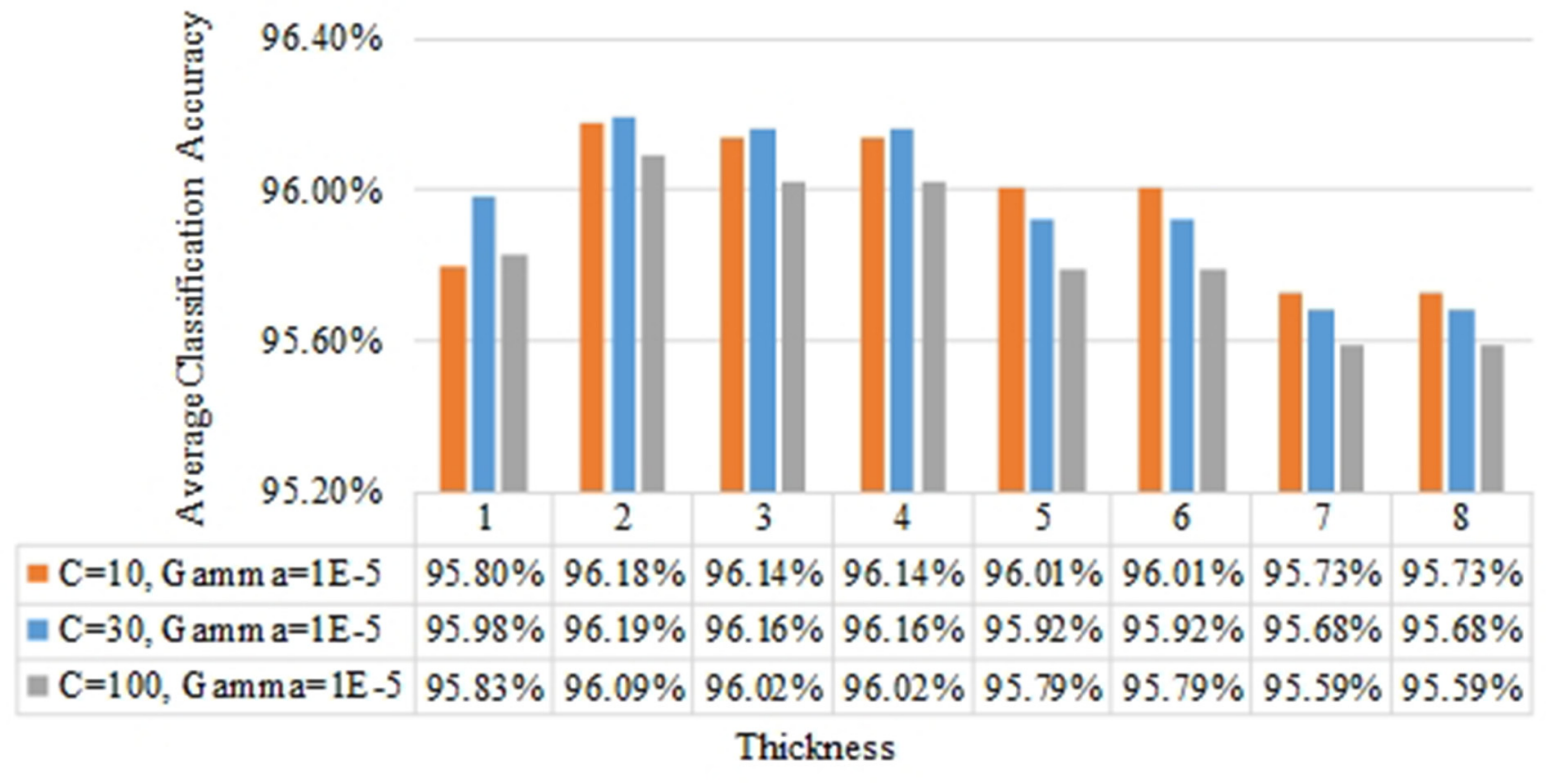
The average classification accuracies of the k-FLBPCM method (coefficient *k* = 0.5) for different contour line thicknesses and 4 growth stages.

The average classification accuracies of the k-FLBPCM method for different thicknesses are reported in Fig. 8. Our proposed k-FLBPCM method attained optimal discrimination between canola and radish at contour thickness of 2 with an accuracy of 96.19% (*C* = 30, γ =${10^{ - 5}}\ $), while the lowest accuracy was 95.73% with thicknesses of 7 and 8. These 2 broadleaf plants displayed morphological similarity at a contour thickness of 2. As shown in Fig. 7, for thickness >2, the leaf features were smoothed by the thick edge, while for a thickness of 1, the edge features were too thin to fully show the difference between the undulate and sinuate patterns.

### Classification capabilities of the k-FLBPCM, FLBPbCM, and FLBP methods

**Figure 9:**
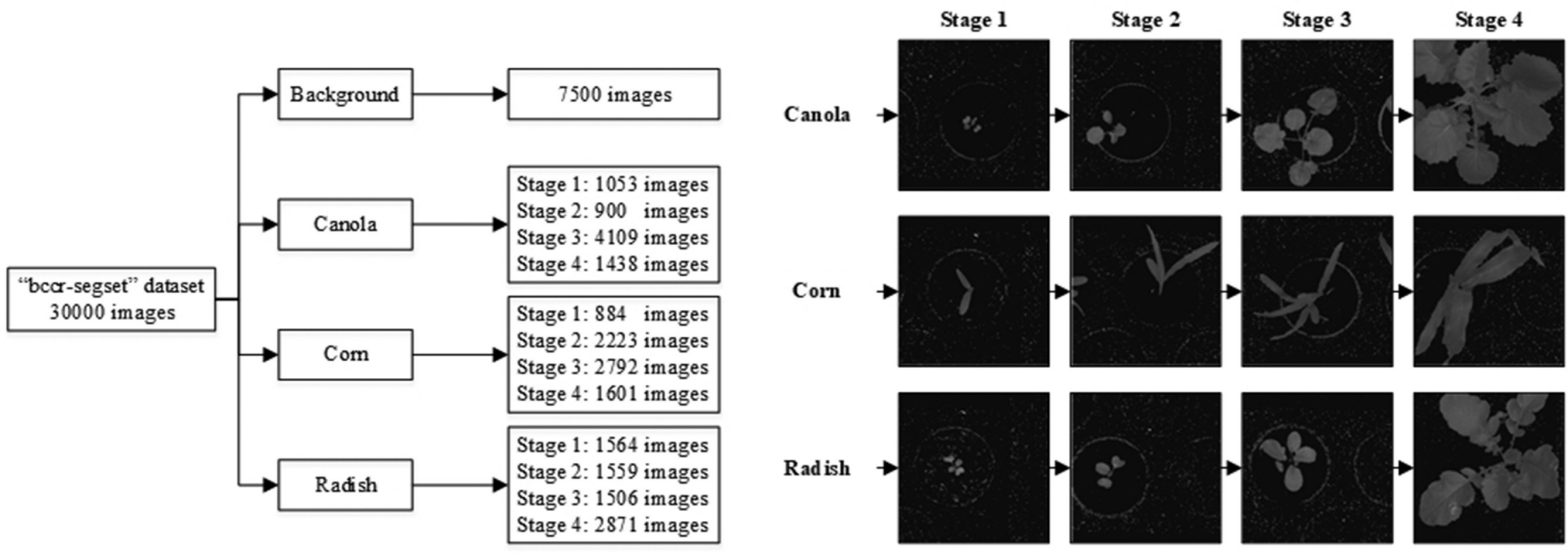
The “bccr-segset” dataset and its 4 growth stages. The average classification accuracies of the FLBP, FLBPCM, and k-FLBPCM methods are listed in Table 7. Note that, in this investigation, the following typical values were selected: *C* = 30, 60, 100 and γ = ${10^{ - 5}}\ $, ${10^{ - 6}}$. The k-FLBPCM method again achieved the highest accuracies among all compared methods, confirming the results in the given “can-rad” dataset.

The k-FLBPCM method was evaluated on the full bccr-segset dataset, which included 30,000 plant images in 4 classes (canola, corn, radish, and background) under different rotations, scales, and illumination conditions. Plant images were taken under different rotation angles (45°, 90°, 135°, 180°, 225°, 270°, 315°, 360°), lighting conditions (sunlight and fluorescent), sizes, and morphologies of plants through 4 growth stages, as illustrated in Fig. 9. The number of plant images at each class and each growth stage is indicated in Fig. 9 [43].

In order to find optimal (*C*, γ) pairs, we investigated the following parameter ranges: *C* = 1, 10, 30, 60, 100, 1,000, γ = $\ {10^{ - 5}},\ {10^{ - 6}}$, *k* = 0.1, 0.2, 0.5, 0.8, 1, and thickness of 2. Only the 10 highest classification accuracies of the k-FLBPCM method are listed in Table 8. This method attained the highest classification accuracy of 98.63% with C = 30, γ = ${10^{ - 5}}\ $, and coefficient *k* = 0.2.

**Table 8:** Average classification accuracies of the k-FLBPCM method for different *C* and γ parameters and coefficients *k*

C	γ	Thickness	k-FLBPCM Method	Accuracy score (%)
30	1E−05	2	*k* = 0.2	98.63
100	1E−06	2	*k* = 0.8	98.61
100	1E−05	2	*k* = 0.2	98.61
60	1E−05	2	*k* = 0.2	98.61
100	1E−06	2	*k* = 1	98.60
60	1E−06	2	*k* = 0.8	98.58
60	1E−06	2	*k* = 1	98.57
1,000	1E−06	2	*k* = 0.5	98.56
30	1E−06	2	*k* = 1	98.56
1,000	1E−06	2	*k* = 1	98.51

The k-FLBPCM method can classify plant images with different conditions, as shown in our 2 datasets, and improve the classification accuracies achieved previously [43]. Particularly, there is a significant improvement in performance when combining LBP features with a contour-based mask. The average classification accuracies of the k-FLBPCM method have increased over the previously described method by up to 6.78% [45].

The F1-score results for each class are indicated in Table 9. Particularly, the F1 scores of the k-FLBPCM method significantly increased to 97.40% and 97.40% for canola and radish, from 84.41% and 83.43%, respectively, which had used combined LBP operators in the previously published article [45]. In addition, the testing time (millisecond/image) of the k-FLBPCM method was faster than that of the combined LBP method [45].

**Table 9:** Comparison of performance metrics between the k-FLBPCM and combined LBP methods for each class

Method	SVM kernel	Classes	Precision (%)	Recall (%)	F1-score (%)	Testing time (ms/image)
k-FLBPCM	RBF kernel	Background	100	100	100	0.491
		Canola	96.80	97.60	97.40	
		Corn	100	100	100	
		Radish	97.60	97.20	97.40	
Combined LBP operators LBP(8,1) + LBP(16,2) + LBP(24,3)	RBF kernel	Background	96.17	98.87	97.50	1.419
		Canola	83.64	85.20	84.41	
		Corn	98.64	96.87	97.75	
		Radish	84.69	82.27	83.46	

**Figure 10:**
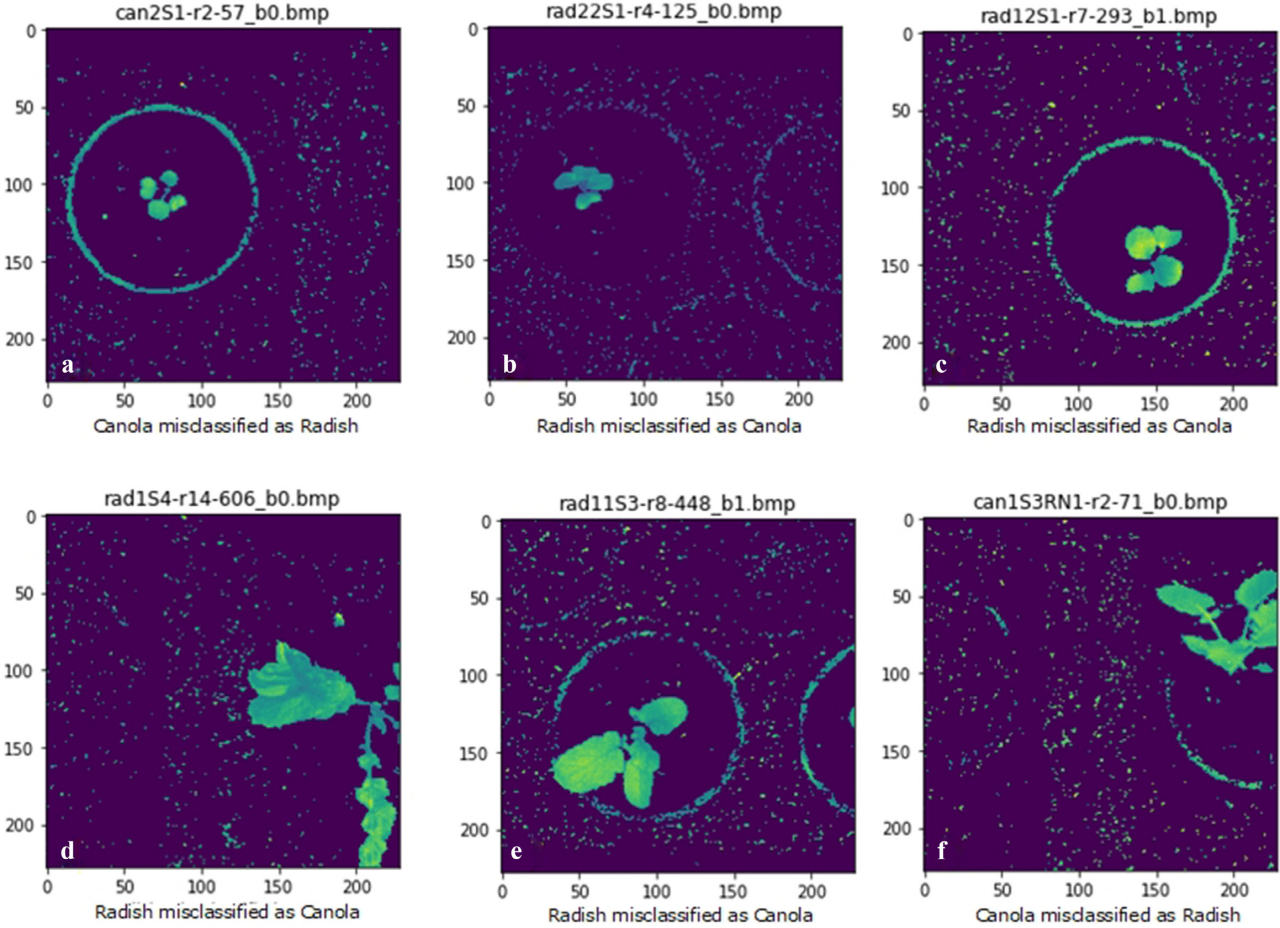
Misclassified images are printed from the model of the k-FLBPCM method with *C* = 30, γ = ${10^{ - 5}}\ $, and *k* = 0.2.

With the aim of reducing the misclassification, we investigated the misclassified images through visual inspection as shown in Fig. 10. The first-stage plants (Fig. 10a–c) appear to have been misclassified owing to the close morphological similarities. In addition, deformity of the leaves and stems, especially arising from perspective distortions (Fig. 10e and f) and leaf diseases (Fig. 10d), can also lead to identification errors. However, the k-FLBPCM method considerably reduced the number of misclassified images and outperformed other methods, obtaining the high classification accuracy at 98.63%.

## Conclusions

In this article, the k-FLBPCM method combining LBP feature extraction with contour masks has been proposed for reducing noise and improving plant classification accuracy. Results have shown that various factors can reduce weed identification accuracy, including outdoor scene complexity and morphological variability of plants. On the basis of the experimental results, the k-FLBPCM method had the best performance of 98.63% accuracy in identifiying morphologically similar plants. This method is particularly useful to discriminate between 2 classes with highly similar morphologies while tolerating morphological variability within each class. Furthermore, results have shown that the execution time of the proposed method is faster than that of the combined LBP method in the previous published article. As a result, the proposed method helps to improve classification of plants with similar morphological features. Furthermore, the fast processing time of this method enhances the ability to implement plant detection in real time.

Future research might consider the potential of the k-FLBPCM method in diverse applications in order to identify objects of similar morphologies. Morphological cell analysis plays an important role in supporting pathologists to accurately detect cancer cells [71, 72]. The advantage of the k-FLBPCM method is that image data can be reused for extracting morphological features and identifying abnormal cells.

## Availability of Supporting Source Code and Requirements

Project name: k-FLBPCM-method

Project home page: https://github.com/vinguyenle/k-FLBPCM-method

Operating system: The code of the k-FLBPCM method was written on Linux.

Programming language: Python 2.7.13

License: GNU General Public License v3.0

RRID:SCR_017973

## Availability of Supporting Data and Materials

All data are available at the provided links.

Bccr-segset dataset: https://data.pawsey.org.au/download/Weedvision/public/LBP-SVM-analysis/bccr-set/bccr-segset%20dataset.rar

Can-rad dataset: https://data.pawsey.org.au/download/Weedvision/public/LBP-SVM-analysis/bccr-set/can-rad_dataset.rar. Snapshots of our code and other supporting data can be found in the *GigaScience* repository, GigaDB [73].

## Abbreviations

DLBP: discriminative LBP; DLTP: discriminative local ternary pattern; ExG − ExR: excess green minus excess red indices; FLBP: filtered LBP; LBP: local binary pattern; NDVIs: normalized difference vegetation indices; RAM: random access memory; RBF: radial basis function; SIFT: scale-invariant feature transform; SURF: speeded-up robust features; SVM: support vector machine.

## Competing Interests

The authors declare that they have no competing interests.

## Funding

This work was supported by the Grains Research and Development Corporation (GRDC) and Photonic Detection Systems Pty. Ltd, Australia (grant No. WCA00004).

## Authors’ Contributions

V.N.T.L. and B.A. proposed and designed the study. V.N.T.L. and B.A. constructed the datasets and wrote and optimized the code. V.N.T.L., S.A., and K.A. wrote, revised, proofread, and improved the manuscript.

## Supplementary Material

giaa017_GIGA-D-19-00356_Original_Submission

giaa017_GIGA-D-19-00356_Revision_1

giaa017_Response_to_Reviewer_Comments_Original_Submission

giaa017_Reviewer_1_Report_Original_Submission Chris Armit -- 10/28/2019 Reviewed

giaa017_Reviewer_2_Report_Original_Submission Jayamala Patil -- 1/20/2020 Reviewed
