# Supplementary material for: A novel method for detecting morphologically similar crops and weeds based on the combination of contour masks and filtered Local Binary Pattern operators
Source: Gigascience. 2020 Mar 4;9(3):giaa017. doi: 10.1093/gigascience/giaa017 (PMC7055473; doi:10.1093/gigascience/giaa017)
Supplement: giaa017_GIGA-D-19-00356_Revision_1 [file giaa017_giga-d-19-00356_revision_1.pdf]

GigaScience

A novel k-FLBPCM method for detecting morphologically similar crops and weeds based on the combination of contour masks and Local Binary Pattern operators

--Manuscript Draft--

**Manuscript Number:**	GIGA-D-19-00356R1
**Full Title:**	A novel k-FLBPCM method for detecting morphologically similar crops and weeds based on the combination of contour masks and Local Binary Pattern operators
**Article Type:**	Research
**Funding Information:**	Grains Research and Development Corporation (WCA00004) — Professor Kamal Alameh
**Abstract:**	Weeds are a major cause of low agricultural productivity. Some weeds have morphological features similar to crops making them difficult to discriminate. This paper proposes a novel method using a combination of filtered-features extracted by combined Local Binary Pattern operators and features extracted by plant-leaf contour masks to improve the discrimination rate between broadleaf plants. Opening and closing morphological operators were applied to filter noise in plant images. The images at four stages of growth were collected using a testbed system. Mask-based Local Binary Pattern features were combined with filtered-features and a coefficient k. The classification of crops and weeds was achieved using support-vector-machine with radial basis function kernel. By investigating optimal parameters, this method reached a classification accuracy of 98.63% with four classes in the “bccr-segset” dataset published online in comparison with an accuracy of 91.85% attained by a previously reported method.
**Corresponding Author:**	Vi Nguyen Thanh Le, Ph.D<br>Edith Cowan University<br>Joondalup, WA AUSTRALIA
**Corresponding Author Secondary Information:**	
**Corresponding Author's Institution:**	Edith Cowan University
**Corresponding Author's Secondary Institution:**	
**First Author:**	Vi Nguyen Thanh Le, Ph.D
**First Author Secondary Information:**	
**Order of Authors:**	Vi Nguyen Thanh Le, Ph.D<br>Selam Ahderom<br>Beniamin Apopei<br>Kamal Alameh
**Order of Authors Secondary Information:**	
**Response to Reviewers:**	RESPONSE TO REVIEWERS<br><br>We would like to thank the Reviewers for your valuable and detailed comments. We have gone through and seriously considered every comment. We would like to summarise our response to the Reviewers’ comments as follows:<br><br>Editor’s comment: Please register any new software application in the bio.tools and SciCrunch.org databases to receive RRID (Research Resource Identification Initiative ID) and biotoolsID identifiers, and include these in your manuscript. This will facilitate tracking, reproducibility and re-use of your tool.<br>Response: We have registered an RRID account for our algorithm on SciCrunch. The RRID for this resource is: k-FLBPCM-method, RRID: SCR_017973.

Powered by Editorial Manager® and ProduXion Manager® from Aries Systems Corporation

Reviewer 1’s comments:
Comment 1: Do the authors consider the reuse potential of the k-FLBPCM method limited to classifying plant images? Alternatively, could this method be used in a Digital Pathology context to identify between, for example, epithelial and mesenchymal cells that have relatively similar morphology? A "reuse potential" section outlining the value of the k-FLBPCM method would be a useful addition to the manuscript for future researchers.
Response to Comment 1: We thank the Reviewer for your suggestion. We have now added a section in The Conclusion section from lines 500 to line 503 as follows: “Future research might consider the potential of the k-FLBPCM method in diverse applications in order to identify objects of similar morphologies. Morphological cell analysis plays a significant role in supporting pathologists to accurately detect cancer cells [1, 2]. The advantages of the k-FLBPCM method is that image data can be reused for extracting morphological features and identifying abnormal cells.”

Comment 2: The GitHub archive (https://aus01.safelinks.protection.outlook.com/?url=https%3A%2F%2Fgithub.com%2Fvinguyenle%2Fk-FLBPCM-method&data=02%7C01%7Cvlenguye%40our.ecu.edu.au%7C69e0e0c2143a453e644508d79d7c7421%7C9bcb323d7fa345e7a36f6d9cfdbcc272%7C1%7C0%7C637151030165905119&sdata=tRM0OVS92c5jAbj7l9w4dAp5CrwuDR46EsL5rGuqf2o%3D&reserved=0) does not appear to have a license associated with it. Will the authors be ascribing an OSI-approved license to this GitHub archive?
Response to Comment 2: We thank the Reviewer for your advice. We have added a General Public License (GPL) to our project on Github (https://github.com/vinguyenle/k-FLBPCM-method).

Comment 3: To encourage reuse of the image data, I recommend that the bccr-segset dataset is archived in the GigaScience DataBase (GigaDB). Does GigaScience have permission to archive the bccr-segset image dataset?
Comment 4: To encourage reuse of the image data, I recommend that the can-rad dataset is archived in the GigaScience DataBase (GigaDB). Does GigaScience have permission to archive the can-rad image dataset?
Response to Comments 3 and 4: Since the data has already been an open-source dataset, you can archive the bccr-segset and can-rad image dataset in the GigaScience DataBase.

Reviewer 2’s comments:
Comment 1: The methods used for investigation which includes LBP variants, Morphological processing and SVM Classifier are well described.
Comment 2: Conclusions are adequately supported by the experimental results.
Comment 4: Minor language editing may be possible. It does not require a heavy editing for language and clarity.
Response to Comments 1, 2 and 4: We thank the Reviewer for your comments.

Comment 3: Quality of language in the manuscript is good. However grammatical corrections are needed at some lines as below: Line 97: correct sentence grammatically. Line 246: 'However, there is some differences between': it may be: However, 'there are some differences between'.
Response to Comment 3: We thank the Reviewer. We have corrected all grammatical errors, as requested.

Comment 5: Reason behind selecting following statistical parameters should be mentioned: C =1, 10, 30, 60, 100, 1000, γ =10^−4, 10^−5, 10^−6, 10^−7and k =0.1, 0.2, 0.5, 0.7, 0.8, and 1.0.
Response to Comment 5:
According to the article [3], various pairs of (C, γ) values were tried and the ones yielding the best cross-validation accuracy were chosen. Good results were obtained with exponentially growing sequences of C and γ, and that is why we initially tried C= 〖10〗^0, 〖10〗^1, 〖10〗^2, 〖10〗^3 and γ = 〖 〖10〗^(-4), 〖10〗^(-5),10〗^(-6), 〖10〗^(-7). When we fine-tuned the parameters C and γ in these ranges, the experimental results showed that C =10, 100 and γ = 〖 〖10〗^(-5),10〗^(-6) produce

the highest classification accuracies. Then, we randomly changes the value of C within the range [10, 100] and γ within the range [〖 〖10〗 ^(-6),10〗 ^(-5)] in order to optimise the accuracy.
The reason why we chose a coefficient k was discussed in the following paragraph (lines 328 to 336): “Due to the high bin values in the FLBP method, as shown in Figure 4 and Figure 5, cmask_features are scaled by multiplying pass_features by coefficient k in k-FLBPCM method. For example, Table 1 shows the distributions of patterns (bin values) in a typical canola image. It demonstrates that by combining the pass_features (in FLBP method) and cmask_features (in FBLPbCM method), the bin values of the k-FLBPCM method have better balance between these two feature sets. The purpose of multiplying coefficient k (k≤1) with pass_features is to reduce the gap between the bin values of the cmask_features and pass_features.” Moreover, we selected k randomly from 0.1 to 1 and tested all these values in the experiments in order to observe the variation of values and chose an optimal set k, C and Gamma when these parameters reach the highest classification accuracy.

Comment 6: Line 310: Fig. 2.4 (a-d),…where is this figure, I didn't found it.
Response to Comment 6: We thank the Reviewer for pointing out some typos, which have now been corrected. We have modified all Figures using similar formats throughout the manuscript. In addition, we have highlighted all the corrections in our revised manuscript.

References
1.Kalinin AA, Allyn-Feuer A, Ade A, Fon G-V, Meixner W, Dilworth D, et al. 3D shape modeling for cell nuclear morphological analysis and classification. Scientific reports. 2018;8 1:1-14.
2.Chen S, Zhao M, Wu G, Yao C and Zhang J. Recent advances in morphological cell image analysis. Computational and mathematical methods in medicine. 2012;2012.
3.Hsu C-W, Chang C-C and Lin C-J. A practical guide to support vector classification. Taipei, 2003.

Additional Information:	
**Question**	**Response**
Are you submitting this manuscript to a special series or article collection?	No
**Experimental design and statistics**<br><br>Full details of the experimental design and statistical methods used should be given in the Methods section, as detailed in our Minimum Standards Reporting Checklist. Information essential to interpreting the data presented should be made available in the figure legends.<br><br>Have you included all the information requested in your manuscript?	Yes
**Resources**<br><br>A description of all resources used, including antibodies, cell lines, animals and software tools, with enough information to allow them to be uniquely	No

Powered by Editorial Manager® and ProduXion Manager® from Aries Systems Corporation

identified, should be included in the Methods section. Authors are strongly encouraged to cite Research Resource Identifiers (RRIDs) for antibodies, model organisms and tools, where possible.<br><br>Have you included the information requested as detailed in our Minimum Standards Reporting Checklist?	
If not, please give reasons for any omissions below.<br><br>as follow-up to "**Resources**<br><br>A description of all resources used, including antibodies, cell lines, animals and software tools, with enough information to allow them to be uniquely identified, should be included in the Methods section. Authors are strongly encouraged to cite Research Resource Identifiers (RRIDs) for antibodies, model organisms and tools, where possible.<br><br>Have you included the information requested as detailed in our Minimum Standards Reporting Checklist?<br><br>"	I uploaded the code of my method on Github.
**Availability of data and materials**<br><br>All datasets and code on which the conclusions of the paper rely must be either included in your submission or deposited in publicly available repositories (where available and ethically appropriate), referencing such data using a unique identifier in the references and in the "Availability of Data and Materials" section of your manuscript.<br><br>Have you have met the above requirement as detailed in our Minimum Standards Reporting Checklist?	Yes

Powered by Editorial Manager® and ProduXion Manager® from Aries Systems Corporation

Powered by Editorial Manager® and ProduXion Manager® from Aries Systems Corporation

Manuscript with DOI

Click here to access/download;Manuscript;Manuscipt2_VNTLe_gigascience

A novel k-FLBPCM method for detecting morphologically similar crops and weeds based on the combination of contour masks and Local Binary Pattern operators

Vi Nguyen Thanh Le[1], Selam Ahderom[1], Beniamin Apopei[1], Kamal Alameh[1]

[1] Electronic Science Research Institute, Edith Cowan University, Western Australia, Australia

*** Correspondence:**
Corresponding Author
vlenguye@our.ecu.edu.au

Abstract

Weeds are a major cause of low agricultural productivity. Some weeds have morphological features similar to crops making them difficult to discriminate. This paper proposes a novel method using a combination of filtered-features extracted by combined Local Binary Pattern operators and features extracted by plant-leaf contour masks to improve the discrimination rate between broadleaf plants. Opening and closing morphological operators were applied to filter noise in plant images. The images at four stages of growth were collected using a testbed system. Mask-based Local Binary Pattern features were combined with filtered-features and a coefficient k. The classification of crops and weeds was achieved using support-vector-machine with radial basis function kernel. By investigating optimal parameters, this method reached a classification accuracy of 98.63% with four classes in the "bccr-segset" dataset published online in comparison with an accuracy of 91.85% attained by a previously reported method.

Keywords: Precision agriculture; Morphological operators; Feature extraction; Local Binary Patterns; Contour masks; Plant classification; Computer vision.

Introduction

Weed infestation poses a threat to the environment, crop yields and quality. Weeds in a field retard crop growth by competing for access to sunshine, water and nutrients. In particular, the density, spreading

time and growth characteristics are important factors for weed management [1]. One of the most invasive and serious weeds is wild radish, which causes significant crop yield losses and low-quality crops due to its fast growth rate, contaminants, multiple-herbicide resistance and vigorous competition [2-4]. Currently, blanket herbicide spraying is the most common practice used to eradicate weeds. However, the excessive use of herbicides has negative impacts on the environment in addition to the development of herbicide-resistance properties in weeds. The dramatic challenge for controlling weeds is to attain an optimal eradication efficacy with minimum herbicide usage. Note that, reducing the herbicide application rates brings down the cost of weed management. Hence, it is a worthwhile objective in precision agriculture.

Spraying selective weeds automatically in vegetation fields is considered as a potential method to reduce the environmental and economic costs of weed management. Wild radish is a dominant weed in all broadacre field crops, including wheat, barley, sorghum, maize and canola. Canola is the most difficult crop to discriminate against wild radish because of their morphological similarity [5]. Therefore, canola, corn and wild radish are selected for experimental investigation in this study. Classifying crops and wild radish plants is a vital practical problem in agriculture. The ability to accurately detect and classify weeds in row crops in real time enables the selective application of herbicides, thus enhancing the quality and productivity of crops.

There have been numerous studies on weed-from-crop discrimination. Spectral techniques based on the calculation of the Normalised Difference Vegetation Indices (NDVIs) [6, 7] have long been proposed for identifying plant species. However, this method has some deficiencies. In typical farm field conditions, the wind, shadowing, and soil background brightness may change the spectral features of plants, leading to the reduction of the discrimination accuracy of NDVI-based weed sensors [8, 9]. Due to the drawbacks of such spectral-reflectance sensors, research on spatial sensors based on the use of image processing techniques for the classification of plant species and weeds in real time have been conducted [10]. One such spatial technique is “texture analysis” in image processing, which has been applied in many fields, such as industrial inspection systems, medical image analysis, face recognition and content-based image retrieval [11]. There are significant challenges in image texture analysis, such

as noise sensitivity, grey scale variation, rotation sensitivity and illumination and brightness conditions. One of the discriminative and computationally effective local texture descriptors that can potentially overcome these issues is local binary patterns (LBP) [12-14]. The important role of extracting dominant features is emphasized, as poor features combining with even the best classifier are unlikely to achieve good identification results.

In this paper, the LBP method is applied to extract plant features due to its flexibility and robustness in monotonic grey-level transformation, illumination, scaling, viewpoint, and rotation variance. Furthermore, the LBP method is also a robust tool for identifying the relationship among the pixels in plant images and detecting microstructures including lines, spots, edges and flat areas [14]. Another attractive feature of the LBP method is low computational complexity [15]. In fact, the LPB is computationally less complex than its SIFT or SURF counterparts [16]. Finally, it has exhibited superior performance in various applications, such as motion analysis [17, 18], texture recognition [12, 14, 19], face recognition [20-22], face expression analysis [23, 24], fingerprint recognition [25] and image retrieval [26, 27].

Numerous studies on the LBP method have been developed to enhance its discriminative power including Completed LBP [12], Extended LBP [28, 29], Discriminative completed LBP [30], Dominant LBP with Gabor filtering features [19], Pairwise rotation invariant co-occurrence LBP [31], Fuzzy LBP [32], Robust LBP [33], Noise-tolerant LBP [34] and Noise resistant LBP [35]. However, these methods still have unsatisfying tolerance to noise in images and increased feature dimensionality, leading to high computational complexity [36].

In the agricultural context, the complex and similar morphologies of plant leaves are one of the key challenges to find effective and discriminative plant descriptors. Combining LBP features with other features from different methods has become an interesting research topic in plant recognition. There have been several approaches based on applying the LBP method for the identification and classification of plants. For example, using LBP, in conjunction with template matching and SVM, was proposed to classify broadleaf and grass weed images [37]. These weed images having broad and narrow leaf shapes were easily distinguished. Similarly, another study on combining LBP, Local Ternary Pattern and Local

Directional to classify broadleaf and narrow grass weeds [38]. Another statistical method for separating sugar beets and weeds has been proposed, based on using shape features [39]. However, this method was considered accurate only because the sugar beet sizes were significantly different from those of the weeds. The LBP method has also been used for crop segmentation in order to detect occluded crops (sweet pepper) [40]. However, the detection accuracy was quite limited (just 66.8%). The detection and classification of apple fruit diseases using Global Colour Histogram, Colour Coherence Vector, LBP and Complete LBP has been investigated [41]. The classification accuracy of this method was just above 93%. Identifying medicinal plants was conducted by combining morphological, LBP variance and colour features and the classification accuracy of this method was 72.16% [42]. In addition, canola, corn and radish plants have been classified using the combined LBP operators and SVM with a classification accuracy of 91.85% [43]. These methods are still deemed unsatisfactorily due to their low classification accuracy.

Some studies have investigated a promising approach to reducing noise and increasing classification accuracy is the combination of the LBP operators and contours that mask LBP images. LBP-guided active contour approaches have only been proposed for texture segmentation [44]. The active contour can identify the position of the initial curve anywhere in the captured image and then automatically detect interior contours. By combining scalar and vector LBP active contours, reduced computational cost and high segmentation quality can be achieved. However, typically, this method has been applied in the segmentation process. LBP-based edge-texture features for object recognition has also been proposed [45]. Particularly, discriminative LBP (DLBP) and Local Ternary Pattern (DLTP) were focused on differentiating a bright object against a dark background by combining edge and texture information. Another method for detecting humans based on non-redundant LBP shape descriptor has been implemented by concatenating a set of local appearance descriptors extracted at a set of key points. However, occlusion was the main limitation that made this method impractical [46]. Another LBP edge-mapped descriptor for face recognition has been investigated [47], whereby LBP was applied on the edge contours (eyes, nose, and mouth) instead of the whole image, then the LBP intensity was combined with the edge pixel array around the feature points.

The above-mentioned methods have their own drawbacks, such as having unsatisfactory classification accuracy, computational complexity, application-specific recognition and not dealing with occlusion. In the context of this paper, we address the challenge of discriminating broadleaf plants species of relatively-similar morphology by proposing a novel method called "filtered LBP method with contour mask and coefficient k (k-FLBPCM)", which enhances the plant discrimination capability. The k-FLBPCM is based on combining filtered LBP features and contour mask-based features to precisely identify and classify broad-leaf plants in the field. The current k-FLBPCM method has particularly been applied for the classification of two broad-leaf plants, namely canola (crop) and wild radish (weed), which significantly improves on the accuracy of our previously published paper [43]. This paper still employs a support vector machine (SVM) classifier due to its good accuracy and relevance to real-life datasets [48, 49]. The "bccr-segset" dataset, which comprises a variety of plant images at four defined growth stages, with rotation, scale and viewpoint variance, is used in this paper in order to compare the present results with our previously reported results.

Morphological operations

The Excess Green minus Excess Red Indices (ExG-ExR) method was used to segment green plant regions in the bccr-segset dataset [43]. During segmentation, the noise in plant images creates issues in the process of edge detection. However, reducing the noise level in these plant images plays an important role in image enhancement for the next stages of feature extraction and classification.

Morphological image processing is particularly investigated in this paper [50]. Morphological operators are introduced and extended to analyse images by Matheron and Serra [51]. Particularly, in morphological analysis, images are treated as sets that illustrate the plant shapes, represented in grey-scale or binary images. Morphological transformations are a tool that helps extract features from images using Minkowski addition and subtraction [52]. The morphological process needs two inputs including grey-scale images and structuring elements. The function of morphology operators is to transform from one set to another with the aim of searching the special structure of the original set. Then, the special structure information is stored in the transformed set and the transformation is recognized by special

structuring elements. As a result, there is a correlation among some characteristics of the structuring elements.

There are two basic morphological operations for binary and grey-scale images including erosion and dilation. Erosion is defined as a shrinking transformation, which reduces the size of regions within the image, while expanding the size of holes within the regions. As for dilation, it is defined as an expansion transformation, which increases the size of the regions within the image while reducing the size of the holes in the regions and gaps between the regions. It is important to note that the erosion operator filters the inner image, while the dilation operator filters the outer image. Opening and closing morphological operators, which are an extension of erosion and dilation operators are also used, to find specific shapes in an image. Specifically, the opening operation comprises the erosion operation followed by the dilation operation, and helps to smooth the contour of an image and eliminate small objects. On the other hand, the closing operation tends to remove small holes and fill gaps in the contours [53]. Note that morphological operations have gained popularity because they are useful for the detection of the edge of an image and suppression of noise.

In this paper, opening and closing morphological operators are applied on grey-scale images, mainly to filter noise [53], while erosion and dilation operations are used for processing image edges. I(x,y) is considered as a grey-scale two-dimensional image and S is referred as structuring element. The erosion of a grey-scale image I(x,y) by a structuring element S(a,b) is defined as [52, 54]:

$$I \ominus S = \min\{I(x+a, y+b) - S(a,b)\} \quad (1)$$

The dilation of a grey-scale image, I(x,y), is denoted by

$$I \oplus S = \max\{I(x-a, y-b) + S(a,b)\} \quad (2)$$

Based on the erosion and dilation operators, the opening and closing of the image I by the structuring element S are respectively defined as follows:

$$I \circ S = (I \ominus S) \oplus I \quad (3)$$

$$I \bullet S = (I \oplus S) \ominus S \quad (4)$$

In this paper, the first step is to select structuring elements which are regarded as matrices and able to measure the shape of the image. In addition, choosing the shape and size of the structuring element is based on the condition and processing demand of the image. In this paper, we used a 5×5 square structuring element to input in the opening and closing morphological operators for filtering. The opened and closed images were then converted to binary images by using thresholds for next features extraction and classification processes.

Local Binary Pattern Operators

The LBP algorithm was introduced by Ojala et al. in 1996 [55]. The LBP operator has been developed to detect textures or objects in images for a long time. It is considered a robust texture descriptor for analysing images, because of its capability to represent plant discriminative information and computational efficiency [55]. It is also one of the best texture descriptors and has been effectively used in various applications. The potentials and effectiveness of LBP have been presented in identifying objects, recognizing faces and facial expressions and classifying demographics. In this paper, the LBP operator is particularly used for leaf description due to its effectiveness in pattern description.

The main limitation of the previously reported LBP operator was to only cover a small 3×3 neighbourhood, thus failing to capture dominant textural features in images with large-scale structures. To overcome this drawback (i.e., improve the LBP operators), the number of pixels and the radius in the circular neighbourhood have been increased [14]. Typically, it is more flexible and effective to enhance the performance of the LBP method by using textures of different scales. Generally, the value of the LBP code of a centre pixel (x_c, y_c) can be calculated as follows [14]:

$$\mathrm{LBP}_{P,R} = \sum_{p=0}^{P-1} s(g_p - g_c)2^p \quad \text{where} \quad s(x) = \begin{cases} 1, x \geq 0 \\ 0, x < 0 \end{cases} \tag{5}$$

where g_c is the grey value of the central pixel and g_p indicates the grey values of the circularly symmetric neighbourhood from $p = 0$ to $P - 1$ and $g_p = x_{P,R,p}$. In addition, P stands for the number of surrounding pixels in the circular neighbourhood with the spatial resolution of the neighbourhood R. Also, $s(x)$ symbolizes the thresholding function, which helps the LBP algorithm to gain illumination

invariance against any monotonic transformation. The probability distribution of the 2^{P} LBP patterns represents the characteristic of the texture image. The mentioned parameters of the LBP algorithm control how patterns are computed for each pixel in input images.

Rotating an image causes diverse LBP codes. Therefore, LBP codes need to rotate back to the position of the reference pixel in order to invalidate the results of translating a pixel location and generate multiple identical versions of binary codes. To address the problem of the image rotation effect, a rotation-invariant LBP has been defined as follows [14, 56]:

$$LBP_{P,R}^{ri} = \min\{ROR(LBP_{P,R}, i) \quad | \quad i = 0, 1, \ldots, P-1\} \tag{6}$$

where the function $ROR(x, i)$ performs an i-step circular bit-wise right shift on the P-bit number x. The rotation invariant LBP is formed by circularly rotating the basic LBP code and keeping the rotationally-unique patterns that result in a significant reduction in feature dimensionality.

For uniform patterns, $LBP_{P,R}$ refers to the number of spatial transitions in the patterns and the $LBP_{P,R}^{u2}$ patterns need to have at most two bitwise transitions from 0 to 1 or vice versa. As for a given pattern of P bits, the uniform descriptor produces $P\,(P-1)+3$ output bins, which consist of $P\,(P-1)+2$ bins for distinct uniform patterns, and a single bin $(P+1)$ assigned to all non-uniform patterns. To overcome poor discrimination, due to the crude quantization of angular space at 45° intervals, the rotation invariant uniform descriptor $LBP_{P,R}^{riu2}$, which has a U value of at most 2, is defined as follows [14]:

$$LBP_{P,R}^{riu2} = \begin{cases} \sum_{p=0}^{P-1} s(g_p - g_c), & \text{if } U(LBP_{P,R}) \leq 2 \\ P+1, & \text{if } U(LBP_{P,R}) > 2 \end{cases} \tag{7}$$

The other patterns are marked as “miscellaneous” label and grouped into a single value. To map from $LBP_{P,R}$ to $LBP_{P,R}^{riu2}$, the number of bins depends on the number of neighbours P are P+2. Correspondently, the $LBP_{8,1}^{riu2}$, $LBP_{16,2}^{riu2}$ and $LBP_{24,3}^{riu2}$ operators have 10, 18 and 26 bins, respectively.

Support Vector Machines (SVM)

After the dominant features are extracted using the LBP method, the next stage is classification. There are several different classification methods, including decision trees, SVM, neural networks, k-nearest neighbour method and the Bayesian classifier. One of the efficient classification methods is SVM, due to its high performance in many applications, such as face recognition [57, 58], weed identification [59, 60] and disease detection in plant leaves [61, 62]. Therefore, the optimal combination of the LBP descriptors and the SVM classifier can lead to high plant discrimination accuracy. Furthermore, the SVM method has become widespread for classifying objects. It is also regarded as an effective and robust supervised classifier due to its capability of dealing with pattern recognition problems in image processing and preventing over-fitting and noise data [63, 64]. SVM was originally introduced in 1992 [65] and then significantly extended by many other researchers. A binary classification SVM was first proposed [66]. A given training dataset of images (x_i, y_i) where $x_i \in \mathbb{R}^d$ for i = 1, 2, 3… N (images) with a label $y_i \in \{-1, 1\}$, the SVM binary classifier $f(x)$ predicts a label y as follows [66]:

$$f(x_i)\begin{cases}\geq 0 & y_i = +1\\ < 0 & y_i = -1\end{cases} \tag{8}$$

For example, $y_i\, f(x_i) > 0$ is considered as a correct classification. The optimization problem solved for binary classification is formulated as follows [65, 67]:

$$\min_{w,b,\xi} = \frac{1}{2}w^T w + C\sum_{i=1}^{l}\xi_i \tag{9}$$

subject to the constraint $y_i\left(w^T\phi(x_i) + b\right) \geq 1 - \xi_i$ with $\xi_i \geq 0, i = 1, \ldots, l$

According to Eq. (9), the training data x_i are mapped into a higher dimensional space by the function ϕ and every constraint can be satisfied if ξ_i is sufficiently large. In addition, C > 0 is the regularization parameter, w is known as the weight vector and b is the bias. The SVM method generates an optimal hyperplane with the maximal margin between classes in the higher dimensional space. A kernel function $K(x_i, x_j)$ is represented as $\phi(x_i)^T\phi(x_j)$ and two kernels including polynomial and radial basis function (RBF) are applied in this paper. The polynomial and RBF kernels with kernel parameters γ, r, d are given by [68]

$$\text{Polynomial SVM: } K(x_i, x_j) = (\gamma x_i^T x_j + r)^d, \gamma > 0 \quad (10)$$

$$\text{RBF SVM: } K(x_i, x_j) = \exp(-\gamma \|x_i - x_j\|^2), \gamma > 0 \quad (11)$$

Kernel selection has long been a problem. In this paper, a study is conducted using independent test sets to compare kernels and select the best one.

Data Collection

As mentioned in the article [43], all data was captured on a custom-built testing facility in Figure 1 at ESRI (Electron Science Research Institute), Edith Cowan University, Australia. Particularly, a Xilinx Zynq ZC702 development platform [65] captured HD images (1920×1080 pixels) at 60 frames per second and used an On-Semi VITA 2000 camera sensor. All images captured by the camera had a spatial resolution of ≈1mm/pixel and size of 228×228 pixels, which were down-sampled by a factor of 2 from a size of 456×456 pixels. Moreover, the vertical height of the camera above the surface of the plant pots was 980 mm and the camera focal length was 9mm.

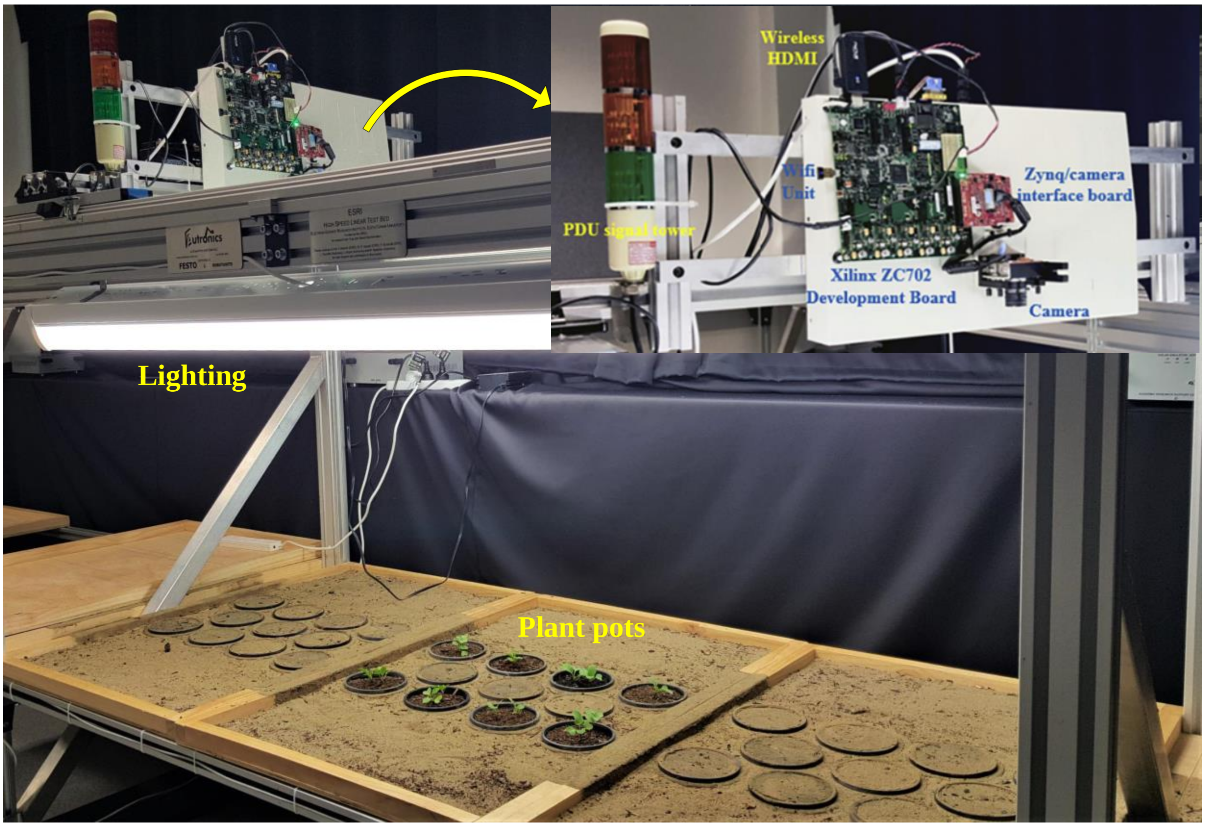

Figure 1: A high-speed testbed system used for controlled data capture [43]

In this paper, we continue to use the bccr-segset dataset to compare the performance of the novel combination of the LBP algorithm and contoured mask with coefficient k with that of the combined LBP operators reported in [43]. In addition, a new dataset of broadleaf images including only canola and radish leaves is captured to objectively evaluate the detection capability of the proposed approach.

Method

In the previous paper [43], three different LBP operators $LBP_{8,1}^{riu2}, LBP_{16,2}^{riu2}$ and $LBP_{24,3}^{riu2}$ and the SVM method were combined to detect and classify broadleaf and narrow-leaf plants. The results confirmed that the classification accuracies between broad and narrow leaves were higher than the ones between broadleaf groups. The recognition of leaves is based on the observation of their morphological features such as texture and shape. According to our “bccr-segset” dataset, canola and radish plants belong to the broadleaf group, develop as a rosette and have lobes. However, there are some differences between leaf shapes on the canola and radish plants. When the edge of each leaf is observed closely at the third stage in Figure 2, canola leaves have outward-pointing teeth and radish leaves have a rounded shape with curved-toothed edge. In other words, from the glossary of leaf morphology, the leaf margin of canola is sinuate while the edge of radish is undulate with a wavy edge, shallower than sinuate [69]. For canola leaves at the fourth growth stage, their lobes are often completely separated towards the base of the leaf. With regard to older radish leaves, they have larger rounded lobe at the tip of the leaf, some pairs of side lobes and each set is progressively smaller toward the base.

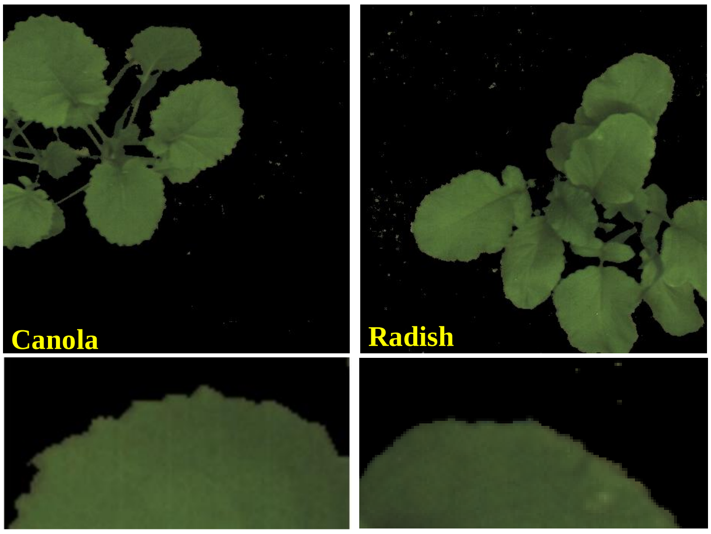

Figure 2: Full and zoomed-in images of canola and radish leaves in the third stage.

To overcome the limitation of the combined LBP operators in the previous paper, a novel method has been developed for amplifying the dominant features of canola and radish leaves. The flowchart below describes this method in detail.

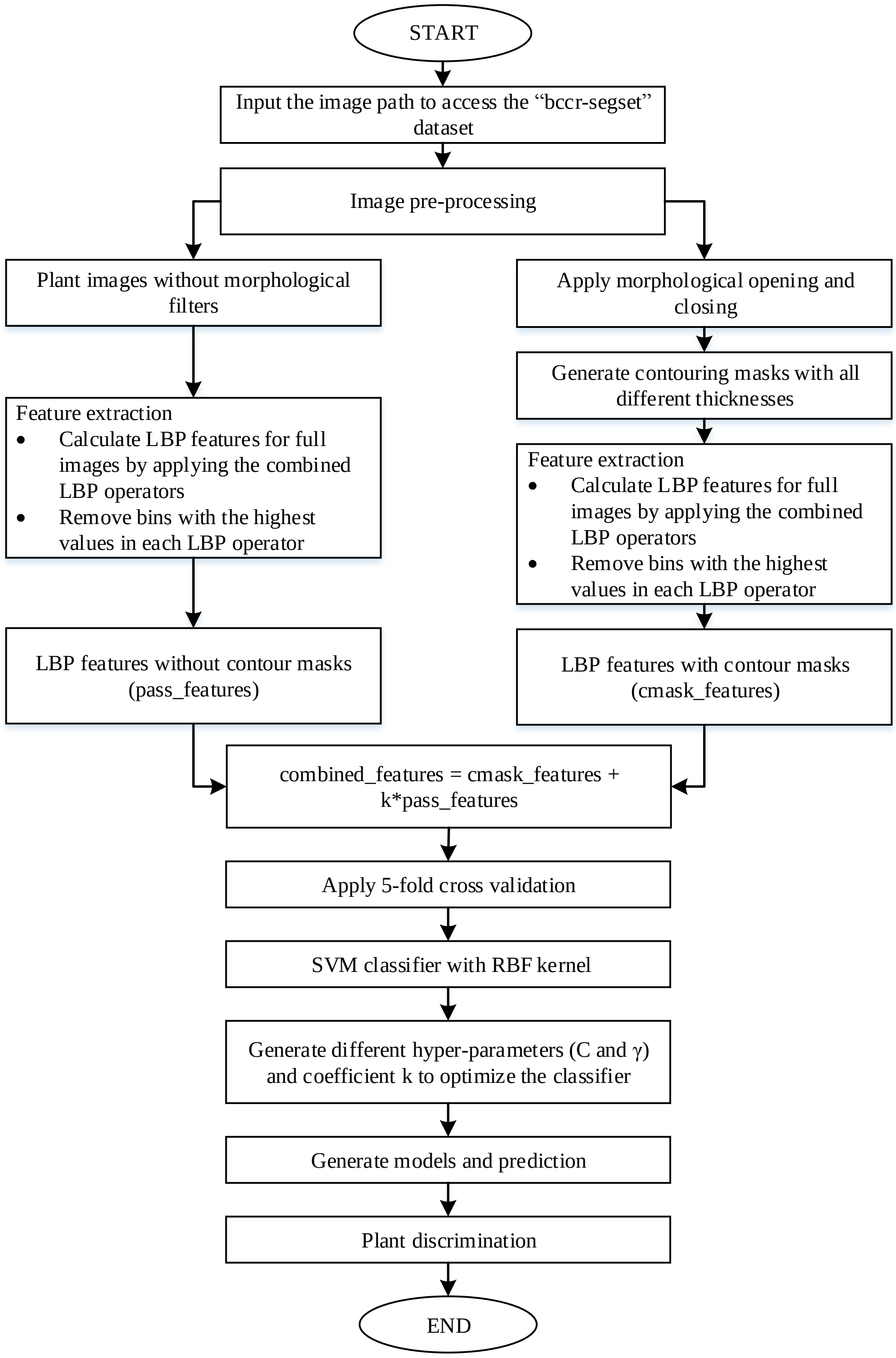

Figure 3: A flowchart describing the procedures of the novel method through steps, namely, filtering LBP bins, extracting features, masking images based on contours and classifying plant leaves.

To begin with, we input the “bccr-segset” dataset into the plant classification program. The dataset was processed in two branches: (i) the dataset was input to the feature extraction block without applying the morphological operations, and (ii) the dataset applied the morphological opening and closing, and generated contour masks with different thicknesses as shown in Figure 3. To be more specific in the second branch, a 5x5 morphological filter was created to implement the morphological opening and closing on all plant images in the dataset. By selecting a threshold, grayscale images were converted into binary images to get better accuracy. Here, we masked all plant images with contours, i.e., boundaries around selected plant images. The *findContours* function and *drawContours* function in OpenCV were used, and then all the masks of plant images of different thicknesses were stored. This eliminates the need to recalculate when the thickness was changed.

The next stage of both branches was going through the feature extraction block. Particularly, LBP features were computed for full images in the mentioned dataset by incorporating $\mathrm{LBP}_{8,1}^{\mathrm{riu2}} + \mathrm{LBP}_{16,2}^{\mathrm{riu2}} + \mathrm{LBP}_{24,3}^{\mathrm{riu2}}$ operators, which are accumulated into a histogram of P+2 bins (with P=8, 16, 24 corresponding to each LBP operator). Each bin denotes an estimate of the probability of encountering the corresponding pattern in the plant image. The discrete histograms of the $\mathrm{LBP}_{\mathrm{P,R}}^{\mathrm{riu2}}$ operators were calculated over plant images. Note that it is not necessary for all bins in the LBP histogram to contain useful information for plant leaf detection. It is observed that for the LBP histograms of plant images at the bin level, the 9th bin of $\mathrm{LBP}_{8,1}^{\mathrm{riu2}}$, the 17th bin of $\mathrm{LBP}_{16,2}^{\mathrm{riu2}}$ and the 25th bin of $\mathrm{LBP}_{24,3}^{\mathrm{riu2}}$ contain a much higher number of hits when compared to the remaining bins from the LBP histogram. A further investigation shows that the LBP values for these bins correspond to patterns which have no pixel variations. For example, all pixels are constant values such as the values of background pixels. However, the remaining bins correspond to LBP patterns which mainly capture the intensity variations of green pixels (plant leaves). Therefore, bins P+1 (the 9th bin of $\mathrm{LBP}_{8,1}^{\mathrm{riu2}}$, the 17th bin of $\mathrm{LBP}_{16,2}^{\mathrm{riu2}}$ and the 25th bin of $\mathrm{LBP}_{24,3}^{\mathrm{riu2}}$) were removed from each LBP histogram in order to better scale the remaining bins. According to the combination of three different spatial resolutions and different angular resolutions in LBP operators, three bins including 9th, 27th and 53rd were removed in the joint histogram of $\mathrm{LBP}_{8,1}^{\mathrm{riu2}} + \mathrm{LBP}_{16,2}^{\mathrm{riu2}} + \mathrm{LBP}_{24,3}^{\mathrm{riu2}}$ operator (10 bins +18 bins + 26bins = 54 bins). After applying the

$LBP_{8,1}^{riu2} + LBP_{16,2}^{riu2} + LBP_{24,3}^{riu2}$ operator for the plant images, the resultant images were called as LBP images.

As can be seen in Figure 4, it illustrates an example of the process shown in the flowchart (Figure 3). In Figure 4 a) we show an original canola leaf image and its three histograms corresponding to $LBP_{8,1}^{riu2}, LBP_{16,2}^{riu2}$ and $LBP_{24,3}^{riu2}$ operators. The 9th, 17th and 25th bins in each operator have the highest level of the distribution of patterns. The LBP-based canola leaf image and contour mask, the original histogram and the filtered histogram of the contour masks are shown in Figure 4 b), c), d) with the $LBP_{8,1}^{riu2}, LBP_{16,2}^{riu2}$ and $LBP_{24,3}^{riu2}$ operators, respectively. It is apparent that the feature distribution is easily observed in the other bins of the LBP histogram with bin removal. Interestingly, dominant features such as edge and corner patterns in other bins can be seen clearly by removing some specific bins (9th, 17th, and 25th bins) in the LBP histograms. Similarly, plant features in the histogram of the LBP based contour mask with bin removal also present their significance. It is noted that the bin number of the LBP histogram in Figure 4, calculated in a Python code, has an index range from 0 to [(P+2) - 1] bins. Note that the bin number mentioned in this paper starts from 1 to P+2. For example, the $LBP_{8,1}^{riu2}$ operator has an index range from 0 to 9 but the bin number from 1 to 10.

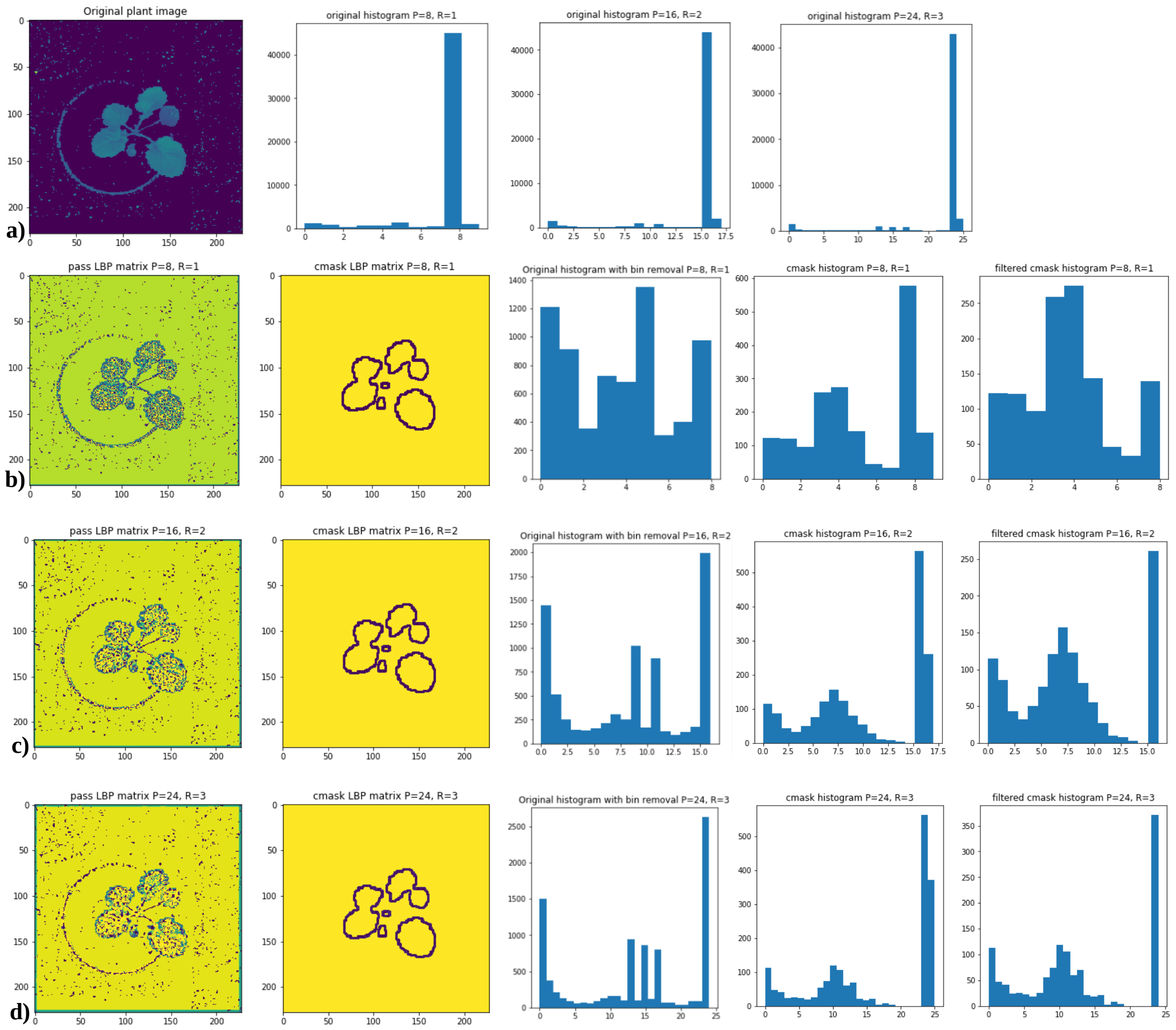

Figure 4: a) An original canola leaf image and its LBP histograms corresponding to $LBP_{8,1}^{riu2}$, $LBP_{16,2}^{riu2}$ and $LBP_{24,3}^{riu2}$ operators **b), c)** and **d)** LBP images, LBP images with contour masks, and their original LBP histograms and filtered LBP histograms are presented by implementing $LBP_{8,1}^{riu2}$, $LBP_{16,2}^{riu2}$ and $LBP_{24,3}^{riu2}$ operators, respectively.

Multiresolution analysis can be achieved by altering P and R of LBP operators and then combining these operators. Figure 5 (a-d), shows four different LBP histograms of a canola leaf image obtained by combining three operators ($LBP_{8,1}^{riu2}$, $LBP_{16,2}^{riu2}$ and$LBP_{24,3}^{riu2}$), eliminating 9th, 27th and 53rd bins, applying the LBP method with contour masking and removing 9th, 27th and 53rd bins in the joint cmask histogram, respectively.

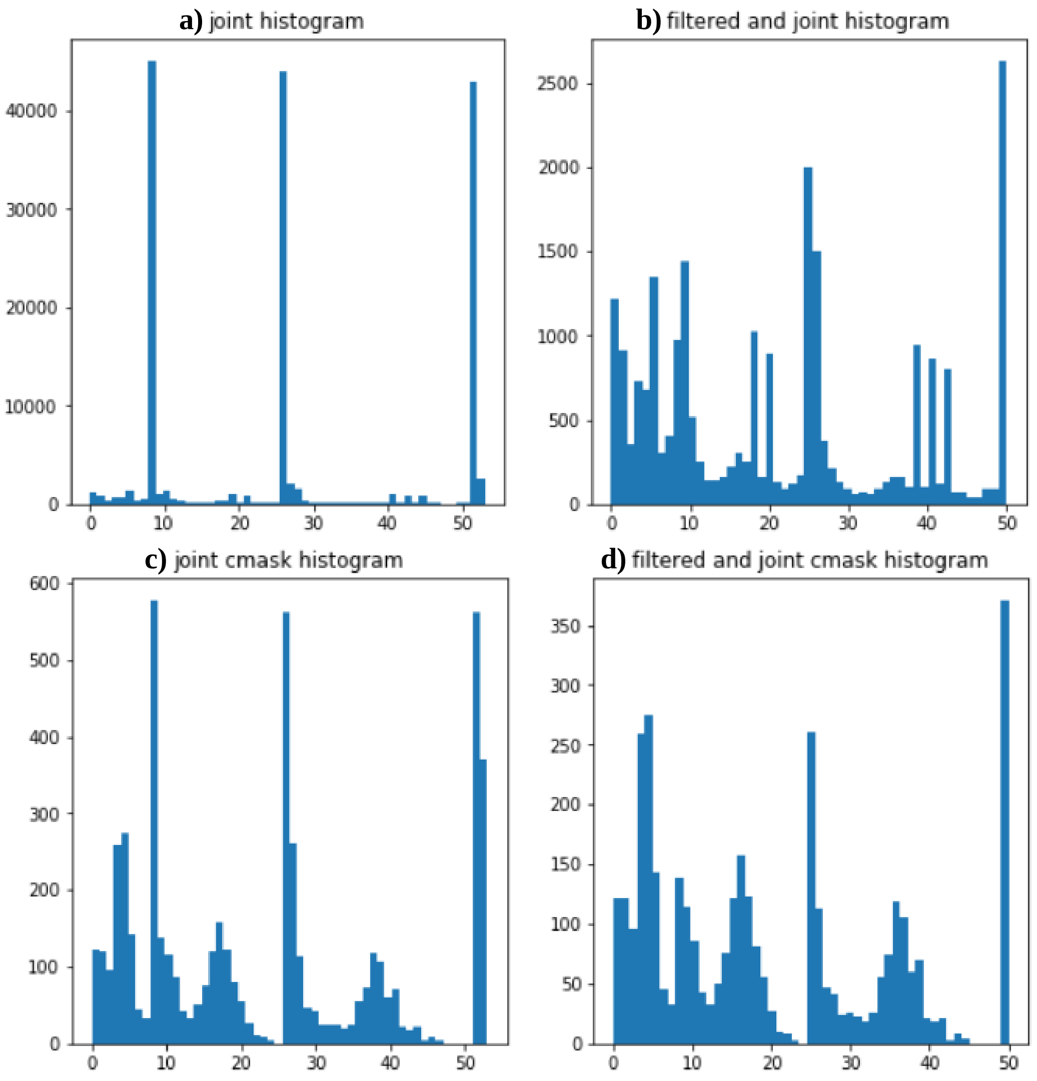

Figure 5: Four different LBP histograms of a canola leaf image. a) combining three operators ($LBP^{riu2}_{8,1}$, $LBP^{riu2}_{16,2}$ and $LBP^{riu2}_{24,3}$). b) A filtered and joint histogram is generated by eliminating 9th, 27th and 53rd bins in the joint histogram. c) A joint cmask histogram is generated by applying the LBP method with a contour mask. d) Removing 9th, 27th and 53rd bins in the joint cmask histogram.

As shown in Figure 3, the filtered LBP features without contour mask in plant images are denoted as ***pass_features***. The method used to generate images is referred to as Filtered LBP method (FLBP). The FLBP method is applied to the plant images, and results in 51 features are calculated over the entire image. The FLBP based contour masks are denoted as ***cmask_features***. The method used to create images consisting of cmask_features is referred to as the Filtered LBP based Contour Mask (FLBPbCM). Applying the FLBPbCM method to the plant images also results in 51 features computed

only on the contours. The remaining region in the image is set to the maximum value (255) in the LBP matrix and ignored when generating the LBP histogram.

The novelty of the current ***k-FLBPCM method*** filtered LBP method with Contour Mask and coefficient k (k-FLBPCM) is a combination of ***pass_features*** and ***cmask_features***. Due to the high bin values in the FLBP method as shown in Figure 4 and Figure 5, cmask_features are scaled by multiplying pass_features by coefficient k in k-FLBPCM method. For example, Table 1 shows the distributions of patterns (bin values) in a typical canola image. It demonstrates that by combining the pass_features (in FLBP method) and cmask_features (in FBLPbCM method), the bin values of the k-FLBPCM method have better balance between these two feature sets. The purpose of multiplying coefficient k ($k \leq 1$) with pass_features is to reduce the gap between the bin values of the cmask_features and pass_features.

Table 1: The bin values of a typical canola image using FLBP, LFBPbCM and the combined k-FLBPCM methods

**Bin values of different methods**	**Bin 1**	**Bin 2**	**Bin 3**	**Bin 4**	**Bin 5**	**Bin 6**	**Bin 7**	**Bin 8**	**Bin 10**
FLBP	1212	913	355	727	680	1351	305	402	974
FLBPbCM	122	121	96	259	275	143	45	33	139
k-FLBPCM with k=0.5	728	577.5	273.5	622.5	615	818.5	197.5	234	626

After the feature extraction step, the plant images are classified by using SVM kernels. Initially, 5-fold-cross validation was used to divide the dataset into five subsets. Due to the different plant growth stages in the dataset, images at each growth stage are equally divided in each subset as well. A single subset of the dataset is used for testing while the remaining four subsets of the dataset are used for training. The cross-validation process was iteratively applied five times, with the test subset changed each time. This procedure helps to prevent overfitting. After generating the training model by selecting RBF kernel in SVM and making predictions, the classification accuracies of the methods was calculated by using the performance metrics such as accuracy, precision, recall and F1-score.

Results

The results are divided into two sections: the first section presents the average classification accuracies of the broadleaf classes consisting of canola and radish. The effectiveness of the proposed k-FLBPCM method is evaluated based on factors including feature extraction (by comparing among the FLBP, FLBPbCM, and k-FLBPCM methods), different SVM kernels (the second order polynomial kernel and RBF kernel), contour thickness, LBP parameters P (the total number of the neighbouring pixels) and R (the radius) as well as the coefficient k. In the second section, the parameters (C, Gamma (γ), coefficient k and thickness) for the classification of all four classes in the "bccr-segset" dataset including canola, corn, radish and background are optimized to obtain improved classification accuracy. The computer used in these experiments had a 3.4GHz processor, 16GB RAM and ran Python 2.7.13.

Results of the k-FLBPCM, FLBPbCM and FLBP methods in classifying two different broadleaf plants

Canola and radish images were taken from the "bccr-segset" dataset. The train and test sets of canola and radish classes consist of 15000 images (7500 images in each class). After applying the FLBP, FLBPbCM, or k-FLBPCM methods, SVM was used to classify the two broadleaf classes including canola and radish plants. The classification accuracies of the second order polynomial kernel and the RBF kernel were compared. In this experiment, C = 10, 60, γ $=10^{-5}, 10^{-6}$ and thickness =2 were selected. The values of C and γ selected were typical values, before any optimization had been performed.

The results of using two SVM kernels (the second order polynomial and RBF kernels) on the given dataset for classification are summarised in

Table **2**. In particular, the average classification accuracy of the k-FLBPCM method (C=10, γ $=10^{-5}$, k=0.5 and 0.2) with the RBF kernel was 97.32%, followed by 96.40% corresponding to k-FLBPCM method with coefficient k=0.1. Meanwhile, the average classification accuracy of the k-FLBPCM method (C=10, γ $=10^{-5}$, k=0.5) with the second order polynomial kernel was just 95.46%. Similarly, the case (C=60, γ $=10^{-6}$) of the k-FLBPCM method with the RBF kernel was also higher than the

polynomial kernel of degree 2. In addition, the FLBP method with the RBF kernel had higher classification rate than the polynomial kernel. As for the FLBPbCM method (C=10, γ =10^{-5}), the RBF kernel had the classification accuracy of 94.07% in comparison to the second order polynomial kernel at 88.53%. These results show that the RBF kernel, which nonlinearly maps features into a higher dimensional space, resulting in higher classification accuracy for all three methods (FLBP, FLBPbCM and k-FLBPCM methods).

Table 2: The average classification accuracy score of the k-FLBPCM, FLBPbCM and FLBP methods with the second order polynomial and RBF kernels.

**C**	**γ**	**Thickness**	**Methods**	**Accuracy Score**	
				**Polynomial kernel of degree 2**	**RBF kernel**
10	1E-05	2	k-FLBPCM method, k=0.5	95.46%	97.32%
10	1E-05	2	k-FLBPCM method, k=0.2	94.91%	97.32%
10	1E-05	2	k-FLBPCM method, k=0.1	94.27%	96.40%
60	1E-06	2	k-FLBPCM method, k=1	94.92%	97.50%
60	1E-06	2	k-FLBPCM method, k=0.5	94.56%	96.89%
60	1E-06	2	k-FLBPCM method, k=0.2	93.55%	96.06%
10	1E-05	No thickness	FLBP method	93.53%	95.36%
60	1E-06	No thickness	FLBP method	93.74%	96.72%
10	1E-05	2	FLBPCM method	88.53%	94.07%
60	1E-06	2	FLBPCM method	88.26%	94.83%

A second experiment was conducted to investigate the effects of the hyper-parameters C and γ, as well as the coefficient k on the classification accuracy of canola and radish images. Various pairs of (C, γ) values were tried and good results were obtained with exponentially growing sequences of C and γ [70]. Therefore, we chose the ranges of C, γ and coefficient k as follows: C =1, 10, 30, 60, 100, 1000, γ = $10^{-4}, 10^{-5}, 10^{-6}, 10^{-7}$. In addition, as mentioned in the method section, we selected k (k≤1) randomly from 0.1 to 1 (k =0.1, 0.2, 0.5, 0.7, 0.8, and 1.0). We tested all these values in the experiments in order to observe the variation of values and chose an optimal set k, C and Gamma when these parameters reach the highest classification accuracy. As shown in

Table **3**, the k-FLBPCM method had the highest classification accuracy, averaged over the 5-folds of the cross validation, in the first pair (C=30, γ =10^{-5}, thickness=2, k=0.2) and the second pair (C=60, γ =10^{-6}, thickness=2, k=1), at 97.50%. In addition, the average classification accuracies of the k-

FLBPCM method with different parameters were sorted from high to low. Due to the large number of combinations possible, only the top 10 cases are listed in Table **3**. Due to the low accuracy of using γ =10^{-4}, the parameter γ should be less than 10^{-5} to improve the classification accuracy of the k-FLBPCM method.

Table 3: The average accuracy scores of the k-FLBPCM method with the RBF kernel, varying C, γ and the coefficient k.

**C**	**γ**	**Thickness**	**k-FLBPCM method**	**Accuracy score**
30	1E-05	2	k=0.2	97.50%
60	1E-06	2	k=1	97.50%
60	1E-05	2	k=0.2	97.49%
100	1E-05	2	k=0.2	97.45%
100	1E-06	2	k=1	97.42%
30	1E-06	2	k=1	97.42%
100	1E-06	2	k=0.7	97.40%
30	1E-05	2	k=0.5	97.37%
100	1E-06	2	k=0.8	97.35%
60	1E-06	2	k=0.8	97.34%

Although all experiments were conducted with different coefficients k, this parameter should be less than or equal to 1. We find that ($k \leq 1$) results in optimal accuracy. As shown in Figure 1, the average classification accuracies of the proposed k-FLBPCM method with $k \leq 1$ were higher than the ones with $k>1$.

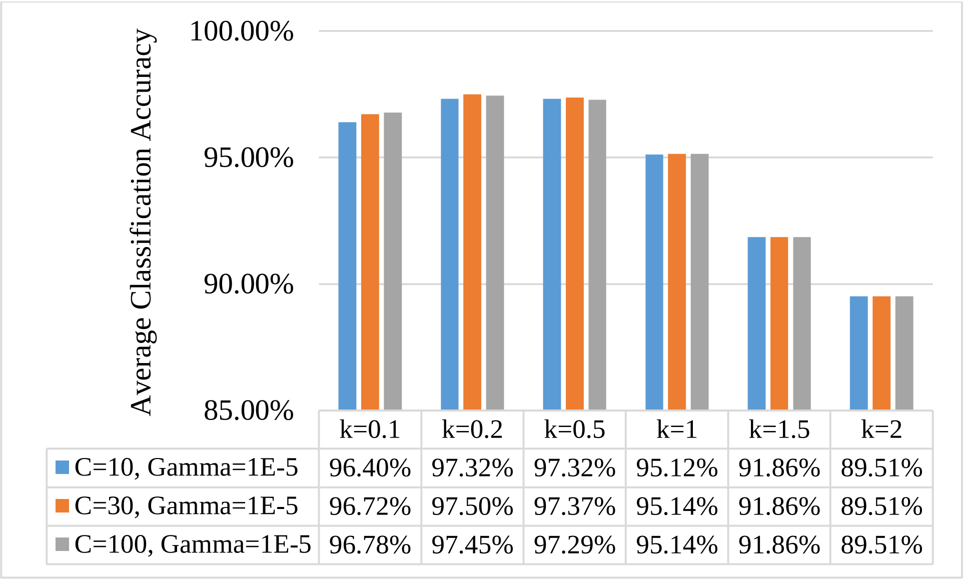

Figure 1: The average classification accuracies of the k-FLBPCM method with different coefficients k.

Comparing the FLBP, FLBPbCM, and k-FLBPCM methods

To check the effectiveness of the k-FLBPCM method in a different dataset, a new set of canola and radish images in four different growth stages was collected and designated "can-rad" dataset (published online). A total of 19600 broadleaf images (9800 images in each class) were collected at four different growth stages. The parameters C =10, 30, 60, 100, 1000, γ =$10^{-5}, 10^{-6}$, and thicknesses from 1 to 8 were selected. Note that the SVM classifier was used only with the RBF kernel in the remaining parts of experiments. Further, only the 10 highest classification accuracies for each method are listed in Tables 3.3-5 and the average classification accuracy scores are sorted from high to low.

Table 4: Classification accuracy of the FLBP method.

C	γ	Classification Accuracy of the FLBP method
**100**	**1E-06**	**95.13%**
1000	1E-06	95.03%
60	1E-06	94.96%
30	1E-06	94.92%
10	1E-06	94.31%
1000	1E-07	93.92%
10	1E-05	93.78%
30	1E-05	93.67%
60	1E-05	93.62%

100	1E-05	93.61%

Table 5: Classification accuracy of the FLBPbCM method.

C	γ	Thickness	Classification Accuracy of the FLBPbCM method
**30**	**1E-05**	**8**	**93.95%**
**30**	**1E-05**	**7**	**93.95%**
100	1E-05	2	93.94%
30	1E-05	6	93.88%
30	1E-05	5	93.88%
10	1E-05	8	93.88%
10	1E-05	7	93.88%
1000	1E-05	2	93.87%
60	1E-05	6	93.87%
100	1E-05	6	93.81%

As can be seen from Table 4 and Table 5, the classification accuracy of the FLBP method was 95.13% with C =100 and $\gamma = 10^{-6}$, while that of the FLBPbCM method was 93.95%, lower than the FLBP method. However, when combining the FLBP and FLBPbCM methods (in k-FLBPCM method), the classification accuracy was significantly higher. Table 6 shows that the highest average classification accuracy of the k-FLBPCM method was 96.21%.

Table 6: Classification accuracy of the k-FLBPCM method

C	γ	Thickness	k-FLBPCM method	Classification Accuracy
**1000**	**1E-06**	**2**	**k=0.5**	**96.21%**
30	1E-05	2	k=0.5	96.19%
10	1E-05	2	k=0.5	96.18%
30	1E-05	4	k=0.5	96.16%
30	1E-05	3	k=0.5	96.16%
60	1E-05	2	k=0.5	96.15%
10	1E-05	4	k=0.5	96.14%
10	1E-05	3	k=0.5	96.14%
30	1E-05	2	k=0.2	96.13%
30	1E-05	4	k=0.2	96.11%

Effects of the contour thickness on the classification accuracy

Next, we evaluated the average classification accuracy of the k-FLBPCM method for varying the thicknesses of the contour lines. The “can-rad” dataset was used for this investigation. We selected

C=10, 30, 100, $\gamma = 10^{-5}$, coefficient k = 0.5 and thickness from 1 to 8. As can be seen in Figure 2, two images of canola and radish with varying contour thickness are presented at the third growth stage.

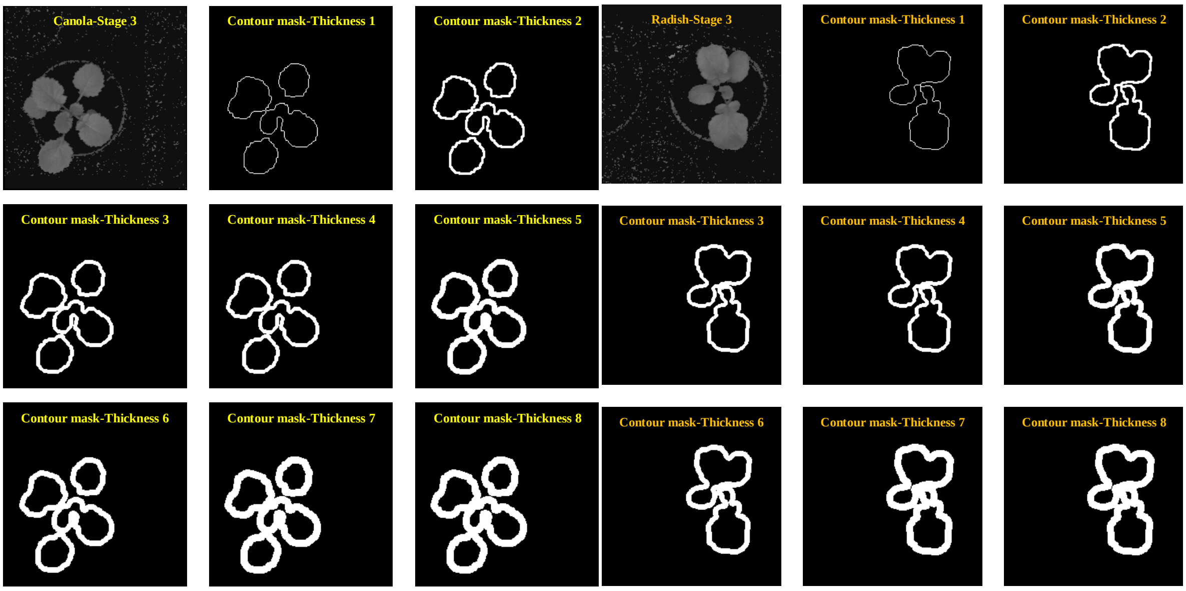

Figure 2: Canola and Radish at the third stage with varying thicknesses of the contour lines.

The average classification accuracies of the k-FLBPCM method for different thicknesses are reported in Figure 3. Our proposed k-FLBPCM method attained optimal discrimination between canola and radish at contour thickness of 2 with the accuracy of 96.19% (C=30, $\gamma = 10^{-5}$), while the lowest accuracy was 95.73% with thicknesses of 7 and 8. These two broadleaf plants displayed morphological similarity at a contour thickness of 2. As shown in Figure 2, for the thickness greater than 2, the leaf features were smoothed by the thick edge, while for the thickness of 1, the edge features were too thin to fully show the difference between the undulate and sinuate patterns.

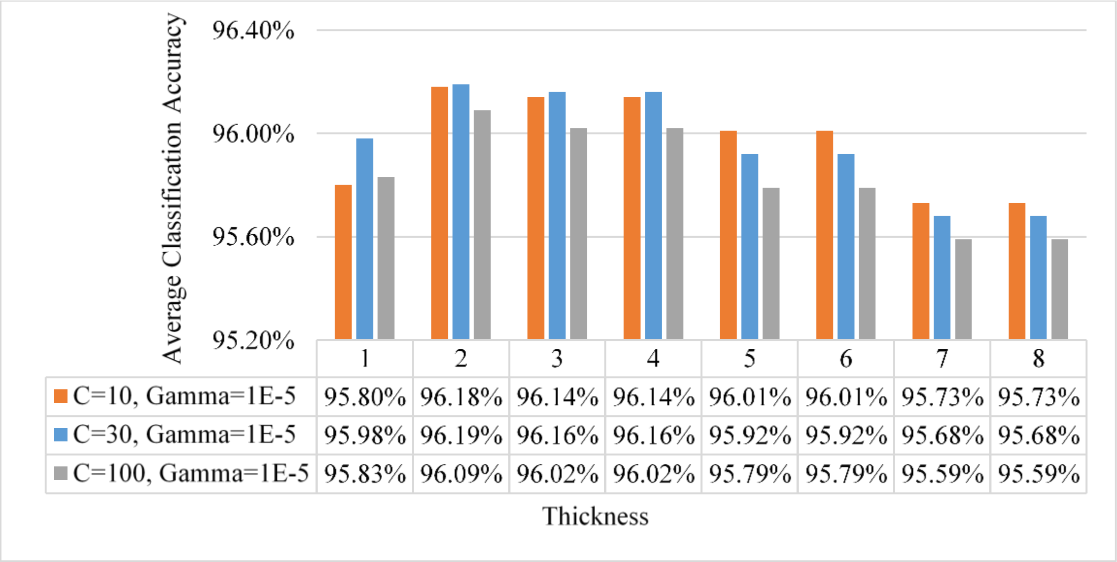

Figure 3: The average classification accuracies of the k-FLBPCM method (coefficient k=0.5) for different contour line thicknesses and four growth stages

Classification capabilities of the k-FLBPCM, FLBPbCM and FLBP methods

The k-FLBPCM method was evaluated on the full “bccr-segset” dataset, which included 30,000 plant images in four classes (canola, corn, radish and background) under different rotations, scales and illumination conditions. Plant images were taken under different rotation angles (45°, 90°, 135°, 180°, 225°, 270°, 315°, 360°), lighting conditions (sunlight and fluorescent), sizes and morphologies of plants through four growth stages, as illustrated in Figure 4. The number of plant images at each class and each growth stage is indicated in Figure 4 [43].

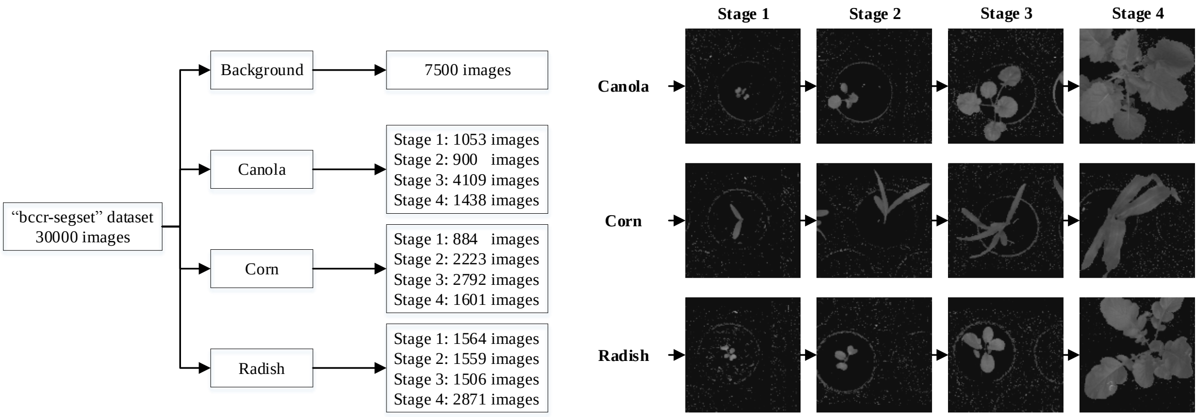

Figure 4: The “bccr-segset” dataset and its four-growth stages.

The average classification accuracies of the FLBP, FLBPCM and k-FLBPCM methods are listed in Table 7. Note that, in this investigation, the following typical values were selected: C=30, 60, 100 and γ=10^{-5}, 10^{-6}. The k-FLBPCM method again achieved the highest accuracies among all compared methods, confirming the results in the given "can-rad" dataset.

Table 7: Comparison of the average classification accuracies of the FLBP, FLBPCM and k-FLBPCM methods.

C	γ	Thickness	Method	Accuracy score
30	1E-05	2	k-FLBPCM, k=0.2	98.63%
60	1E-05	2	k-FLBPCM, k=0.2	98.61%
100	1E-06	2	k-FLBPCM, k=0.8	98.61%
30	1E-05	No thickness	FLBP	97.23%
60	1E-05	No thickness	FLBP	97.22%
100	1E-06	No thickness	FLBP	98.17%
30	1E-05	2	FLBPCM	97.04%
60	1E-05	2	FLBPCM	97.14%
100	1E-06	2	FLBPCM	96.01%

In order to find optimal (C, γ) pairs, we investigated the following parameter ranges: C = 1, 10, 30, 60, 100, 1000, γ = $10^{-5}, 10^{-6}$, k = 0.1, 0.2, 0.5, 0.8, 1 and thickness of 2. Only the 10 highest classification accuracies of the k-FLBPCM method were listed in Table 8. This method attained the highest classification accuracy of 98.63% with C =30, γ =10^{-5} and coefficient k=0.2.

Table 8: Average classification accuracies of the k-FLBPCM method for different C and γ parameters and coefficients k.

C	γ	Thickness	k-FLBPCM Method	Accuracy score
30	1E-05	2	k=0.2	98.63%
100	1E-06	2	k=0.8	98.61%
100	1E-05	2	k=0.2	98.61%
60	1E-05	2	k=0.2	98.61%
100	1E-06	2	k=1	98.60%
60	1E-06	2	k=0.8	98.58%
60	1E-06	2	k=1	98.57%
1000	1E-06	2	k=0.5	98.56%
30	1E-06	2	k=1	98.56%

1000	1E-06	2	k=1	98.51%

The k-FLBPCM method can classify plant images with different conditions, as shown in our two datasets, and improve the classification accuracies achieved previously [43]. Particularly, there is a significant improvement in performance when combining LBP features with a contour based mask. The average classification accuracies of the k-FLBPCM method have increased over the previously described method by up to 6.78% [45].

The F1-score results for each class are indicated in Table 9. Particularly, the F1 scores of the k-FLBPCM method significantly increased to 97.40% and 97.40% for canola and radish, from 84.41% and 83.43% respectively, which had used combined LBP operators in the previously published paper [45]. In addition, the testing time (millisecond/image) of the k-FLBPCM method was faster than the combined LBP method [45].

Table 9: Comparison of performance metrics between the k-FLBPCM and combined LBP methods for each class.

**Method**	**SVM kernel**	**Classes**	**Precision**	**Recall**	**F1-score**	**Testing time (ms/image)**
k-FLBPCM	RBF kernel	Background	100%	100%	100%	0.491
		Canola	96.80%	97.60%	97.40%	
		Corn	100%	100%	100%	
		Radish	97.60%	97.20%	97.40%	
Combined LBP operators LBP(8,1)+LBP (16,2)+LBP(24,3)	RBF kernel	Background	96.17%	98.87%	97.50%	1.419
		Canola	83.64%	85.20%	84.41%	
		Corn	98.64%	96.87%	97.75%	
		Radish	84.69%	82.27%	83.46%	

With the aim of reducing the misclassification, we investigated the misclassified images through visual inspection as shown in Figure 5. The first stage plants (Figure 5 (a), (b) and (c)), appear to have been misclassified due to the close morphological similarities. In addition, deformity of the leaves and stems, especially arising from perspective distortions (Figure 5 (e) (f)) and leaf diseases (Figure 5 (d)) can also lead to the identification errors. However, the k-FLBPCM method considerably reduced the number of

misclassified images and outperformed other methods by obtaining the high classification accuracy at 98.63%.

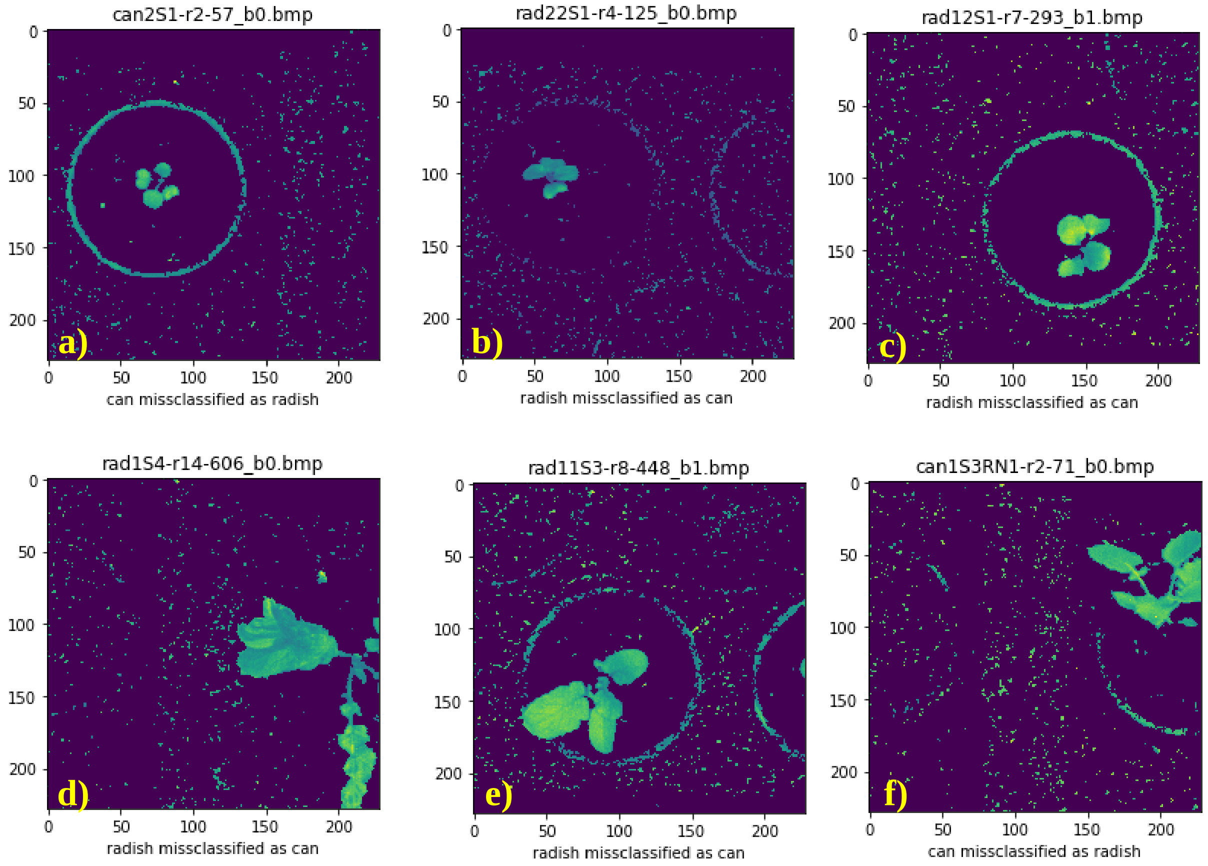

Figure 5: Misclassified images are printed from the model of the k-FLBPCM method with C =30, γ =10^{-5} and k=0.2

Conclusions

In this paper, the k-FLBPCM method combining LBP feature extraction with contour masks has been proposed for reducing the noise and improving the plant classification accuracy. Results have shown that various factors can reduce the weed identification accuracy, including outdoor scene complexity and morphological variability of plants. Based on the experimental results, the k-FLBPCM method had the best performance of 98.63% accuracy in identifiying similar morphological plants. This method is particularly useful to discriminate between two classes with highly similar morphologies, while

tolerating morphological variability within each class. Further, results have shown that the execution time of the proposed method is faster than the combined LBP method in the previous published paper. As a result, the proposed method helps to improve the plant classification with similar morphological features. Furthermore, the fast processing time of this method enhances the ability to implement the plant detection in the real time.

Future research might consider the potential of the k-FLBPCM method in diverse applications in order to identify objects of similar morphologies. Morphological cell analysis plays an significant role in supporting pathologists to accurately detect cancer cells [71, 72]. The advantages of the k-FLBPCM method is that image data can be reused for extracting morphological features and identifying abnormal cells.

Availability of supporting source code and requirements

Project name: k-FLBPCM-method

Project home page: https://github.com/vinguyenle/k-FLBPCM-method

Operating system: The code of k-FLBPCM method was written on Linux.

Programming language: Python 2.7.13

License: GNU General Public License v3.0

RRID:SCR_017973

Availability of supporting data and materials

All data are available at the provided links.

Bccr-segset dataset: https://data.pawsey.org.au/download/Weedvision/public/LBP-SVM-analysis/bccr-set/bccr-segset%20dataset.rar

Can-rad dataset: https://data.pawsey.org.au/download/Weedvision/public/LBP-SVM-analysis/bccr-set/can-rad_dataset.rar. Snapshots of our code and other supporting data can be found in the GigaScience repository, GigaDB [73].

Competing interests

The authors declare that they have no competing interests.

Funding

The work was supported by the Grains Research and Development Corporation (GRDC), Australian Research Council and Photonic Detection Systems Pty. Ltd, Australia (grant number WCA00004).

Authors' contributions

VNTL and BA proposed and designed the study. VNTL and BA constructed the datasets, wrote and optimized the code. VNTL, SA and KA wrote, revised the manuscript, proofread and improved the manuscript.

Acknowledgments

The authors would like to thank the Pawsey Supercomputing Centre for computation resources and storage of the public "bccr-segset" dataset.

References

1. Slaughter D, Giles D and Downey D. Autonomous robotic weed control systems: A review. Computers and electronics in agriculture. 2008;61 1:63-78.
2. Yu Q, Zhang XQ, Hashem A, Walsh MJ and Powles SB. ALS gene proline (197) mutations confer ALS herbicide resistance in eight separated wild radish (Raphanus raphanistrum) populations. Weed Science. 2003;51 6:831-8.
3. Walsh MJ, Powles SB, Beard BR, Parkin BT and Porter SA. Multiple-herbicide resistance across four modes of action in wild radish (Raphanus raphanistrum). Weed science. 2004;52 1:8-13.
4. Blackshaw RE, Lemerle D, Mailer R and Young KR. Influence of wild radish on yield and quality of canola. Weed Science. 2002;50 3:344-9.
5. Robertson M, Whish J and Smith F. Simulating competition between canola and wild radish. 12th Australian research assembly on Brassicas. 2001:2-5.
6. Hansen P and Schjoerring J. Reflectance measurement of canopy biomass and nitrogen status in wheat crops using normalized difference vegetation indices and partial least squares regression. Remote sensing of environment. 2003;86 4:542-53.
7. Thorp K and Tian L. A review on remote sensing of weeds in agriculture. Precision Agriculture. 2004;5 5:477-508.
8. Huete A, Didan K, Miura T, Rodriguez EP, Gao X and Ferreira LG. Overview of the radiometric and biophysical performance of the MODIS vegetation indices. Remote sensing of environment. 2002;83 1:195-213.
9. Ozdogan M, Yang Y, Allez G and Cervantes C. Remote sensing of irrigated agriculture: Opportunities and challenges. Remote sensing. 2010;2 9:2274-304.
10. Lass LW, Prather TS, Glenn NF, Weber KT, Mundt JT and Pettingill J. A review of remote sensing of invasive weeds and example of the early detection of spotted knapweed (Centaurea maculosa) and babysbreath (Gypsophila paniculata) with a hyperspectral sensor. Weed Science. 2005;53 2:242-51.

11. Pietikäinen M, Hadid A, Zhao G and Ahonen T. Computer vision using local binary patterns. Springer Science & Business Media; 2011.

12. Guo Z, Zhang L and Zhang D. A completed modeling of local binary pattern operator for texture classification. IEEE Transactions on Image Processing. 2010;19 6:1657-63.

13. Heikkilä M, Pietikäinen M and Schmid C. Description of interest regions with local binary patterns. Pattern recognition. 2009;42 3:425-36.

14. Ojala T, Pietikainen M and Maenpaa T. Multiresolution gray-scale and rotation invariant texture classification with local binary patterns. IEEE Transactions on pattern analysis and machine intelligence. 2002;24 7:971-87.

15. Happy S, George A and Routray A. A real time facial expression classification system using Local Binary Patterns. In: *Intelligent Human Computer Interaction (IHCI), 4th International Conference* 2012, pp.1-5. IEEE.

16. Lahdenoja O, Poikonen J and Laiho M. Towards understanding the formation of uniform local binary patterns. ISRN Machine Vision. 2013.

17. Heikkila M and Pietikainen M. A texture-based method for modeling the background and detecting moving objects. IEEE transactions on pattern analysis and machine intelligence. 2006;28 4:657-62.

18. Kellokumpu V, Zhao G and Pietikäinen M. Human activity recognition using a dynamic texture based method. In: *BMVC* 2008, p.2.

19. Liao S, Law MW and Chung AC. Dominant local binary patterns for texture classification. IEEE transactions on image processing. 2009;18 5:1107-18.

20. Ahonen T, Hadid A and Pietikainen M. Face description with local binary patterns: Application to face recognition. IEEE transactions on pattern analysis and machine intelligence. 2006;28 12:2037-41.

21. Jin H, Liu Q, Lu H and Tong X. Face detection using improved LBP under Bayesian framework. In: *Image and Graphics (ICIG'04), Third International Conference* 2004, pp.306-9. IEEE.

22. Louis W and Plataniotis KN. Co-occurrence of local binary patterns features for frontal face detection in surveillance applications. EURASIP Journal on Image and Video Processing. 2010;2011 1:745487.

23. Zhao G and Pietikainen M. Dynamic texture recognition using local binary patterns with an application to facial expressions. IEEE transactions on pattern analysis and machine intelligence. 2007;29 6.

24. Shan C, Gong S and McOwan PW. Facial expression recognition based on local binary patterns: A comprehensive study. Image and Vision Computing. 2009;27 6:803-16.

25. Nanni L and Lumini A. Local binary patterns for a hybrid fingerprint matcher. Pattern recognition. 2008;41 11:3461-6.

26. Takala V, Ahonen T and Pietikäinen M. Block-based methods for image retrieval using local binary patterns. In: *Scandinavian Conference on Image Analysis* 2005, pp.882-91. Springer.

27. Subrahmanyam M, Maheshwari R and Balasubramanian R. Local maximum edge binary patterns: a new descriptor for image retrieval and object tracking. Signal Processing. 2012;92 6:1467-79.

28. Liu L, Zhao L, Long Y, Kuang G and Fieguth P. Extended local binary patterns for texture classification. Image and Vision Computing. 2012;30 2:86-99.

29. Zhou H, Wang R and Wang C. A novel extended local-binary-pattern operator for texture analysis. Information Sciences. 2008;178 22:4314-25.

30. Guo Y, Zhao G and PietikäInen M. Discriminative features for texture description. Pattern Recognition. 2012;45 10:3834-43.

31. Qi X, Xiao R, Li C-G, Qiao Y, Guo J and Tang X. Pairwise rotation invariant co-occurrence local binary pattern. IEEE transactions on pattern analysis and machine intelligence. 2014;36 11:2199-213.

32. Iakovidis DK, Keramidas EG and Maroulis D. Fuzzy local binary patterns for ultrasound texture characterization. In: *International conference image analysis and recognition* 2008, pp.750-9. Springer.

33. Chen J, Kellokumpu V, Zhao G and Pietikäinen M. RLBP: Robust Local Binary Pattern. In: *BMVC* 2013.

34. Fathi A and Naghsh-Nilchi AR. Noise tolerant local binary pattern operator for efficient texture analysis. Pattern Recognition Letters. 2012;33 9:1093-100.

35. Ren J, Jiang X and Yuan J. Noise-resistant local binary pattern with an embedded error-correction mechanism. IEEE Transactions on Image Processing. 2013;22 10:4049-60.

36. Liu L, Lao S, Fieguth PW, Guo Y, Wang X and Pietikäinen M. Median robust extended local binary pattern for texture classification. IEEE Transactions on Image Processing. 2016;25 3:1368-81.

37. Ahmed F, Bari AH, Shihavuddin A, Al-Mamun HA and Kwan P. A study on local binary pattern for automated weed classification using template matching and support vector machine. In: *2011 IEEE 12th International Symposium on Computational Intelligence and Informatics (CINTI)* 2011, pp.329-34. IEEE.

38. Ahmed F, Kabir MH, Bhuyan S, Bari H and Hossain E. Automated weed classification with local pattern-based texture descriptors. International Arab Journal of Information Technology. 2014;11 1:87-94.

39. Lottes P, Hörferlin M, Sander S and Stachniss C. Effective Vision‐ based Classification for Separating Sugar Beets and Weeds for Precision Farming. Journal of Field Robotics. 2017;34 6:1160-78.

40. McCool C, Sa I, Dayoub F, Lehnert C, Perez T and Upcroft B. Visual detection of occluded crop: For automated harvesting. In: *2016 IEEE International Conference on Robotics and Automation (ICRA)* 2016, pp.2506-12. IEEE.

41. Dubey SR and Jalal AS. Detection and classification of apple fruit diseases using complete local binary patterns. In: *2012 Third International Conference on Computer and Communication Technology* 2012, pp.346-51. IEEE.

42. Herdiyeni Y and Santoni MM. Combination of morphological, local binary pattern variance and color moments features for indonesian medicinal plants identification. In: *2012 International Conference on Advanced Computer Science and Information Systems (ICACSIS)* 2012, pp.255-9. IEEE.

43. Le VNT, Apopei B and Alameh K. Effective plant discrimination based on the combination of local binary pattern operators and multiclass support vector machine methods. Information Processing in Agriculture. 2018.

44. Savelonas MA, Iakovidis DK and Maroulis D. LBP-guided active contours. Pattern Recognition Letters. 2008;29 9:1404-15.

45. Satpathy A, Jiang X and Eng H-L. LBP-based edge-texture features for object recognition. IEEE Transactions on Image Processing. 2014;23 5:1953-64.

46. Nguyen DT, Ogunbona PO and Li W. A novel shape-based non-redundant local binary pattern descriptor for object detection. Pattern recognition. 2013;46 5:1485-500.

47. Lin J and Chiu C-T. Low-complexity face recognition using contour-based binary descriptor. IET Image Processing. 2017;11 12:1179-87.

48. Scholkopf B and Smola AJ. Learning with kernels: support vector machines, regularization, optimization, and beyond. MIT press; 2001.

49. Ma Y and Guo G. Support vector machines applications. Springer; 2014.

50. Bai MR, Krishna VV and SreeDevi J. A new morphological approach for noise removal cum edge detection. International Journal of Computer Science Issues. 2010;7 6:187.

51. Serra J. Image analysis and mathematical morphology. Academic Press, Inc.; 1983.

52. Haralick RM, Sternberg SR and Zhuang X. Image analysis using mathematical morphology. IEEE Transactions on pattern analysis and machine intelligence. 1987; 4:532-50.

53. Yu-Qian Z, Wei-Hua G, Zhen-Cheng C, Jing-Tian T and Ling-Yun L. Medical images edge detection based on mathematical morphology. In: *2005 IEEE engineering in medicine and biology 27th annual conference* 2006, pp.6492-5. IEEE.

54. Vincent L. Morphological area openings and closings for grey-scale images. Shape in Picture. Springer; 1994. p. 197-208.

55. Ojala T, Pietikäinen M and Harwood D. A comparative study of texture measures with classification based on featured distributions. Pattern recognition. 1996;29 1:51-9.

56. Pietikäinen M, Ojala T and Xu Z. Rotation-invariant texture classification using feature distributions. Pattern Recognition. 2000;33 1:43-52.

57. Guo G, Li SZ and Chan KL. Support vector machines for face recognition. Image and Vision Computing. 2001;19 9-10:631-8.

58. Phillips PJ. Support vector machines applied to face recognition. In: *Advances in Neural Information Processing Systems* 1999, pp.803-9.

59. Tellaeche A, Pajares G, Burgos-Artizzu XP and Ribeiro A. A computer vision approach for weeds identification through Support Vector Machines. Applied Soft Computing. 2011;11 1:908-15.

60. Guerrero JM, Pajares G, Montalvo M, Romeo J and Guijarro M. Support vector machines for crop/weeds identification in maize fields. Expert Systems with Applications. 2012;39 12:11149-55.

61. Rumpf T, Mahlein A-K, Steiner U, Oerke E-C, Dehne H-W and Plümer L. Early detection and classification of plant diseases with support vector machines based on hyperspectral reflectance. Computers and electronics in agriculture. 2010;74 1:91-9.

62. Patil JK, Kumar R and Research. Advances in image processing for detection of plant diseases. Journal of Advanced Bioinformatics Applications. 2011;2 2:135-41.

63. Wu L and Wen Y. Weed/corn seedling recognition by support vector machine using texture features. African Journal of Agricultural Research. 2009;4 9:840-6.

64. Ahmed F, Al-Mamun HA, Bari AH, Hossain E and Kwan P. Classification of crops and weeds from digital images: A support vector machine approach. Crop Protection. 2012;40:98-104.

65. Boser BE, Guyon IM and Vapnik VN. A training algorithm for optimal margin classifiers. In: *Proceedings of the fifth annual workshop on Computational learning theory* 1992, pp.144-52. ACM.

66. Cortes C and Vapnik V. Support-vector networks. Machine learning. 1995;20 3:273-97.

67. Chang C-C, Lin C-J and technology. LIBSVM: a library for support vector machines. ACM transactions on intelligent systems. 2011;2 3:27.

68. Gunn SR. Support vector machines for classification and regression. ISIS technical report. 1998;14 1:5-16.

69. Radford AE. Vascular plant systematics. 1974.

70. Hsu C-W, Chang C-C and Lin C-J. A practical guide to support vector classification. Taipei, 2003.

71. Kalinin AA, Allyn-Feuer A, Ade A, Fon G-V, Meixner W, Dilworth D, et al. 3D shape modeling for cell nuclear morphological analysis and classification. Scientific reports. 2018;8 1:1-14.

72. Chen S, Zhao M, Wu G, Yao C and Zhang J. Recent advances in morphological cell image analysis. Computational and mathematical methods in medicine. 2012;2012.

73. Le VNT; Ahderom S; Apopei B; Alameh K: Supporting data for "A novel k-FLBPCM method for detecting morphologically similar crops and weeds based on the combination of contour masks and Local Binary Pattern operators" *GigaScience* Database. 2020. http://dx.doi.org/10.5524/100708

Click here to access/download;Table;Table 1.JPG

Bin values of different methods	Bin 1	Bin 2	Bin 3	Bin 4	Bin 5	Bin 6	Bin 7	Bin 8	Bin 10
FLBP	1212	913	355	727	680	1351	305	402	974
FLBPbCM	122	121	96	259	275	143	45	33	139
k-FLBPCM with k=0.5	728	577.5	273.5	622.5	615	818.5	197.5	234	626

Click here to access/download;Table;Table 2.JPG

C	γ	Thickness	Methods	Accuracy Score	
				Polynomial kernel of degree 2	RBF kernel
10	1E-05	2	k-FLBPCM method, k=0.5	95.46%	97.32%
10	1E-05	2	k-FLBPCM method, k=0.2	94.91%	97.32%
10	1E-05	2	k-FLBPCM method, k=0.1	94.27%	96.40%
60	1E-06	2	k-FLBPCM method, k=1	94.92%	97.50%
60	1E-06	2	k-FLBPCM method, k=0.5	94.56%	96.89%
60	1E-06	2	k-FLBPCM method, k=0.2	93.55%	96.06%
10	1E-05	No thickness	FLBP method	93.53%	95.36%
60	1E-06	No thickness	FLBP method	93.74%	96.72%
10	1E-05	2	FLBPCM method	88.53%	94.07%
60	1E-06	2	FLBPCM method	88.26%	94.83%

Click here to access/download;Table;Table 3.JPG

C	γ	Thickness	k-FLBPCM method	Accuracy score
30	1E-05	2	k=0.2	97.50%
60	1E-06	2	k=1	97.50%
60	1E-05	2	k=0.2	97.49%
100	1E-05	2	k=0.2	97.45%
100	1E-06	2	k=1	97.42%
30	1E-06	2	k=1	97.42%
100	1E-06	2	k=0.7	97.40%
30	1E-05	2	k=0.5	97.37%
100	1E-06	2	k=0.8	97.35%
60	1E-06	2	k=0.8	97.34%

Click here to access/download;Table;Table 4.JPG

C	γ	Classification Accuracy of the FLBP method
**100**	**1E-06**	**95.13%**
1000	1E-06	95.03%
60	1E-06	94.96%
30	1E-06	94.92%
10	1E-06	94.31%
1000	1E-07	93.92%
10	1E-05	93.78%
30	1E-05	93.67%
60	1E-05	93.62%
100	1E-05	93.61%

Click here to access/download;Table;Table 5.JPG

C	γ	Thickness	Classification Accuracy of the FLBPbCM method
**30**	**1E-05**	**8**	**93.95%**
**30**	**1E-05**	7	**93.95%**
100	1E-05	2	93.94%
30	1E-05	6	93.88%
30	1E-05	5	93.88%
10	1E-05	8	93.88%
10	1E-05	7	93.88%
1000	1E-05	2	93.87%
60	1E-05	6	93.87%
100	1E-05	6	93.81%

Click here to access/download;Table;Table 6.JPG

C	γ	Thickness	k-FLBPCM method	Classification Accuracy
**1000**	**1E-06**	**2**	**k=0.5**	**96.21%**
30	1E-05	2	k=0.5	96.19%
10	1E-05	2	k=0.5	96.18%
30	1E-05	4	k=0.5	96.16%
30	1E-05	3	k=0.5	96.16%
60	1E-05	2	k=0.5	96.15%
10	1E-05	4	k=0.5	96.14%
10	1E-05	3	k=0.5	96.14%
30	1E-05	2	k=0.2	96.13%
30	1E-05	4	k=0.2	96.11%

Click here to access/download;Table;Table 7.JPG

C	γ	Thickness	Method	Accuracy score
30	1E-05	2	k-FLBPCM, k=0.2	98.63%
60	1E-05	2	k-FLBPCM, k=0.2	98.61%
100	1E-06	2	k-FLBPCM, k=0.8	98.61%
30	1E-05	No thickness	FLBP	97.23%
60	1E-05	No thickness	FLBP	97.22%
100	1E-06	No thickness	FLBP	98.17%
30	1E-05	2	FLBPCM	97.04%
60	1E-05	2	FLBPCM	97.14%
100	1E-06	2	FLBPCM	96.01%

Click here to access/download;Table;Table 8.JPG

C	γ	Thickness	k-FLBPCM Method	Accuracy score
30	1E-05	2	k=0.2	98.63%
100	1E-06	2	k=0.8	98.61%
100	1E-05	2	k=0.2	98.61%
60	1E-05	2	k=0.2	98.61%
100	1E-06	2	k=1	98.60%
60	1E-06	2	k=0.8	98.58%
60	1E-06	2	k=1	98.57%
1000	1E-06	2	k=0.5	98.56%
30	1E-06	2	k=1	98.56%
1000	1E-06	2	k=1	98.51%

Click here to access/download;Table;Table 9.JPG

<table>
<tr><th>Method</th><th>SVM kernel</th><th>Classes</th><th>Precision</th><th>Recall</th><th>F1-score</th></tr>
<tr><td rowspan="4">k-FLBPCM</td><td rowspan="4">RBF kernel</td><td>Background</td><td>100%</td><td>100%</td><td>100%</td></tr>
<tr><td>Canola</td><td>96.80%</td><td>97.60%</td><td>97.40%</td></tr>
<tr><td>Corn</td><td>100%</td><td>100%</td><td>100%</td></tr>
<tr><td>Radish</td><td>97.60%</td><td>97.20%</td><td>97.40%</td></tr>
<tr><td>Combined LBP operators</td><td rowspan="4">RBF kernel</td><td>Background</td><td>96.17%</td><td>98.87%</td><td>97.50%</td></tr>
<tr><td rowspan="3">LBP(8,1)+LBP(16,2)+LBP(24,3)</td><td>Canola</td><td>83.64%</td><td>85.20%</td><td>84.41%</td></tr>
<tr><td>Corn</td><td>98.64%</td><td>96.87%</td><td>97.75%</td></tr>
<tr><td>Radish</td><td>84.69%</td><td>82.27%</td><td>83.46%</td></tr>
</table>

Click here to access/download;Figure;Figure 1.jpg

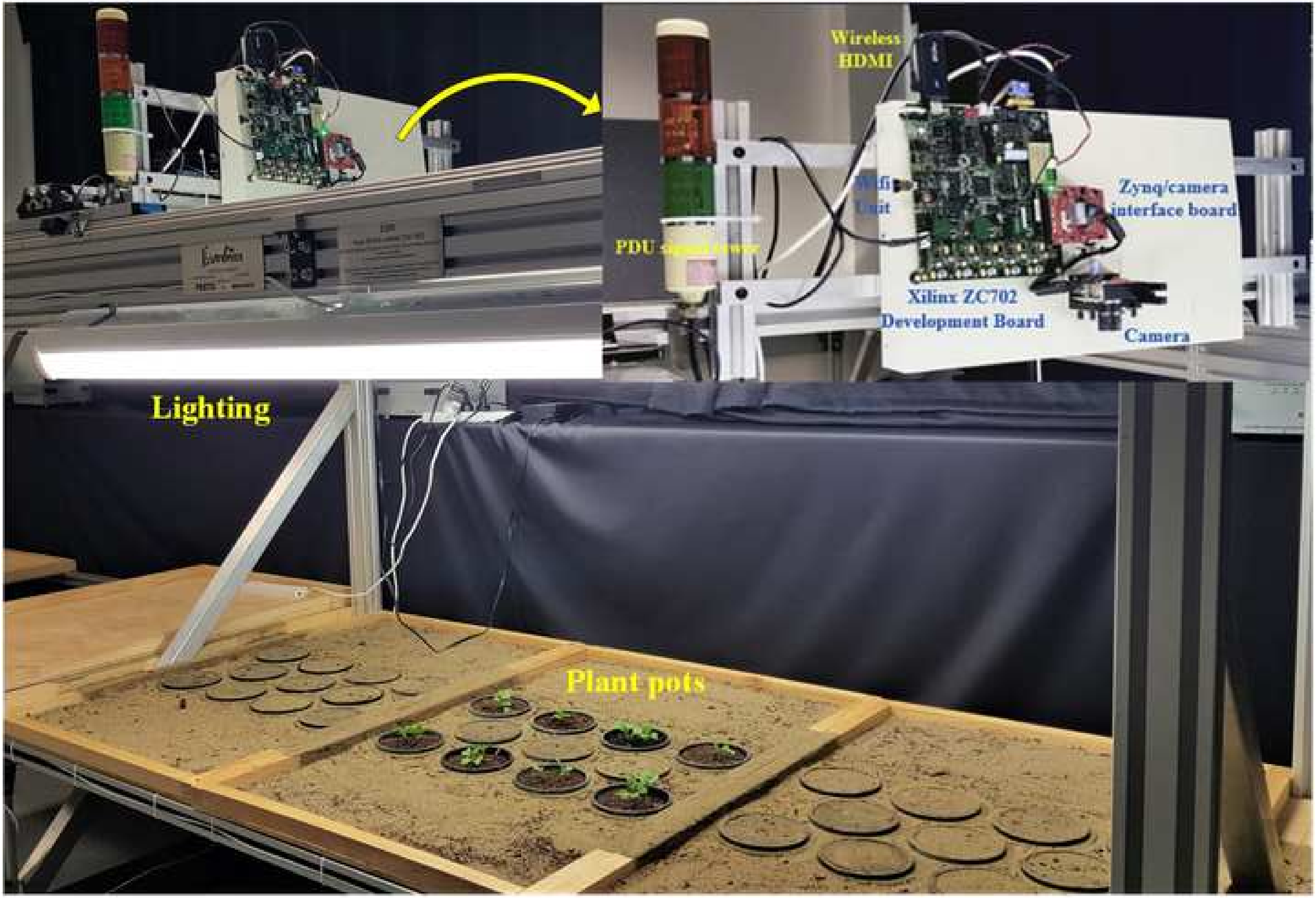

Click here to access/download;Figure;Figure 10.jpg

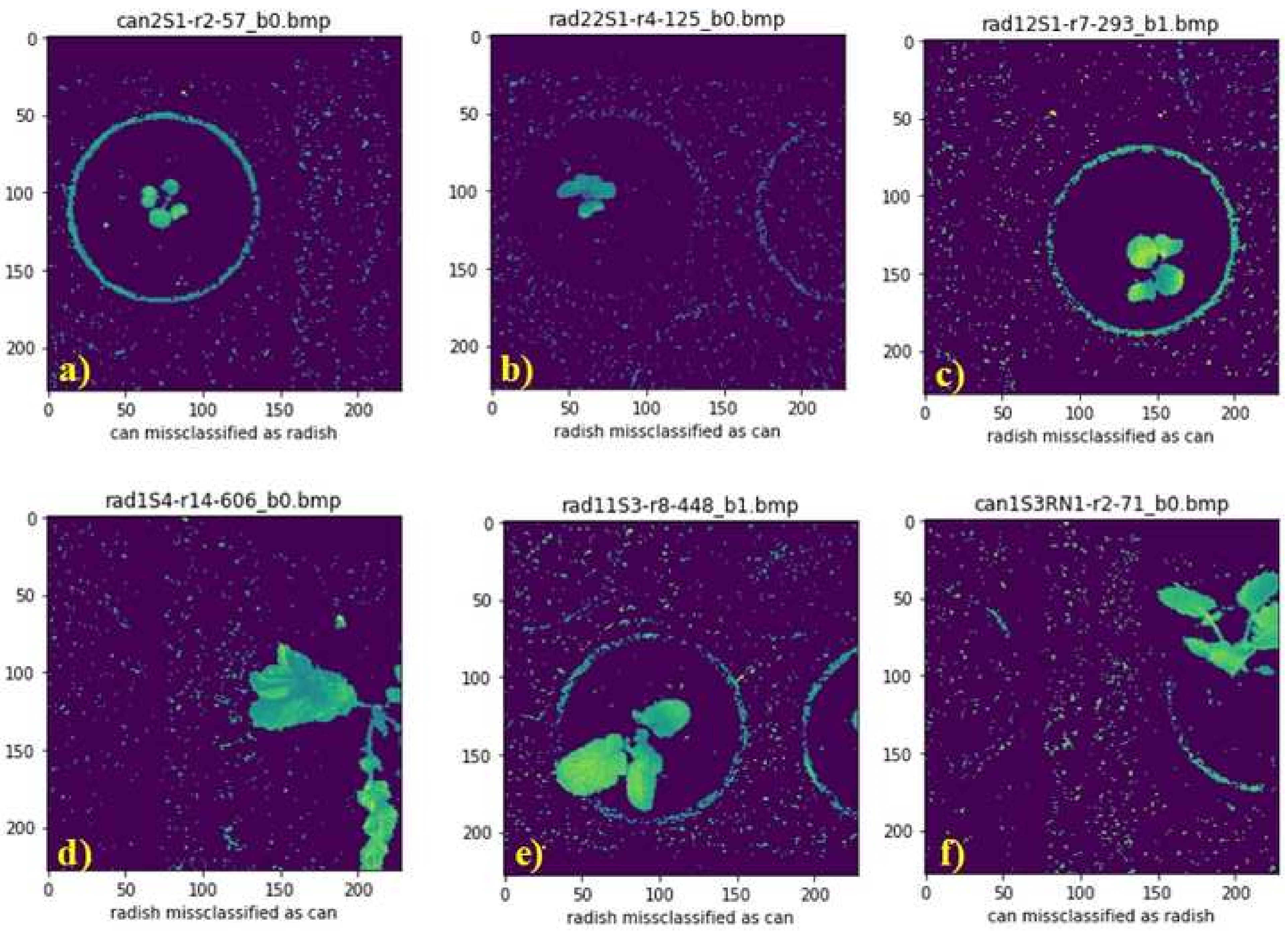

Click here to access/download;Figure;Figure 2.jpg

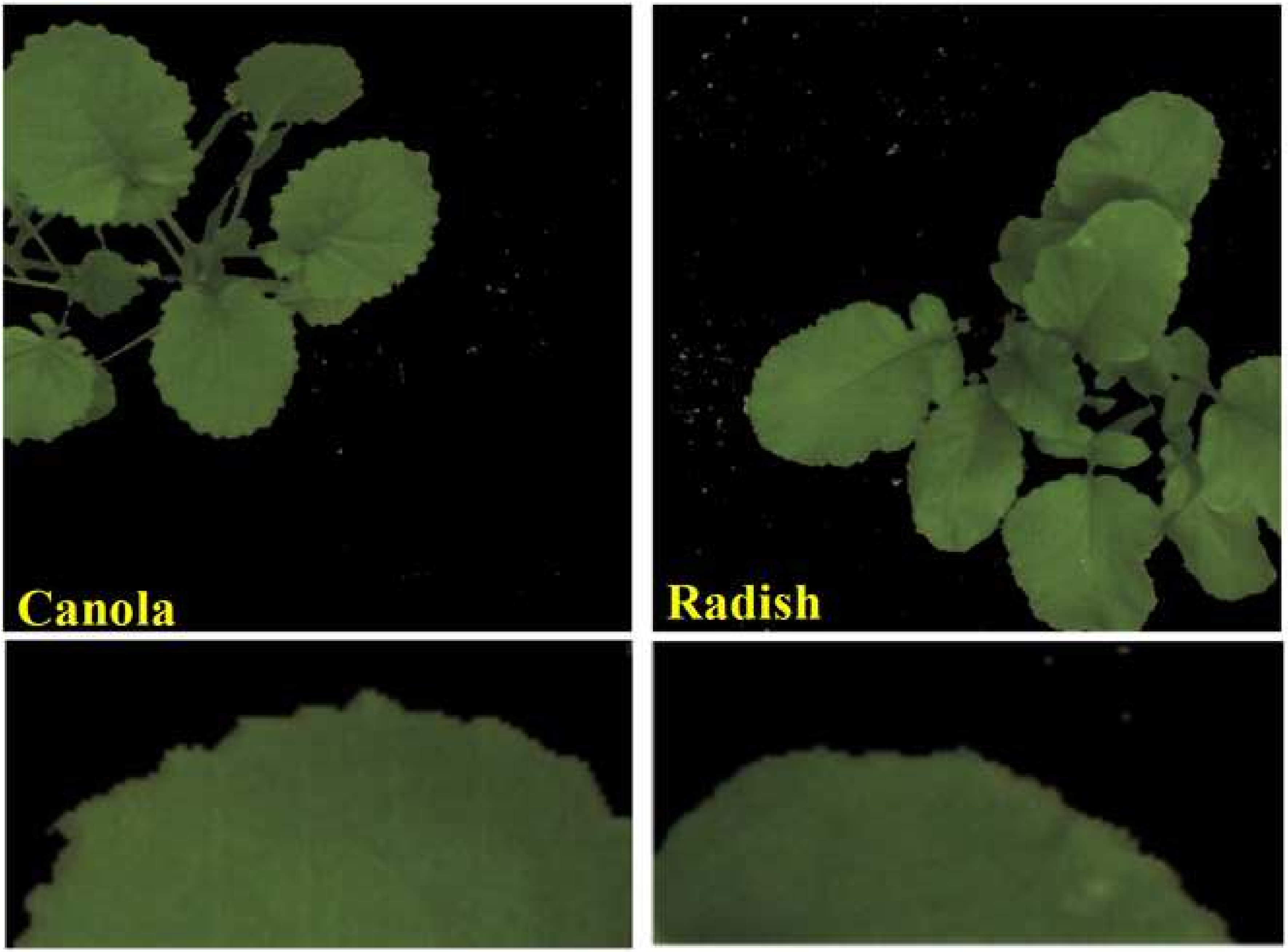

Click here to access/download;Figure;Figure 3.jpg

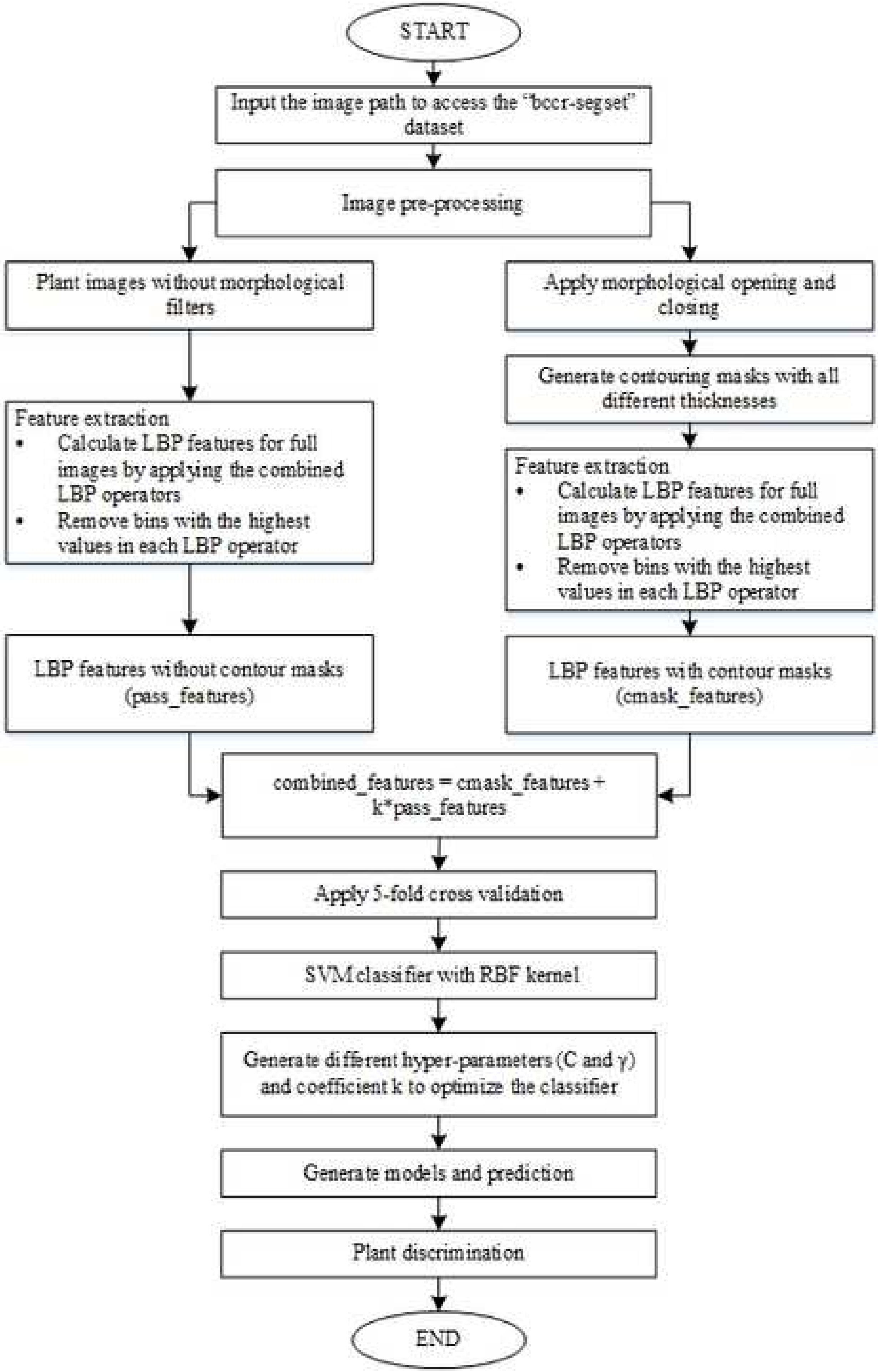

Click here to access/download;Figure;Figure 4.jpg

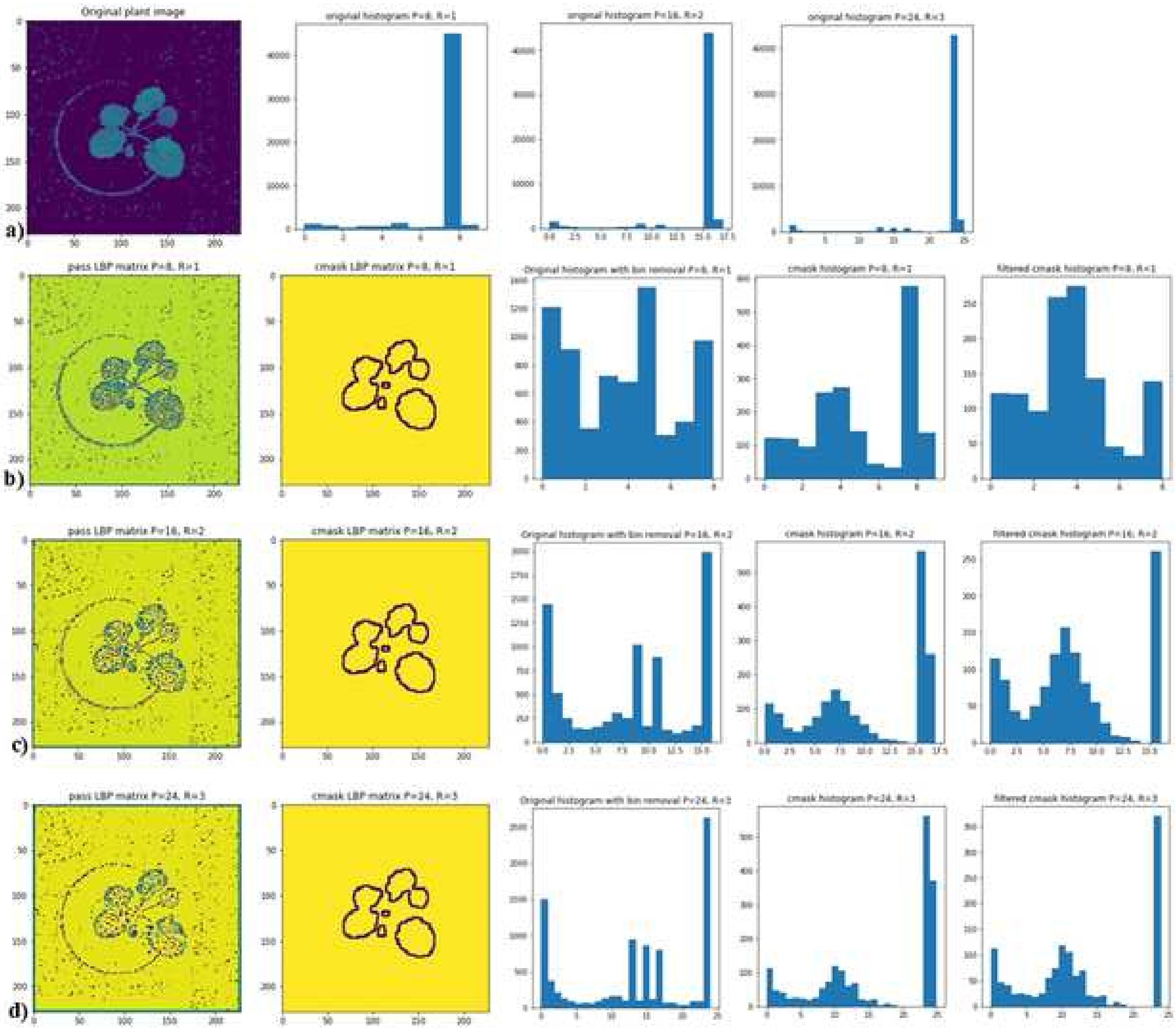

Click here to access/download;Figure;Figure 5.jpg

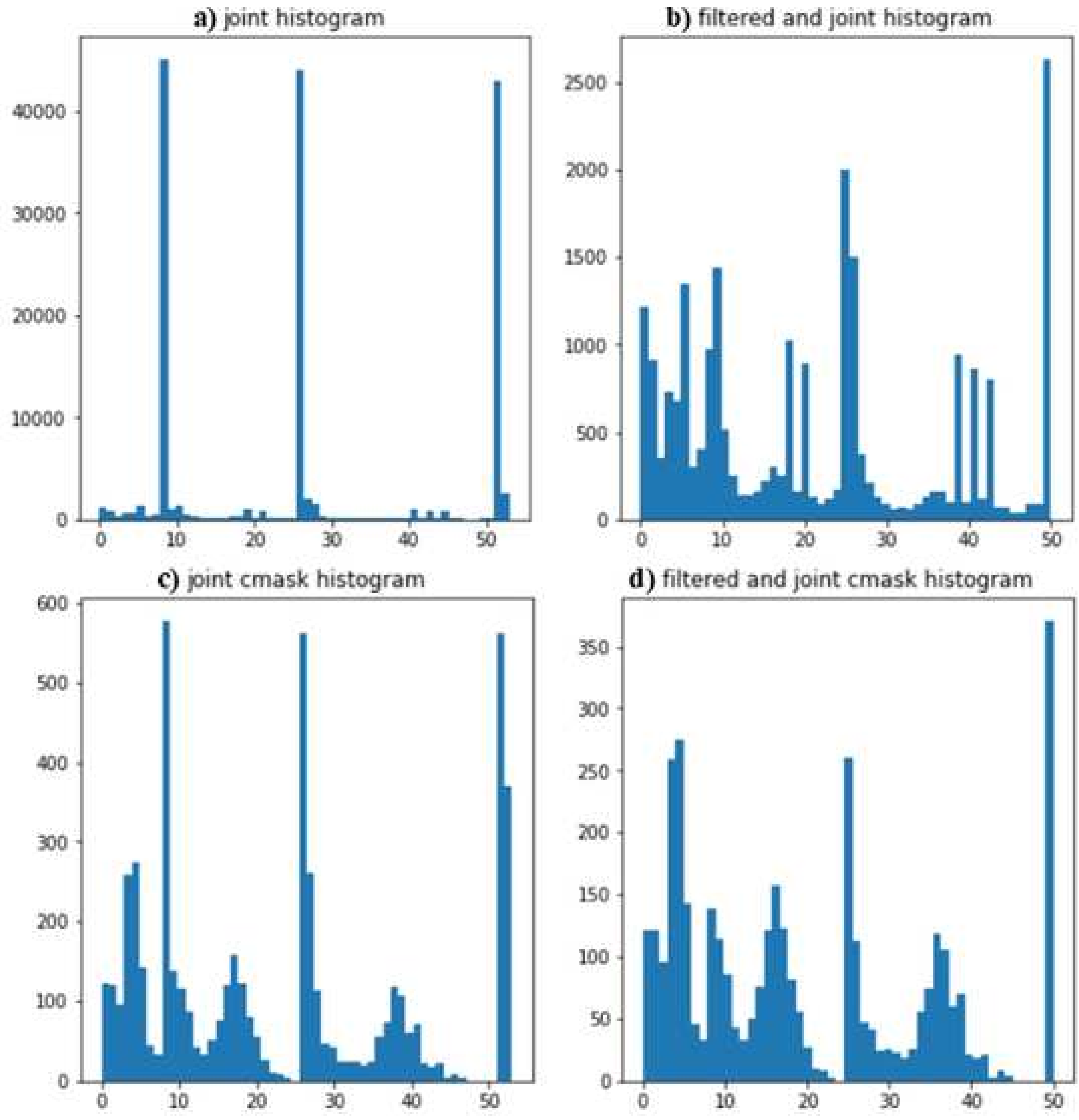

Click here to access/download;Figure;Figure 6.jpg

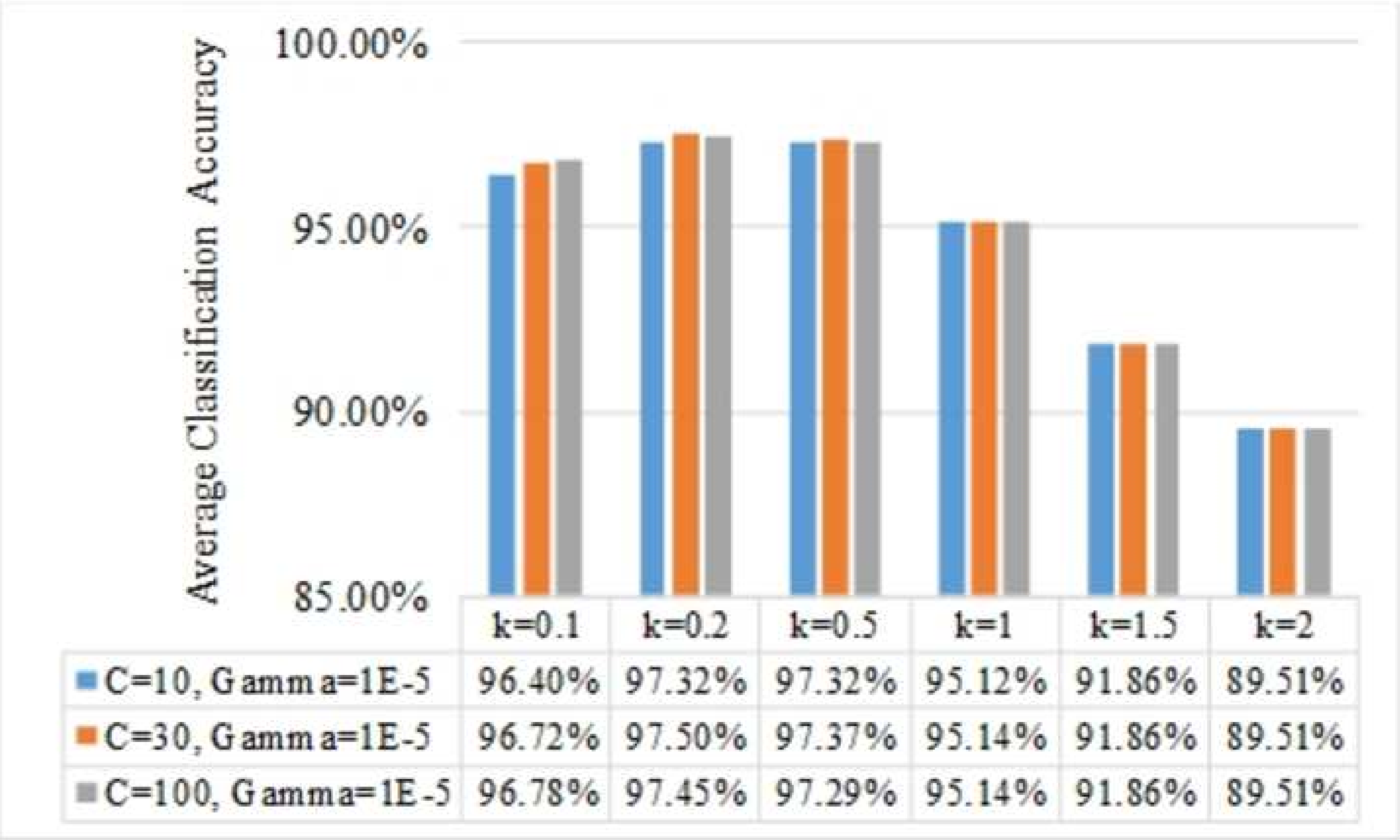

Click here to access/download;Figure;Figure 7.jpg

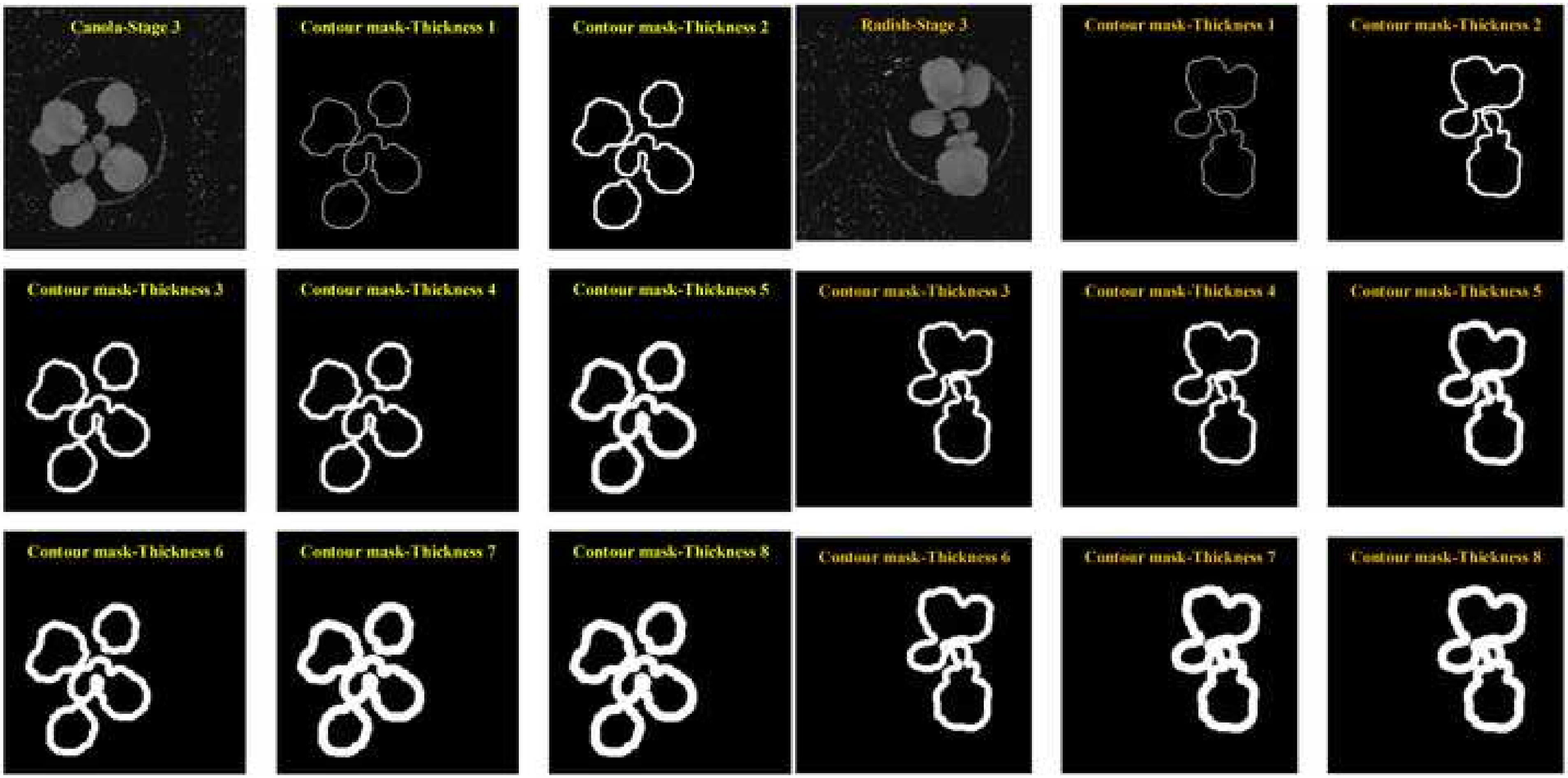

Click here to access/download;Figure;Figure 8.jpg

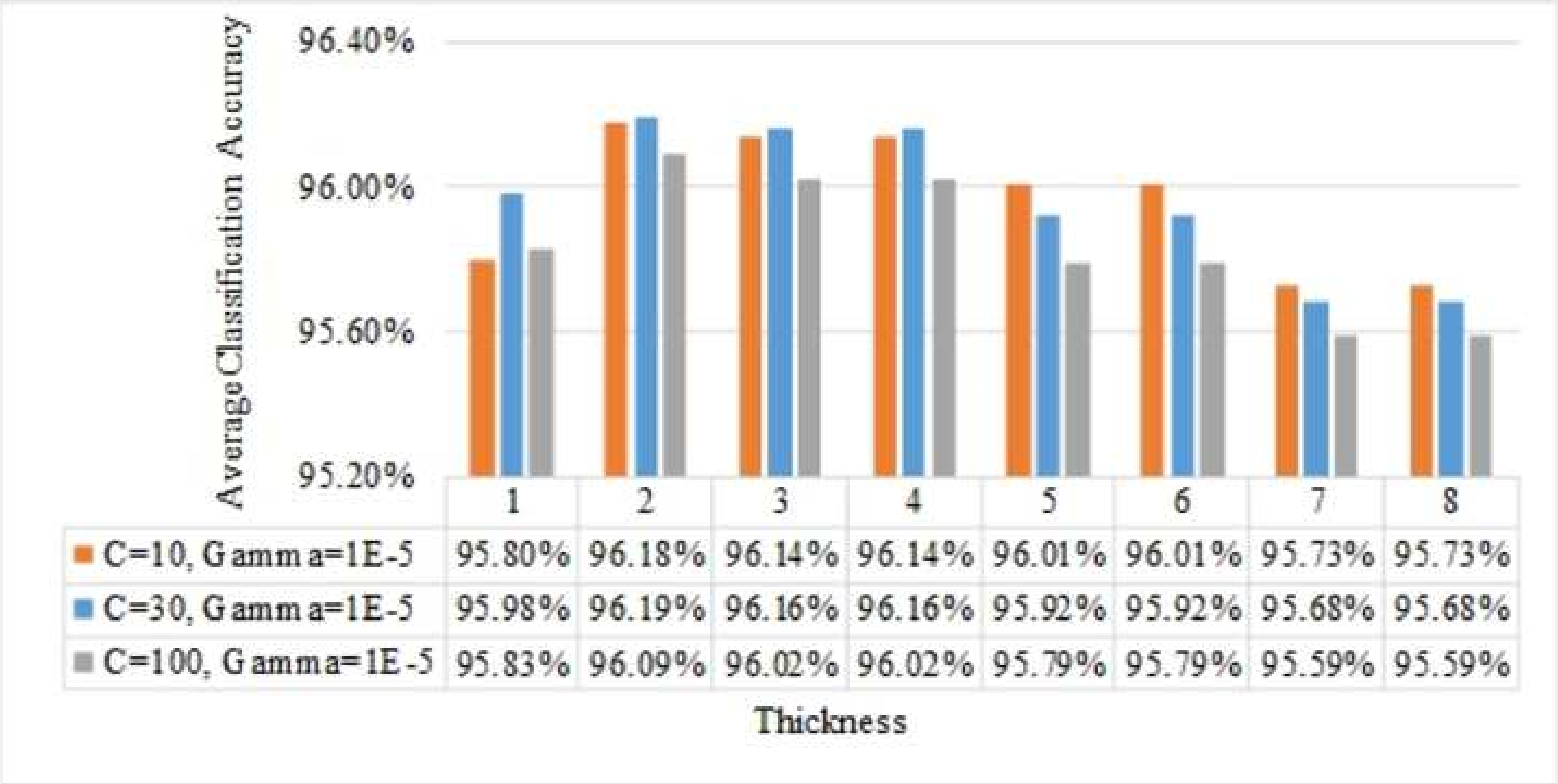

	1	2	3	4	5	6	7	8
C=10, Gamma=1E-5	95.80%	96.18%	96.14%	96.14%	96.01%	96.01%	95.73%	95.73%
C=30, Gamma=1E-5	95.98%	96.19%	96.16%	96.16%	95.92%	95.92%	95.68%	95.68%
C=100, Gamma=1E-5	95.83%	96.09%	96.02%	96.02%	95.79%	95.79%	95.59%	95.59%

Click here to access/download;Figure;Figure 9.jpg

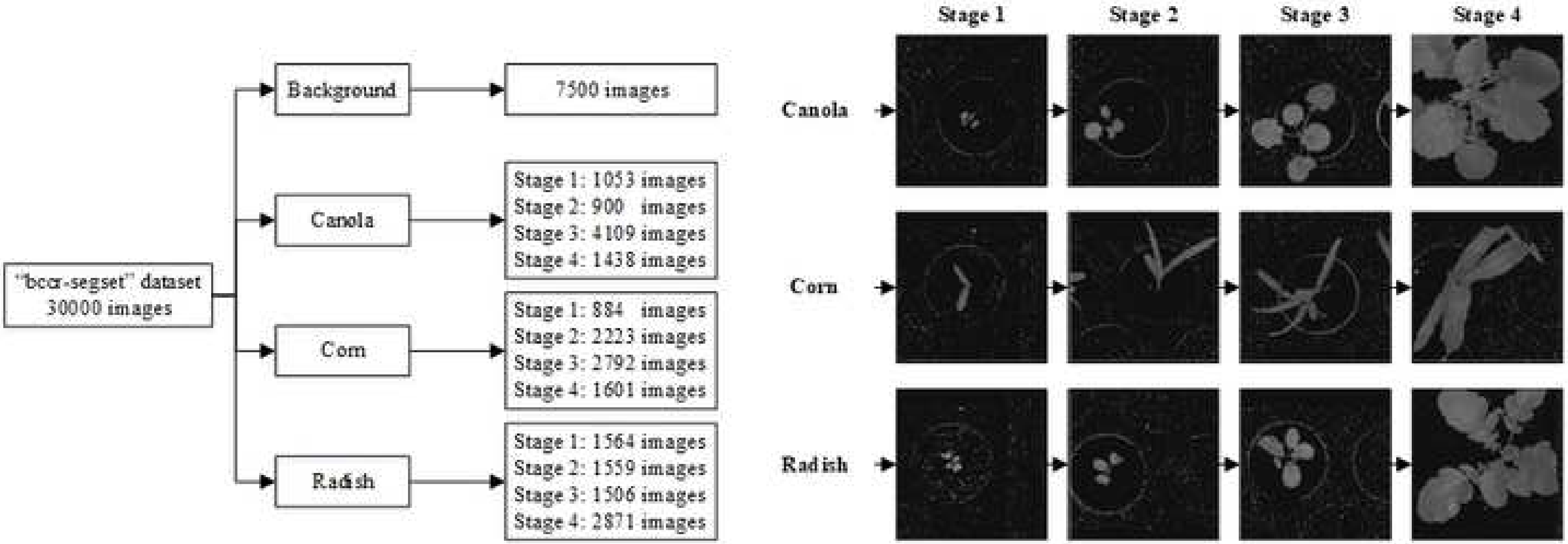

Click here to access/download
Supplementary Material
Declaration of Interest.pdf

Response to Reviewers
Click here to access/download;Personal Cover;Response to Reviewers_1.docx

RESPONSE TO REVIEWERS

We would like to thank the Reviewers for your valuable and detailed comments. We have gone through and seriously considered every comment. We would like to summarise our response to the Reviewers' comments as follows:

Editor's comment: Please register any new software application in the bio.tools and SciCrunch.org databases to receive RRID (Research Resource Identification Initiative ID) and biotoolsID identifiers, and include these in your manuscript. This will facilitate tracking, reproducibility and re-use of your tool.
Response: We have registered an RRID account for our algorithm on SciCrunch. The RRID for this resource is: k-FLBPCM-method, RRID: SCR_017973.

Reviewer 1's comments:
Comment 1: Do the authors consider the reuse potential of the k-FLBPCM method limited to classifying plant images? Alternatively, could this method be used in a Digital Pathology context to identify between, for example, epithelial and mesenchymal cells that have relatively similar morphology? A "reuse potential" section outlining the value of the k-FLBPCM method would be a useful addition to the manuscript for future researchers.
Response to Comment 1: We thank the Reviewer for your suggestion. We have now added a section in The Conclusion section from lines 500 to line 503 as follows: "Future research might consider the potential of the k-FLBPCM method in diverse applications in order to identify objects of similar morphologies. Morphological cell analysis plays a significant role in supporting pathologists to accurately detect cancer cells [1, 2]. The advantages of the k-FLBPCM method is that image data can be reused for extracting morphological features and identifying abnormal cells."

Comment 2: The GitHub archive (https://aus01.safelinks.protection.outlook.com/?url=https%3A%2F%2Fgithub.com%2Fvinguyenle%2Fk-FLBPCM-method&data=02%7C01%7Cvlenguye%40our.ecu.edu.au%7C69e0e0c2143a453e644508d79d7c7421%7C9bcb323d7fa345e7a36f6d9cfdbcc272%7C1%7C0%7C637151030165905119&sdata=tRM0OVS92c5jAbj7l9w4dAp5CrwuDR46EsL5rGuqf2o%3D&reserved=0) does not appear to have a license associated with it. Will the authors be ascribing an OSI-approved license to this GitHub archive?
Response to Comment 2: We thank the Reviewer for your advice. We have added a General Public License (GPL) to our project on Github (https://github.com/vinguyenle/k-FLBPCM-method).

Comment 3: To encourage reuse of the image data, I recommend that the bccr-segset dataset is archived in the GigaScience DataBase (GigaDB). Does GigaScience have permission to archive the bccr-segset image dataset?
Comment 4: To encourage reuse of the image data, I recommend that the can-rad dataset is archived in the GigaScience DataBase (GigaDB). Does GigaScience have permission to archive the can-rad image dataset?
Response to Comments 3 and 4: Since the data has already been an open-source dataset, you can archive the bccr-segset and can-rad image dataset in the GigaScience DataBase.

Reviewer 2's comments:

Comment 1: The methods used for investigation which includes LBP variants, Morphological processing and SVM Classifier are well described.

Comment 2: Conclusions are adequately supported by the experimental results.

Comment 4: Minor language editing may be possible. It does not require a heavy editing for language and clarity.

Response to Comments 1, 2 and 4: We thank the Reviewer for your comments.

Comment 3: Quality of language in the manuscript is good. However grammatical corrections are needed at some lines as below: Line 97: correct sentence grammatically. Line 246: 'However, there is some differences between': it may be: However, 'there are some differences between'.

Response to Comment 3: We thank the Reviewer. We have corrected all grammatical errors, as requested.

Comment 5: Reason behind selecting following statistical parameters should be mentioned: C =1, 10, 30, 60, 100, 1000, γ =10^−4, 10^−5, 10^−6, 10^−7and k =0.1, 0.2, 0.5, 0.7, 0.8, and 1.0.

Response to Comment 5:

According to the article [3], various pairs of (C, γ) values were tried and the ones yielding the best cross-validation accuracy were chosen. Good results were obtained with exponentially growing sequences of C and γ, and that is why we initially tried C= $10^0, 10^1$, $10^2, 10^3$ and γ = $10^{-4}, 10^{-5}, 10^{-6}, 10^{-7}$. When we fine-tuned the parameters C and γ in these ranges, the experimental results showed that C =10, 100 and γ = $10^{-5}, 10^{-6}$ produce the highest classification accuracies. Then, we randomly changes the value of C within the range [10, 100] and γ within the range [$10^{-6}, 10^{-5}$] in order to optimise the accuracy.

The reason why we chose a coefficient k was discussed in the following paragraph (lines 328 to 336): "Due to the high bin values in the FLBP method, as shown in **Error! Reference source not found.** and **Error! Reference source not found.**, cmask_features are scaled by multiplying pass_features by coefficient k in k-FLBPCM method. For example, **Error! Reference source not found.** shows the distributions of patterns (bin values) in a typical canola image. It demonstrates that by combining the pass_features (in FLBP method) and cmask_features (in FBLPbCM method), the bin values of the k-FLBPCM method have better balance between these two feature sets. The purpose of multiplying coefficient k (k≤1) with pass_features is to reduce the gap between the bin values of the cmask_features and pass_features." Moreover, we selected k randomly from 0.1 to 1 and tested all these values in the experiments in order to observe the variation of values and chose an optimal set k, C and Gamma when these parameters reach the highest classification accuracy.

Comment 6: Line 310: Fig. 2.4 (a-d),…where is this figure, I didn't found it.

Response to Comment 6: We thank the Reviewer for pointing out some typos, which have now been corrected. We have modified all Figures using similar formats throughout the manuscript. In addition, we have highlighted all the corrections in our revised manuscript.

References

1. Kalinin AA, Allyn-Feuer A, Ade A, Fon G-V, Meixner W, Dilworth D, et al. 3D shape modeling for cell nuclear morphological analysis and classification. Scientific reports. 2018;8 1:1-14.
2. Chen S, Zhao M, Wu G, Yao C and Zhang J. Recent advances in morphological cell image analysis. Computational and mathematical methods in medicine. 2012;2012.

3. Hsu C-W, Chang C-C and Lin C-J. A practical guide to support vector classification. Taipei, 2003.

Personal Cover Click here to access/download;Personal Cover;Covering Letter.docx

Vi Nguyen Thanh Le
Electron Science Research Institute
Edith Cowan University
Joondalup Drive, WA 6027
Email: vlenguye@our.ecu.edu.au

Editor-in-Chief
Journal of GigaScience

October 15, 2019

Dear Editors,

I am submitting a manuscript for consideration of publication in the Journal of GigaScience. The manuscript is entitled "A novel k-FLBPCM method for detecting morphologically similar crops and weeds based on the combination of contour masks and Local Binary Pattern operators".

We propose a novel method for classifying plants with similar morphology. By investigating optimal parameters, this method reached a classification accuracy of 98.63% with four classes in the "bccr-segset" dataset in comparison with an accuracy of 91.85% attained by a previously reported method.

On behalf of all the authors, I confirm that this manuscript has not been published elsewhere and is not under consideration by another journal. In addition, we have no conflicts of interest to disclose.

Thank you very much for your consideration.

Yours Sincerely,

Vi Nguyen Thanh Le
Electron Science Research Institute
Edith Cowan University

Joondalup Drive, WA 6027

Email: vlenguye@our.ecu.edu.au or thviln88@gmail.com
