# Supplementary material for: A novel method for detecting morphologically similar crops and weeds based on the combination of contour masks and filtered Local Binary Pattern operators
Source: Gigascience. 2020 Mar 4;9(3):giaa017. doi: 10.1093/gigascience/giaa017 (PMC7055473; doi:10.1093/gigascience/giaa017)
Supplement: giaa017_Response_to_Reviewer_Comments_Original_Submission [file giaa017_response_to_reviewer_comments_original_submission.pdf]

Author's Response To Reviewer Comments

Close

RESPONSE TO REVIEWERS

We would like to thank the Reviewers for your valuable and detailed comments. We have gone through and seriously cons

Editor’s comment: Please register any new software application in the bio.tools and SciCrunch.org databases to receive RF
tracking, reproducibility and re-use of your tool.
Response: We have registered an RRID account for our algorithm on SciCrunch. The RRID for this resource is: k-FLBPCM-

Reviewer 1’s comments:
Comment 1: Do the authors consider the reuse potential of the k-FLBPCM method limited to classifying plant images? Alte
have relatively similar morphology? A "reuse potential" section outlining the value of the k-FLBPCM method would be a us
Response to Comment 1: We thank the Reviewer for your suggestion. We have now added a section in The Conclusion sec
order to identify objects of similar morphologies. Morphological cell analysis plays a significant role in supporting patholog
morphological features and identifying abnormal cells.”

Comment 2: The GitHub archive (https://aus01.safelinks.protection.outlook.com/?url=https%3A%2F%2Fgithub.com%2F
method&data=02%7C01%7Cvlenguye%40our.ecu.edu.au%7C69e0e0c2143a453e644508d79d7c7421%7C9bcb323d7fa3
does not appear to have a license associated with it. Will the authors be ascribing an OSI-approved license to this GitHub
Response to Comment 2: We thank the Reviewer for your advice. We have added a General Public License (GPL) to our pr

Comment 3: To encourage reuse of the image data, I recommend that the bccr-segset dataset is archived in the GigaScie
Comment 4: To encourage reuse of the image data, I recommend that the can-rad dataset is archived in the GigaScience
Response to Comments 3 and 4: Since the data has already been an open-source dataset, you can archive the bccr-segse

Reviewer 2’s comments:
Comment 1: The methods used for investigation which includes LBP variants, Morphological processing and SVM Classifier
Comment 2: Conclusions are adequately supported by the experimental results.
Comment 4: Minor language editing may be possible. It does not require a heavy editing for language and clarity.
Response to Comments 1, 2 and 4: We thank the Reviewer for your comments.

Comment 3: Quality of language in the manuscript is good. However grammatical corrections are needed at some lines as
some differences between'.
Response to Comment 3: We thank the Reviewer. We have corrected all grammatical errors, as requested.

Comment 5: Reason behind selecting following statistical parameters should be mentioned: C =1, 10, 30, 60, 100, 1000,
Response to Comment 5:
According to the article [3], various pairs of (C, γ) values were tried and the ones yielding the best cross-validation accura
〖10〗^0,〖10〗^1, 〖10〗^2,〖10〗^3 and γ =〖 〖10〗^(-4),〖10〗^(-5),10〗^(-6),〖10〗^(-7). When we fine-tuned the parameters C
accuracies. Then, we randomly changes the value of C within the range [10, 100] and γ within the range [〖 〖10〗^(-6),10〗
The reason why we chose a coefficient k was discussed in the following paragraph (lines 328 to 336): “Due to the high bir
FLBPCM method. For example, Table 1 shows the distributions of patterns (bin values) in a typical canola image. It demor
method have better balance between these two feature sets. The purpose of multiplying coefficient k (k≤1) with pass_fea
1 and tested all these values in the experiments in order to observe the variation of values and chose an optimal set k, C

Comment 6: Line 310: Fig. 2.4 (a-d),…where is this figure, I didn't found it.
Response to Comment 6: We thank the Reviewer for pointing out some typos, which have now been corrected. We have r
manuscript.

References
1. Kalinin AA, Allyn-Feuer A, Ade A, Fon G-V, Meixner W, Dilworth D, et al. 3D shape modeling for cell nuclear morphologi
2. Chen S, Zhao M, Wu G, Yao C and Zhang J. Recent advances in morphological cell image analysis. Computational and r

3. Hsu C-W, Chang C-C and Lin C-J. A practical guide to support vector classification. Taipei, 2003.

Close
