# Supplementary material for: A novel method for detecting morphologically similar crops and weeds based on the combination of contour masks and filtered Local Binary Pattern operators
Source: Gigascience. 2020 Mar 4;9(3):giaa017. doi: 10.1093/gigascience/giaa017 (PMC7055473; doi:10.1093/gigascience/giaa017)
Supplement: giaa017_Reviewer_1_Report_Original_Submission — Chris Armit -- 10/28/2019 Reviewed [file giaa017_reviewer_1_report_original_submission.pdf]

Reviewer Report

Title: A novel k-FLBPCM method for detecting morphologically similar crops and weeds based on the combination of contour masks and Local Binary Pattern operators

Version: Original Submission **Date:** 10/28/2019

Reviewer name: Chris Armit

Reviewer Comments to Author:

In this Research article, the authors utilise contour masks and morphological operators to classify canola and radish leaves using a k-FLBPCM method. The k-FLBPCM method - a filtered local binary pattern (LBP) method with contour mask and coefficient k - is shown to discriminate between broadleaf plants species of relatively similar morphology. For the bccr-segset dataset - which included 30,000 plant images in four classes (canola, corn, radish and background) under different rotations, scales and illumination conditions - the classification accuracy was 98.63%, which is superior to the average classification accuracies previously reported for FLBP and FLBPCM methodologies. This use case clearly highlights the utility of the k-FLBPCM classifier in correctly distinguishing between morphologically similar crops and weeds. The authors additionally explored the effects of contour thickness on classification accuracy of the can-rad dataset of 19600 broadleaf images (9800 images in each class), and reported on the parameters associated with optimal discrimination between canola and radish leaves. The manuscript is well written, and the authors are to be commended for providing a robust description of this classifier.
Minor comments
As GigaScience has a strong focus on reuse potential of data and software, I invite the authors to address the following comments.
1. Do the authors consider the reuse potential of the k-FLBPCM method limited to classifying plant images? Alternatively, could this method be used in a Digital Pathology context to identify between, for example, epithelial and mesenchymal cells that have relatively similar morphology? A "reuse potential" section outlining the value of the k-FLBPCM method would be a useful addition to the manuscript for future researchers.
2. The GitHub archive (https://github.com/vinguyenle/k-FLBPCM-method) does not appear to have a license associated with it. Will the authors be ascribing an OSI-approved license to this GitHub archive?
3. To encourage reuse of the image data, I recommend that the bccr-segset dataset is archived in the GigaScience DataBase (GigaDB). Does GigaScience have permission to archive the bccr-segset image dataset?
4. To encourage reuse of the image data, I recommend that the can-rad dataset is archived in the GigaScience DataBase (GigaDB). Does GigaScience have permission to archive the can-rad image dataset?

Methods

Are the methods appropriate to the aims of the study, are they well described, and are necessary controls included? Choose an item.

Conclusions

Are the conclusions adequately supported by the data shown? Choose an item.

Reporting Standards

Does the manuscript adhere to the journal's guidelines on minimum standards of reporting? Choose an item.

Choose an item.

Statistics

Are you able to assess all statistics in the manuscript, including the appropriateness of statistical tests used? Choose an item.

Quality of Written English

Please indicate the quality of language in the manuscript: Choose an item.

Declaration of Competing Interests

Please complete a declaration of competing interests, considering the following questions:

- Have you in the past five years received reimbursements, fees, funding, or salary from an organisation that may in any way gain or lose financially from the publication of this manuscript, either now or in the future?
- Do you hold any stocks or shares in an organisation that may in any way gain or lose financially from the publication of this manuscript, either now or in the future?
- Do you hold or are you currently applying for any patents relating to the content of the manuscript?
- Have you received reimbursements, fees, funding, or salary from an organization that holds or has applied for patents relating to the content of the manuscript?
- Do you have any other financial competing interests?
- Do you have any non-financial competing interests in relation to this paper?

If you can answer no to all of the above, write 'I declare that I have no competing interests' below. If your reply is yes to any, please give details below.

I declare that I have no competing interests.

I agree to the open peer review policy of the journal. I understand that my name will be included on my report to the authors and, if the manuscript is accepted for publication, my named report including any attachments I upload will be posted on the website along with the authors' responses. I agree for my

report to be made available under an Open Access Creative Commons CC-BY license (http://creativecommons.org/licenses/by/4.0/). I understand that any comments which I do not wish to be included in my named report can be included as confidential comments to the editors, which will not be published.

Choose an item.

To further support our reviewers, we have joined with Publons, where you can gain additional credit to further highlight your hard work (see: https://publons.com/journal/530/gigascience). On publication of this paper, your review will be automatically added to Publons, you can then choose whether or not to claim your Publons credit. I understand this statement.

Yes Choose an item.
