# Supplementary material for: A novel method for detecting morphologically similar crops and weeds based on the combination of contour masks and filtered Local Binary Pattern operators
Source: Gigascience. 2020 Mar 4;9(3):giaa017. doi: 10.1093/gigascience/giaa017 (PMC7055473; doi:10.1093/gigascience/giaa017)
Supplement: giaa017_Reviewer_2_Report_Original_Submission — Jayamala Patil -- 1/20/2020 Reviewed [file giaa017_reviewer_2_report_original_submission.pdf]

Reviewer Report

Title: A novel k-FLBPCM method for detecting morphologically similar crops and weeds based on the combination of contour masks and Local Binary Pattern operators

Version: Original Submission **Date:** 1/20/2020

Reviewer name: Jayamala Patil

Reviewer Comments to Author:

1. The methods used for investigation which includes LBP variants , Morphological processing and SVM Classifier are well described.
2. Conclusions are adequately supported by the experimental results.
3. Quality of language in the manuscript is good. However grammatical corrections are needed at some lines as below:
Line 97: correct sentence grammatically.
Line 246: 'However, there is some differences between': it may be: However, 'there are some differences between'.
4. Minor language editing may be possible. It does not require a heavy editing for language and clarity.
5. Reason behind selecting following statistical parameters should be mentioned :
c =1, 10, 30, 60, 100, 1000, Î³ =â�¡10âˆ’4, 10âˆ’5, 10âˆ’6, 10âˆ’7and k =0.1,384 0.2, 0.5, 0.7, 0.8, and 1.0.
6. Line 310: Fig. 2.4 (a-d),…where is this figure, I didn't found it.

Methods

Are the methods appropriate to the aims of the study, are they well described, and are necessary controls included? Choose an item.

Conclusions

Are the conclusions adequately supported by the data shown? Choose an item.

Reporting Standards

Does the manuscript adhere to the journal's guidelines on [minimum standards of reporting?] Choose an item.

Choose an item.

Statistics

Are you able to assess all statistics in the manuscript, including the appropriateness of statistical tests used? Choose an item.

Quality of Written English

Please indicate the quality of language in the manuscript: Choose an item.

Declaration of Competing Interests

Please complete a declaration of competing interests, considering the following questions:

- Have you in the past five years received reimbursements, fees, funding, or salary from an organisation that may in any way gain or lose financially from the publication of this manuscript, either now or in the future?
- Do you hold any stocks or shares in an organisation that may in any way gain or lose financially from the publication of this manuscript, either now or in the future?
- Do you hold or are you currently applying for any patents relating to the content of the manuscript?
- Have you received reimbursements, fees, funding, or salary from an organization that holds or has applied for patents relating to the content of the manuscript?
- Do you have any other financial competing interests?
- Do you have any non-financial competing interests in relation to this paper?

If you can answer no to all of the above, write 'I declare that I have no competing interests' below. If your reply is yes to any, please give details below.

I declare that I have no competing interests.

I agree to the open peer review policy of the journal. I understand that my name will be included on my report to the authors and, if the manuscript is accepted for publication, my named report including any attachments I upload will be posted on the website along with the authors' responses. I agree for my report to be made available under an Open Access Creative Commons CC-BY license (http://creativecommons.org/licenses/by/4.0/). I understand that any comments which I do not wish to be included in my named report can be included as confidential comments to the editors, which will not be published.

Choose an item.

To further support our reviewers, we have joined with Publons, where you can gain additional credit to further highlight your hard work (see: https://publons.com/journal/530/gigascience). On publication of this paper, your review will be automatically added to Publons, you can then choose whether or not to claim your Publons credit. I understand this statement.

Yes Choose an item.
